# Extending Vulnerability Assessment to Include Life Stages Considerations

**DOI:** 10.1371/journal.pone.0158917

**Published:** 2016-07-14

**Authors:** Emma E. Hodgson, Timothy E. Essington, Isaac C. Kaplan

**Affiliations:** 1 School of Aquatic and Fishery Sciences, University of Washington, Box 355020, Seattle, WA, 98195, United States of America; 2 Conservation Biology Division, Northwest Fisheries Science Center, National Marine Fisheries Service NOAA, 2725 Montlake Blvd., E Seattle, WA, 98112, United States of America; University of Waikato (National Institute of Water and Atmospheric Research), NEW ZEALAND

## Abstract

Species are experiencing a suite of novel stressors from anthropogenic activities that have impacts at multiple scales. Vulnerability assessment is one tool to evaluate the likely impacts that these stressors pose to species so that high-vulnerability cases can be identified and prioritized for monitoring, protection, or mitigation. Commonly used semi-quantitative methods lack a framework to explicitly account for differences in exposure to stressors and organism responses across life stages. Here we propose a modification to commonly used spatial vulnerability assessment methods that includes such an approach, using ocean acidification in the California Current as an illustrative case study. Life stage considerations were included by assessing vulnerability of each life stage to ocean acidification and were used to estimate population vulnerability in two ways. We set population vulnerability equal to: (1) the maximum stage vulnerability and (2) a weighted mean across all stages, with weights calculated using Lefkovitch matrix models. Vulnerability was found to vary across life stages for the six species explored in this case study: two krill–*Euphausia pacifica* and *Thysanoessa spinifera*, pteropod–*Limacina helicina*, pink shrimp–*Pandalus jordani*, Dungeness crab–*Metacarcinus magister* and Pacific hake–*Merluccius productus*. The maximum vulnerability estimates ranged from larval to subadult and adult stages with no consistent stage having maximum vulnerability across species. Similarly, integrated vulnerability metrics varied greatly across species. A comparison showed that some species had vulnerabilities that were similar between the two metrics, while other species’ vulnerabilities varied substantially between the two metrics. These differences primarily resulted from cases where the most vulnerable stage had a low relative weight. We compare these methods and explore circumstances where each method may be appropriate.

## Introduction

Human activities are altering ecosystems across the globe at historically unprecedented rates. Predicting the consequence of these alterations for species, ecosystem structure and ecosystem resilience is essential to planning for the impacts of environmental change [1, 2]. However, in many systems and for many species, there is a lack of data available to provide quantitative predictions of changing system states under new pressures [3]. For this reason, a variety of qualitative and semi-quantitative methods have been developed to guide decision makers about likely futures [4–6]. A common feature of many assessment tools is that they seek to evaluate the vulnerability of a species, ecosystem or human community to changing anthropogenic pressures (e.g., [7–10]). Vulnerability in this case is “the degree to which a system is susceptible to and is unable to cope with adverse effects” [11] and largely developed out of an interest in understanding linked human-ecological systems [12]. Three components are common to vulnerability assessments: *exposure*, *consequence from exposure* and *resilience* [9, 12, 13], which are used to assess *current* vulnerability of species or ecosystems to stressors [14, 15] or to assess *future* vulnerability under stressors like climate change [10, 16].

To understand species vulnerability to new or existing stressors, we need to consider which life stages are likely to be vulnerable. For some stressors there is clearly an impact directed towards a single life stage (e.g., fishing of adults), while other stressors might affect multiple life stages (e.g., pollution or climate change). As vulnerability is a result of exposure to a stressor and the consequence of exposure, we may observe differences in life stage vulnerability due to varying degrees of stressor exposure and/or consequence. Many organisms exhibit distinct spatio-temporal distributions throughout their ontogeny [17, 18], influencing the stressors they overlap with and possibly creating opportunities for natural avoidance of, or exposure to, stressors. Early life stages are often found to be particularly susceptible to chemical or physical stressors [19–23] making their potential consequence high upon exposure. Consequently, vulnerability is likely to change through life [24], and we need a way to identify the range in vulnerability across life stages and use that to inform population vulnerability.

For stressors that potentially impact species at multiple life stages, there is a major challenge in translating individual life stage vulnerabilities into a population vulnerability estimate. Population vulnerability could equal that of the most vulnerable life stage, or it could be some integration measure across the stages. If all life stages experience a similar vulnerability, then either approach will reach the same conclusion. However, if only one life stage experiences high vulnerability, and the rest experience low vulnerability, the implications of the stressor for the population is unclear. This is particularly the case if the most vulnerable stage is relatively unimportant from a demographic perspective (e.g., low reproductive value [25]). For example, in sea turtles the early life stages have a lower relative contribution to population growth and protecting later life stages is relatively more important [26]; however, for many species early life stages are more susceptible to stressors [23, 27]. This begs the question, “when does a highly vulnerable early life history stage translate into a highly vulnerable population?” Information provided by vulnerability assessment does not tell us how important each individual life stage is for the population or how the vulnerability translates to a quantitative change in a parameter value (e.g. survival, fecundity or growth).

Building on a rich history of risk and vulnerability assessment methodologies [9, 12, 13, 28–30] here we adapt semi-quantitative spatial vulnerability assessment to incorporate life stage considerations [25, 26], using the case study of ocean acidification (OA) in the California Current. An approach that explicitly and transparently addresses changing vulnerability as organisms move between environments and stages within their life cycle has been called for in the vulnerability assessment literature [24]. The California Current is a useful case study for these investigations as it is naturally prone to low pH and lower levels of aragonite saturation, with distinct spatial and seasonal patterns of pH [31]. This will likely make exposure change among life stages that inhabit diverse depth, longitudinal and latitudinal ranges, and we know from laboratory studies that responses from exposure to low pH vary between different life stages of an individual species [23, 27].

We explicitly estimate vulnerability at all life stages for six species within the California Current. We then use these life stage vulnerability estimates to determine overall population vulnerability by comparing two metrics; the first metric assumes population vulnerability is equal to the vulnerability of the most vulnerable stage and the second integrates the vulnerabilities across all stages using a weighted approach. These two metrics were chosen because each might be useful in a different context. Using this process, we investigate the questions: (1) does vulnerability vary across life stages? (2) How do two these alternative approaches for evaluating population vulnerability compare when vulnerability does vary across stages? We use this exercise to reveal the benefits and limitations of these alternative approaches to confronting the challenge of life-stage specific vulnerability and determine vulnerability to ocean acidification for six ecologically and/or economically important species found in the California Current. Our method also directly assesses *confidence* in the vulnerability estimates (similar to the IPCC approach [32]), given the limited information we often have regarding particular stressor-response pairings [14].

## Methods

**Fig 1:**
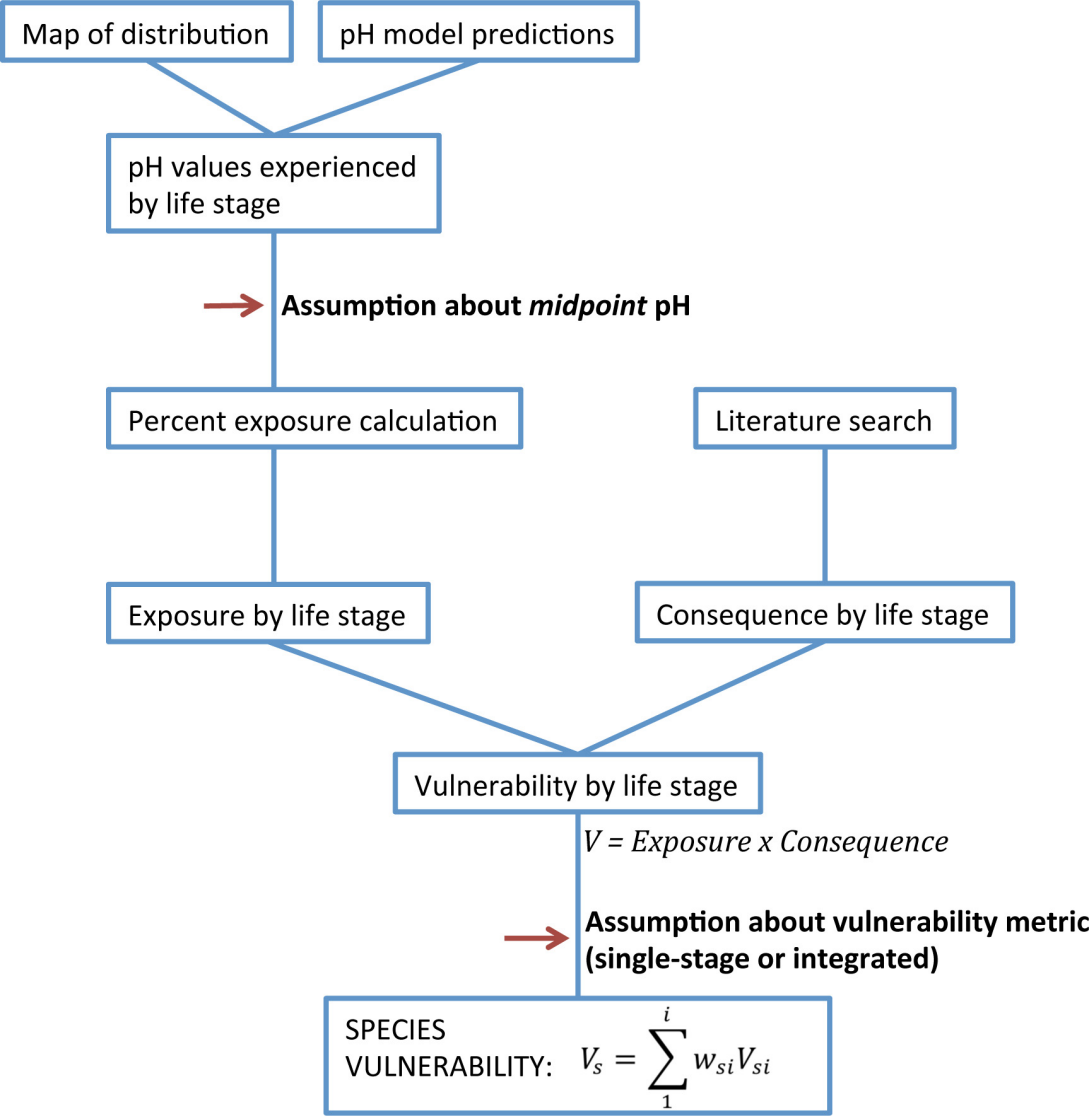
Conceptual diagram of vulnerability assessment components. Diagram includes steps in the vulnerability assessment—exposure and consequence scores that determine stage vulnerability. Red arrows indicate key locations where assumptions are made in the final calculation.

For six example species we calculated vulnerability across the main life history stages and used these estimates to determine population vulnerability. Stage vulnerability was the product of exposure to low pH and consequence from exposure (Fig 1), each having a score from 1–3 (similar to previous methods [33]). Population vulnerability was calculated two ways: (1) we assumed population vulnerability was equal to the vulnerability estimate of the most vulnerable stage, and (2) population vulnerability was calculated as a weighted mean across all stages. In the latter approach, the weights were proportional to the relative importance of each stage for population growth rate, determined from stage-structured models developed for each species. Uncertainty was determined for each element included in the vulnerability estimates.

Using the case study of ocean acidification in the California Current, species were selected for assessment based on ecological importance, economic value, and/or presumed sensitivity to OA. OA sensitivity was based on distributions in low pH areas or demonstrated biological response to low pH. Species were also chosen to include a variety of taxonomic groups at different levels in the food web: pteropod, krill, shrimp, crab and fish (Table 1).

**Table 1:** Species included in the vulnerability assessment, the reason for their inclusion and the life stages assessed.

Species *(Scientific and common names)*	Reasons for Inclusion	Life History Stages Included
Pacific hake, *Merluccius productus*	$	Eggs, larvae and adults
Dungeness crab, *Metacarcinus magister*	$, S	Eggs, zoea larvae, megalopal larvae, juveniles and adults
Pink shrimp, *Pandalus jordani*	$	Eggs, larvae, juveniles and adults
Krill, *Euphausia pacifica*	E, S	Eggs, larvae, juveniles, subadults and adults
Krill, *Thysanoessa spinifera*	E, S	Eggs, larvae, juveniles and adults
Pteropod, *Limacina helicina*	S	Eggs, juveniles, sub-adults and adults

E = ecologically important, $ = economically valuable fishery species, S = known or presumed consequence from lower pH.

The study region was the US exclusive economic zone (EEZ) off Oregon and California in the California Current system. This section of the coast was chosen because of available model predictions of future ocean acidification levels [31]. The California Current is an eastern boundary current that receives water with high dissolved inorganic carbon during the upwelling season in both spring and summer, bringing low pH water into the shallow and nearshore environments [31, 34]. Because the oceans absorb approximately one third of carbon emissions, ocean pH levels are declining globally [35–38]. For regions with naturally low pH such as the California Current, this can be particularly problematic because future declines may push pH beyond species’ physiological tolerance thresholds [31, 34, 39]. Moreover, this ecosystem exhibits wide seasonal variations in pH, which is relevant when considering exposure of life stages with distinct phenology that may coincide with or avoid low pH conditions.

### Life Stage Vulnerability

#### pH model

Spatio-temporal projections of ocean pH were derived from the previously published Regional Ocean Model Systems (ROMS) model for the California Current [31]. ROMS is a dynamic, three-dimensional model, developed as a multi-purpose marine modeling system that has proved successful in marine environments [40]. The ROMS model used provides outputs on a 5 km^2^ grid and uses present-day climatological boundary conditions, modifying only the carbon inputs to 2050 using the B1 and A2 IPCC pollution scenarios [31]. We used the outputs from the A2 highest emissions scenario for the year 2050, recognizing that this is a worst case scenario and allows us to assess the greatest possible vulnerability. The A2 scenario predicts 541 ppm partial pressure of CO_2_ by the year 2050, increasing from 280 ppm in pre-industrial times [31]. This model is currently the most state-of-the-art for predicting oceanographic conditions in the California Current, resolving upwelling which is not well-resolved using the global scale models [31, 36]. Although upwelling is well-resolved, we use model outputs at the monthly-average pH scale, which does not include natural pH fluctuations which occur on a much shorter time scale in the California Current [41]. It is likely that inclusion of daily fluctuations would lead to species being exposed to lower pH values than those given by a monthly average, however, smaller scale exposure calculations are not within the scope of this paper.

To be consistent across the species included in this assessment, we used pH as the representative variable for acidification. Although aragonite saturation state may be more important for pteropods, using different variables for each species would require additional assumptions. Instead, we used pH as a proxy for aragonite saturation state to maintain consistency across species. The values of pH used to represent exposure (7.6–7.7) correspond to aragonite near or below its saturation state in the California Current, as it has been shown that a pH<7.75 is associated with aragonite saturation < 1.0 [34].

#### Mapping Distributions

Species distributions were mapped to address distinct spatial and temporal distributions throughout an organism’s life. We created presence/absence maps for each month, species, and life history stage. We based these maps on survey data when available, or information collected through a literature review and consultation with experts. Most maps were created using the latter method. Each map was developed as a 2D polygon representing the presence of a species’ life history stage for a given month of the year. We identified the deepest depth that the organism inhabits, and based exposure on pH levels at this depth. Because pH generally declines with depth, we were essentially calculating maximum potential exposure. Species distributions were assumed to remain the same between now and 2050. Although species distributions may shift in response to temperature and pH, we do not have the ability to precisely predict these shifts; we therefore make a simplifying assumption that distributions in 2050 will be roughly similar to those in present day. That is, our assessment of vulnerability is contingent on a stable spatial structure to populations.

Maps for Dungeness crab, pink shrimp, two krill species and pteropods were all developed using the literature review and expert consultation method. Preliminary distributions were determined using the literature. For most species (Dungeness crab, krill and pink shrimp), there was information on several life stages (adults, juveniles and eggs) to inform these distributional maps. For pteropods and the planktonic larval stages of these species, preliminary maps were based on limited information with high uncertainty. The preliminary map assumptions were then sent to experts for revisions, leading to final maps that were input into ArcGIS (version 10.2). Details on sources for all species maps can be found in the Supporting Information (Table A1 in Appendix A in S1 File) with figures of maps (Figures A1-38 in Appendix A in S1 File).

We used survey data to describe distributions of Pacific hake. These data came from CalCOFI hake surveys during 1984–2012 [42], which contained data on egg and larval abundance at discrete survey locations. CalCOFI data were only used for years when greater than 500 individuals were collected, and we disregarded tows with fewer than 5 individual hake eggs or larvae. A convex hull was then created for monthly maps in ArcGIS. Note that we only mapped the distribution of Pacific hake eggs and larvae as there is no indication that adult teleost fish are impacted by OA. Therefore adult Pacific hake exposure and consequence were assumed to equal 1, the minimum level. This was reasonable given that there is little evidence of direct adverse consequences of OA adult stages of teleost fish [43, 44].

#### Calculating Exposure

**Fig 2:**
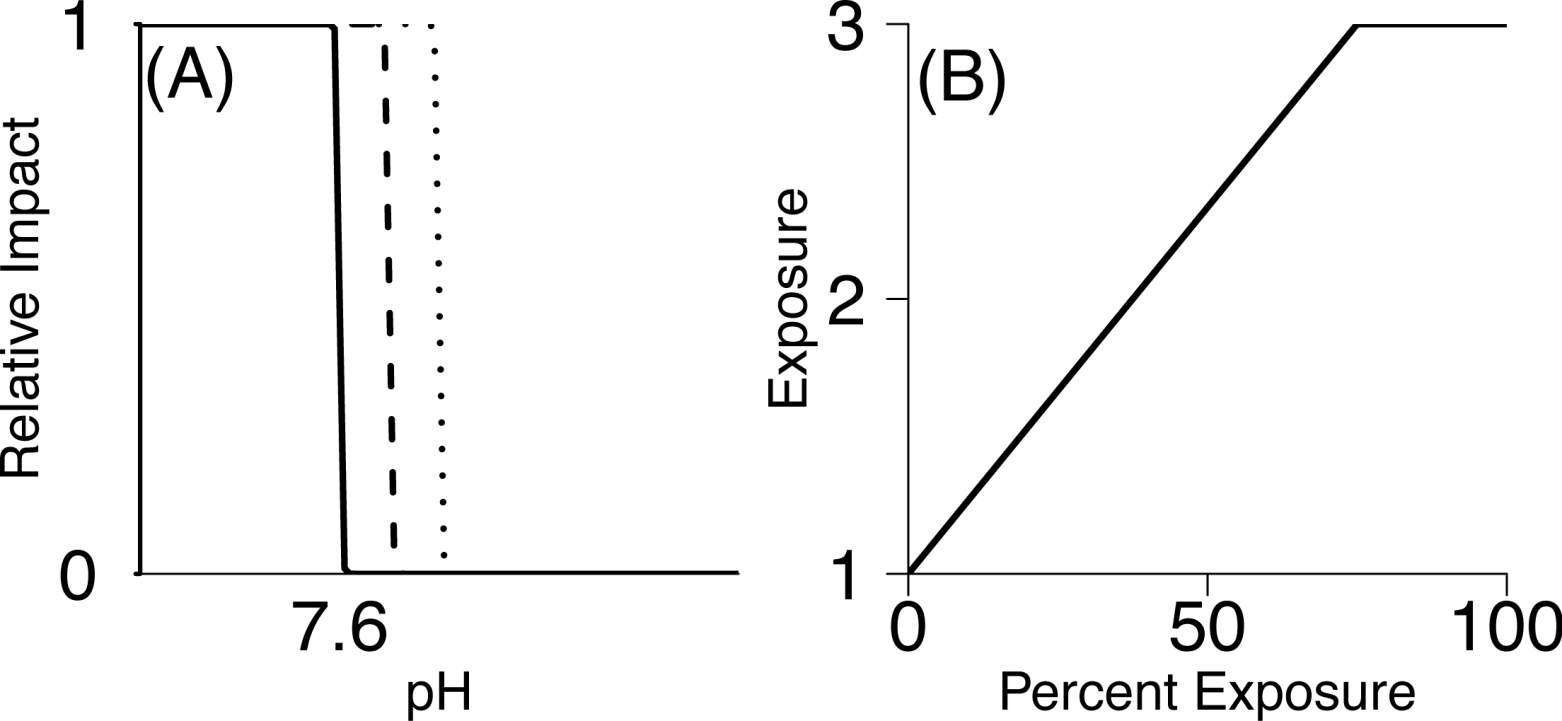
Method for translating pH level and percent exposure into exposure scores. (A) Relative impact vs. pH for the three threshold values of 7.6, 7.65 and 7. (B) Scaling used to relate percent exposure to final exposure score.

To calculate vulnerability, we had to define conditions that would be considered high exposure (pH that produces maximum consequence). The pH experienced by each species’ life stage was determined by overlaying life stage maps with pH predictions [31] using ArcGIS. This produced a pH value for each 5 km^2^ grid cell within the life stage’s distributional range for each month of the year. Ultimately we were interested in generating a single exposure score to use in the semi-quantitative ranking of vulnerability, yet species are exposed to a gradient of pH levels. In other words, pH is a continuous variable and consequences of exposure to different pH values might also be continuous. Data needed to parameterize detailed, continuous stressor-response curves generally were not available. For this reason, we chose a simpler representation and an accompanying sensitivity analysis to measure vulnerability. We used a step function, assuming minimal adverse effects above a threshold pH level and maximum pH effects below the threshold (we tested the assumption of the shape of this ‘threshold’ curve against curves that take a more gradual sigmoidal shape–see Appendix B in S1 File for more information). We considered three threshold pH values to account for uncertainty: 7.6, 7.65, and 7.7 (Fig 2). These threshold pH values were chosen as they are the range within which negative consequences of pH are believed to occur, and are frequently used as the experimental ‘low pH’ value [43]. While similar to stressor-response curves in environmental toxicology [45, 46], our stressor-response step functions were derived from assumed relationships rather than direct measurements.

Using the threshold approach, for each species’ life stage, each grid cell within the distribution of that life stage was given a value of 0 or 1, indicating whether pH was above or below the threshold, respectively. This was done for all 12 months of the year (or for the months that life stage is found in the water column). We calculated the *percent exposure* to low pH as the percent of grid cells scored as a 1 (pH less than the threshold) out of all grid cells within the life stage’s distribution throughout the year. Percent exposure was then translated into an Exposure value between 1–3 assuming that any percent exposure greater than 75% was considered *high exposure* (3), and values between 0% and 75% were linearly converted to values between 1–3 (Fig 2B). Although the relationship between percent exposure and the exposure score also involves potentially influential assumptions, these were necessary to simplify the framework and to focus on life-history-specific vulnerability. Previous authors have assumed similar relationships but have placed high exposure as anything above 50% [47]. However, because in the California Current species are already experiencing pH around 7.6 [34], and our threshold pH of 7.6–7.7 is within the range of what some species may currently experience, we chose a higher end of >75% exposure to rank as ‘high’ exposure (3).

#### Consequence

The response of each organism to ocean acidification was determined through a combination of literature review and consultation with experts. For each species included in this analysis, we used the search engines Scopus, Web of Science and Google Scholar to identify papers on the species’ response to low pH and low aragonite saturation. Research conducted on our species of interest was used when available; however for some species we used other species in the same genus. For Pacific hake, research on other teleost fishes was used. Additionally, we used recent unpublished investigations (see Appendix C in S1 File) for the krill *Euphausia pacifica*.

Consequence was defined as whether the organism’s life stage demonstrates an ability to tolerate exposure. We have used ‘consequence’ as opposed to the commonly used term ‘sensitivity’ [12] since ‘sensitivity’ has numerous definitions depending on the field. In this paper we only use sensitivity in the sense of how assessment outputs depend upon input assumptions. There are a number of ways that organisms respond to ocean acidification: changing development times [48], increased or decreased survival [49, 50], changes in calcification [51] and changing response to predation [50]. As not all papers used the same experimental conditions or measured the same organismal responses, the purpose of this review was not to comprehensively assess whether increased developmental times are more or less detrimental to an organism than changes in survival. Rather, the review was used to determine more broadly whether lethal, sublethal or no effects were found for the study organism in response to OA. Thus, the literature was used to categorize a life stage consequence (C) from low 1 to high 3 (see Table 2). We assigned consequence score 1 when all the evidence indicated that exposure had no impact on either development or survival. A consequence score 2 was assigned if evidence indicated moderate but sublethal effects, while a consequence score 3 was assigned if evidence indicated a strongly adverse impact (e.g., shell dissolution and clear increases in mortality). Most studies tested the response of organisms at pH values near to the threshold pH values we used to determine exposure (Table C1 in Appendix C in S1 File).

**Table 2:** Description of three consequence scores (1–3) used to determine consequence from exposure to low pH.

Consequence	Category Description
1	Life stage demonstrates tolerance to exposure to low pH. Conclusion is based on direct experimentation on this or very closely related species, or based on known exposure patterns in sustained populations.
2	Life stage demonstrates some tolerance to exposure to low pH: evidence suggests limited, but not full, tolerance. Empirical evidence shows some effect, but effect size is moderate. Evidence may come from this or from a related species or similar life stage.
3	Life stage shows clear impact from exposure to low pH. This may be based on demonstration of direct effects on this or closely related species.

#### Stage Vulnerability

The vulnerability (*V*) of each life stage *i* was calculated multiplicatively: *V*_*i*_ = (*E*_*i*_) x (*C*_*i*_) similar to previous methods [14], where *E* is exposure and *C* is consequence. Given values of *E* and *C* ranging between 1 and 3, the vulnerability for each life stage lies between 1 and 9. In the results we discuss the range in life stage vulnerability estimates within each species. Vulnerability range was calculated as the difference between the lowest and highest vulnerability estimates among all life history stages (note that since consequence and exposure are bounded by 1–3, Vulnerability is bounded by 1–9). We did not include *adaptive capacity* as a third axis to our vulnerability assessment [9] because of the limited information available for the early life stages of most species in this assessment. Other authors have made a similar decision based on the inherent challenges in scoring adaptive capacity [30].

### Population Vulnerability

We calculated population vulnerability using two methods. First, we assigned population vulnerability equal to the maximum of the stage-specific vulnerability estimates. Second, we calculated a weighted mean, where weights were derived from summed elasticity values from population models (described below): $V_{s}=\sum_{1}^{i}w_{si}V_{si}$. Hereafter these two methods are referred to as: maximum stage vulnerability and integrated vulnerability.

#### Life History Models and Life Stage Weights

Stage-structured models were developed for five of the six species included in this analysis: pink shrimp, Dungeness crab, Pacific hake and two krill species *E*. *pacifica* and *T*. *spinifera*. A model was not developed for *L*. *helicina* due to a paucity of published data on the survival and durations of the life stages of this species; therefore we averaged vulnerability across life stages with equal weightings to produce the integrative metric. For *T*. *spinifera*, although a model was developed, most of the parameters used came from research on *E*. *pacifica* and as a result we concluded high uncertainty in model estimates. The models were developed according to standard methods in life history matrix modeling [25, 52–54]. For example, for a species with three life stages where only the final stage reproduces, the Lefkovitch matrix has the form:

$$A=\left(\begin{array}{cc} P_{1} & \begin{array}{cc} 0 & F_{3} \end{array}\\ \begin{array}{c} G_{1}\\ 0 \end{array} & \begin{array}{cc} P_{2} & 0\\ G_{2} & P_{3} \end{array} \end{array}\right)$$

where *P*_*i*_ is the probability of remaining in stage *i* from one time step to the next, *G*_*i*_ is the transition probability and *F*_*i*_ is the fecundity of life stage *i*. The two probabilities *P*_*i*_ and *G*_*i*_ were calculated as:

$$P_{i}=s_{i}-G_{i}$$

$$G_{i}=\frac{{s_{i}}^{d_{i}}}{\sum_{t=1}^{t=d}{s_{i}}^{t-1}}$$

where *s*_*i*_ is the daily survival rate and *d*_*i*_ is the duration of the *i*th life stage, defined as the total number of days that a species will remain in stage *i*. This equation is subject to the assumption that the population growth rate, *λ* = 1 [25]. In this analysis, we were interested in the relative contribution to population growth that each life stage provides, not the ‘true’ population growth rate; thus, the assumption *λ* = 1 is appropriate. To ensure all models had a *λ* = 1, the matrix elements for each model were multiplied by a constant which varied between models (spanning 0.9714854–1.000133).

Survival rates and duration times for each stage were determined from the literature using published Lefkovitch matrices [55, 56], estimates from laboratory studies, or field based studies. Where possible, survival rates determined from field studies were preferred over laboratory estimates. Both methods result in limitations in the conclusion of true survival rates (for further discussion, see [57]); however, field based estimates were chosen as they include all sources of natural mortality. For the krill model, we wanted to address the fact that eggs can develop into spawning sub-adults after 4–7 months [58] which is dependent on the time of year when they are spawned (spring vs. summer; pers. comm. Julie Keister). We therefore include two ‘types’ for *E*. *pacifica*–early and late spawners, and calculated vulnerability separately for each type. However, results are only shown for the early spawning krill, as vulnerability was very similar for both types. Details on model parameters can be found in the Supporting Information (Appendix D in S1 File).

We then used the elasticity of population growth rate to each of the matrix elements as the basis of assigning weights to life history stages. Elasticities for the matrix elements were calculated using $e_{ij}=\frac{a_{ij}}{\lambda }\left(\frac{\partial \lambda }{\partial a_{ij}}\right)$ [25], where *a*_*ij*_ are the elements of matrix *A*. Each life stage weight equaled the sum of *e*_*ij*_ for all non-zero elements of A for the ith life stage, i.e., *w*_*i*_ = ∑_*j*_ *e_ij_*. Elasticities indicate how a proportional change in any stage-specific survival, transition, or offspring production affects population growth rate, and therefore provides a basis to identify the stages over which changes in demographic rates are likely to have the largest population-level effect. They provide insight into which life stage may be most important for overall population growth.

### Uncertainty

Uncertainty was assessed for both the exposure and consequence components of all life stages and combined to estimate overall population uncertainty. We used a semi-quantitative method because there was not sufficient information to quantify uncertainty more rigorously. The method estimates uncertainty in a similar manner to that used by the IPCC, scaling confidence to qualify the conclusions made [32]. Uncertainty of each element was scored between 1–3, based on criteria defined *a priori* (Table 3) similar to consequence scores, where 1 = low uncertainty and 3 = high uncertainty. Uncertainty in exposure, *Ue*_*i*_, was based on level of confidence in mapped distributions of each life stage *i*. Uncertainty in consequence, *Uc*_*i*_, for each life stage was based on confidence in conclusions from studies for each stage *i* (uncertainty values and reasoning can be found in Appendix E in S1 File). Total uncertainty for species *s* was calculated as the geometric mean of *Ue*_*i*_ and *Uc*_*i*_ for each life stage and summed using life history weights *w*_*i*_:

$$U_{s}=\sum_{1}^{i}{(\sqrt{{Ue}_{i}{Uc}_{i}})w}_{i}.$$

**Table 3:** Definitions used for uncertainty in exposure, from mapping distributions of species *U*_*e*_ and uncertainty in consequence *U*_*c*_.

*U*_*e*_	Definition
**1**	Low uncertainty in distribution: Map is based on multiple years of direct observations from surveys that span distributional range, distributions are consistent across years, or map is developed from conclusions found in numerous (3+) scientific papers and is supported by experts.
**2**	Moderate uncertainty in distribution: Distribution is based on minimal observations with some questionable accuracy of location or local conditions, full extent of distribution may not be precisely measured, or distribution is derived from model estimates.
**3**	High uncertainty in distribution: Distribution is based on conclusions from few papers (1–2) with minimal spatial coverage requiring generalizations to determine coast-wide distributions, even with expert confirmation of best estimate.
*U*_*c*_	
**1**	Low uncertainty in consequence conclusion: more than one study conducted directly on this species life stage, with agreement between studies.
**2**	Moderate confidence in consequence conclusion: one study conducted on this species, more than one but with conflicting results, or study conducted on a species of the same genus or on same species but different life stage.
**3**	High uncertainty in consequence conclusion: no studies directly on this species or any in the same genus, conclusions are on species in the same family.

## Results

**Fig 3:**
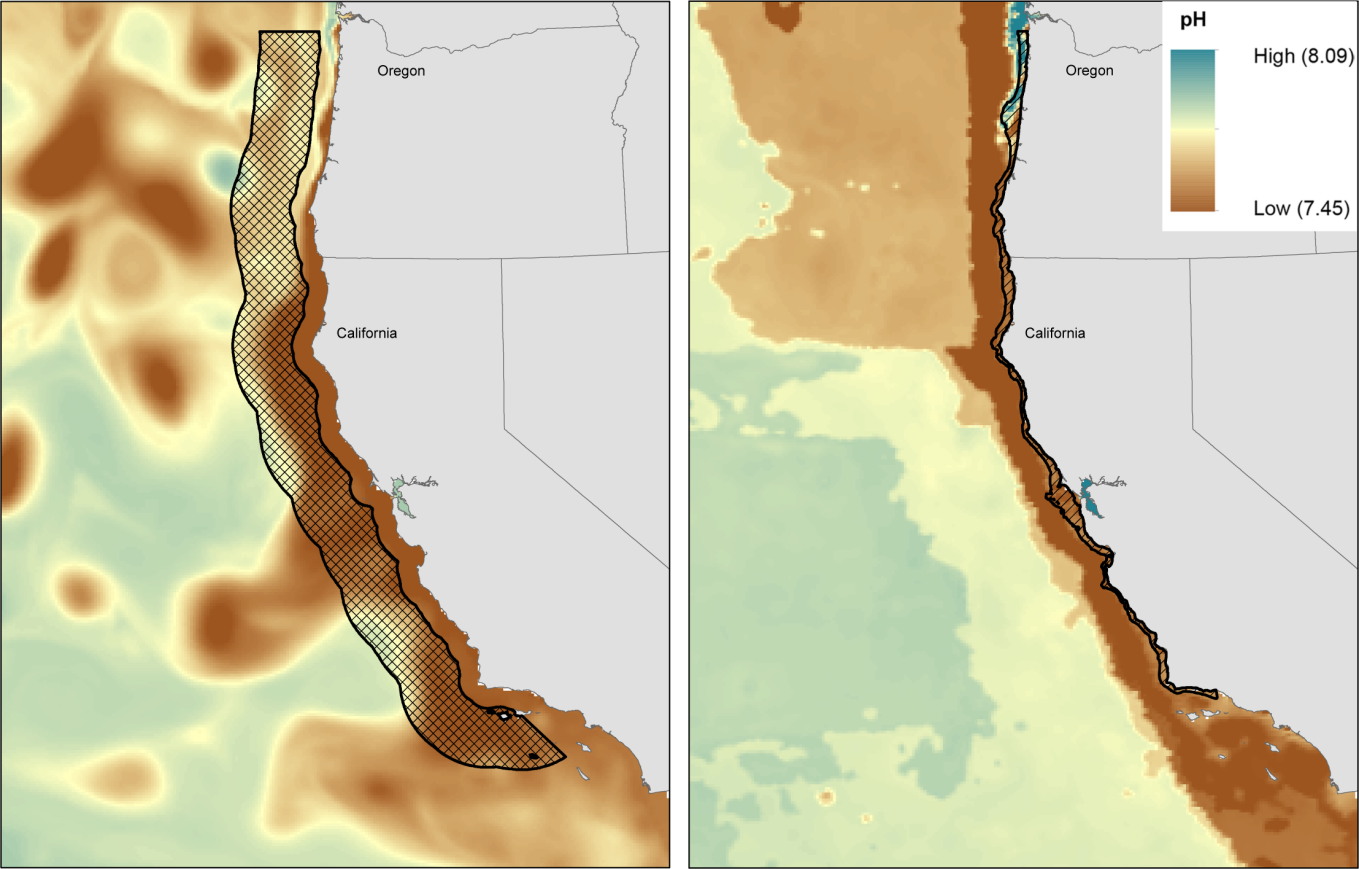
Dungeness crab larval, and adult and egg distributions with pH values for May 2050. (A) Larval distribution (checkered region with a black outline) with pH predictions at 70 m depth, and (B) adult and egg distributions (region identified with a black outline), with pH predictions along the sea floor.

Population distributions varied among life stages in time and space (latitude and longitude, and depth) and this variation led to different pH exposure through population life histories. For example, Dungeness crab adults inhabit a narrow band at the ocean bottom all months of the year (Fig 3). Because eggs are attached to females, eggs inhabit the same narrow band from October through March. In comparison, the larval stage is planktonic and is distributed in the upper water column further offshore (Fig 3). Consequently, adults and eggs have the highest exposure because pH is lowest along the bottom. Differences in exposure resulting from shifts in spatial distribution with age were found for all species (see Appendix A in S1 File for maps), with pteropods as the exception. With limited detailed knowledge on their distributions, the pteropods were assumed to inhabit a similar spatial extent throughout their life history. Temporal variability was therefore more important than spatial variability in governing exposure differences for pteropod life stages.

**Fig 4:**
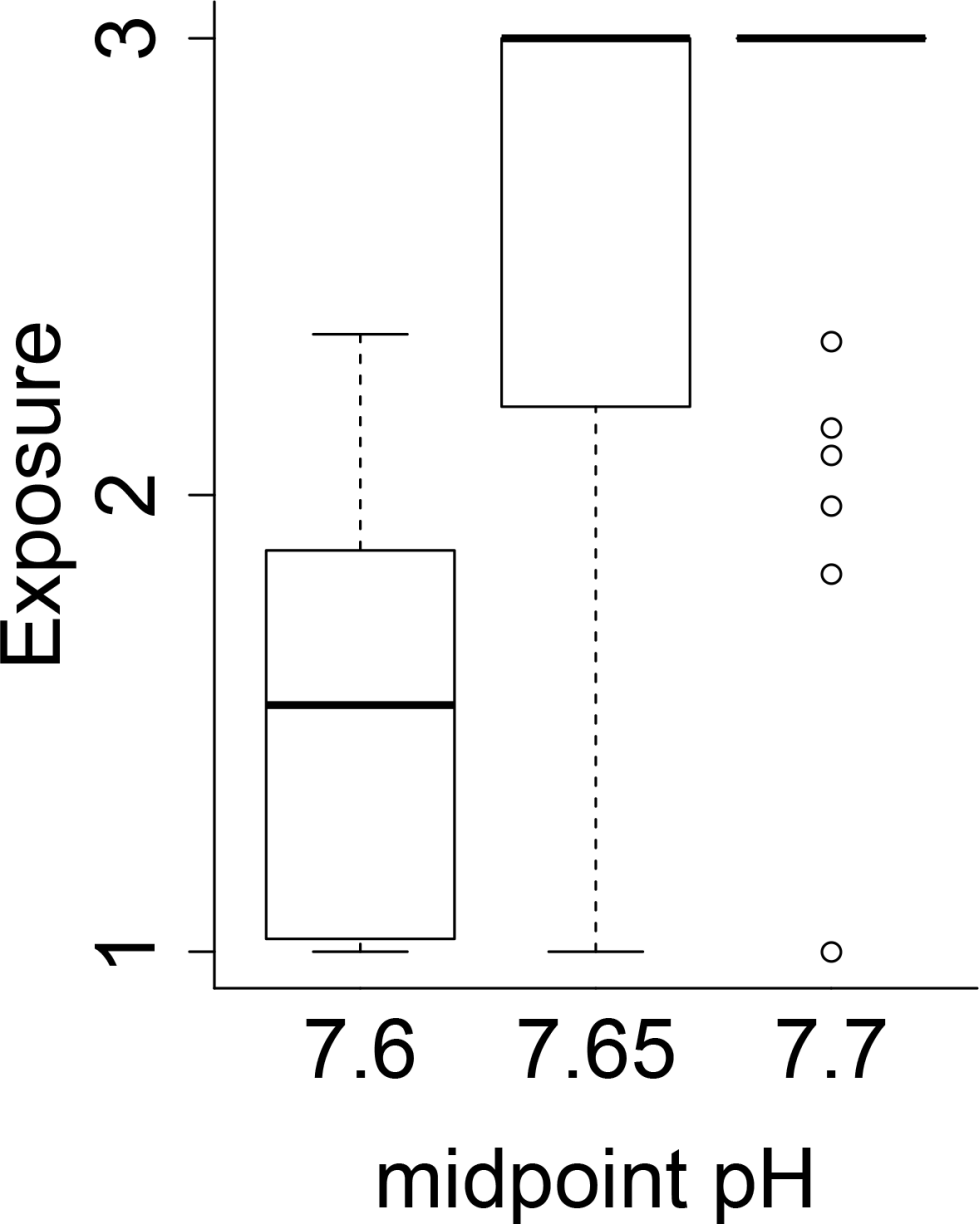
Comparison of exposure scores. Exposure score comparison when using different mid-point pH values across all species and life stages.

Exposure scores were sensitive to the threshold pH value used (Fig 4), producing a wide range of exposure scores for each species’ life history stage (Table 4). Changing threshold pH from 7.6 to 7.65 or 7.7 increased the median exposure from 1.54 to 3 (Fig 4). The high sensitivity to threshold pH levels was due to the fact that the ROMs model predicted many more pH 7.7 conditions than 7.6 conditions i.e. our threshold pH values spanned the edge of predicted future pH values. Because several studies indicate species begin responding within this range, the uncertainty about the precise threshold value produces a wide range of exposure scores across several species life history stages. This indicates that uncertainty in the value of the threshold pH has a large influence on the vulnerability and is a point of notable uncertainty.

**Table 4:** Species specific values for consequence, exposure, life stage weights and the two uncertainty values.

Species	Life Stage	Consequence	Exposure pH = 7.6	Exposure pH = 7.65	Exposure pH = 7.7	Life Stage Weight*	Uncertainty Exposure	Uncertainty Consequence
Dungness crab, *Metacarcinus magister*	Eggs	*1*	*1*.*67*	*3*.*00*	*3*.*00*	*0*.*16*	*1*	*3*
	Larvae	*3*	*1*.*03*	*1*.*43*	*2*.*09*	*0*.*03*	*3*	*2*
	Megalops	*3*	*1*.*00*	*1*.*39*	*1*.*98*	*0*.*03*	*3*	*3*
	Juveniles 1	*1*	*3*.*00*	*3*.*00*	*3*.*00*	*0*.*17*	*1*	*3*
	Juveniles 2	*1*	*3*.*00*	*3*.*00*	*3*.*00*	*0*.*21*	*1*	*3*
	Adult	*1*	*3*.*00*	*3*.*00*	*3*.*00*	***0*.*44***	*1*	*1*
Pink shrimp, *Pandalus jordani*	Eggs	*1*	*1*.*90*	*3*.*00*	*3*.*00*	*0*.*14*	*1*	*2*
	Larvae	*2*	*1*.*04*	*2*.*61*	*3*.*00*	*0*.*04*	*3*	*2*
	Juveniles	*1*	*1*.*89*	*3*.*00*	*3*.*00*	*0*.*27*	*1*	*3*
	Adult	*1*	*1*.*89*	*3*.*00*	*3*.*00*	***0*.*55***	*1*	*2*
Pteropod, *Limacina helicina*	Eggs & larvae	*3*	*1*.*04*	*2*.*24*	*3*.*00*	*0*.*25*	*3*	*2*
	Juvenile	*3*	*1*.*03*	*2*.*42*	*3*.*00*	*0*.*25*	*3*	*1*
	Subadult	*3*	*1*.*04*	*2*.*56*	*3*.*00*	*0*.*25*	*3*	*1*
	Adult	*2*	*1*.*04*	*2*.*42*	*3*.*00*	*0*.*25*	*3*	*2*
Pacific hake, *Merluccius productus*	Eggs	*1*	*1*.*01*	*3*.*00*	*3*.*00*	*0*.*00*	*1*	*3*
	Larvae	*2*	*1*.*06*	*2*.*15*	*3*.*00*	*0*.*01*	*1*	*3*
	Adult	*1*	*1*.*00*	*1*.*00*	*1*.*00*	***0*.*99***	*1*	*1*
Krill, *Euphausia pacifica* Early-spawners	Eggs	*1*	*1*.*67*	*3*.*00*	*3*.*00*	*0*.*03*	*2*	*2*
	Larvae	*2*	*1*.*02*	*1*.*37*	*1*.*83*	*0*.*27*	*2*	*2*
	Juveniles	*2*	*1*.*99*	*3*.*00*	*3*.*00*	*0*.*30*	*2*	*3*
	Sub-adults	*2*	*2*.*35*	*3*.*00*	*3*.*00*	***0*.*32***	*2*	*3*
	Adult	*2*	*2*.*13*	*3*.*00*	*3*.*00*	*0*.*09*	*2*	*3*
Krill, *Thysanoessa spinifera*	Eggs	*1*	*1*.*71*	*3*.*00*	*3*.*00*	*0*.*028*	*2*	*3*
	Larvae	*2*	*1*.*02*	*1*.*52*	*2*.*15*	*0*.*27*	*2*	*3*
	Juvenile	*2*	*1*.*74*	*3*.*00*	*3*.*00*	***0*.*35***	*2*	*3*
	Adult	*2*	*1*.*74*	*3*.*00*	*3*.*00*	***0*.*35***	*2*	*3*

*Bolded values in life stage weights column indicates the stage with the maximum importance for the species.

**Fig 5:**
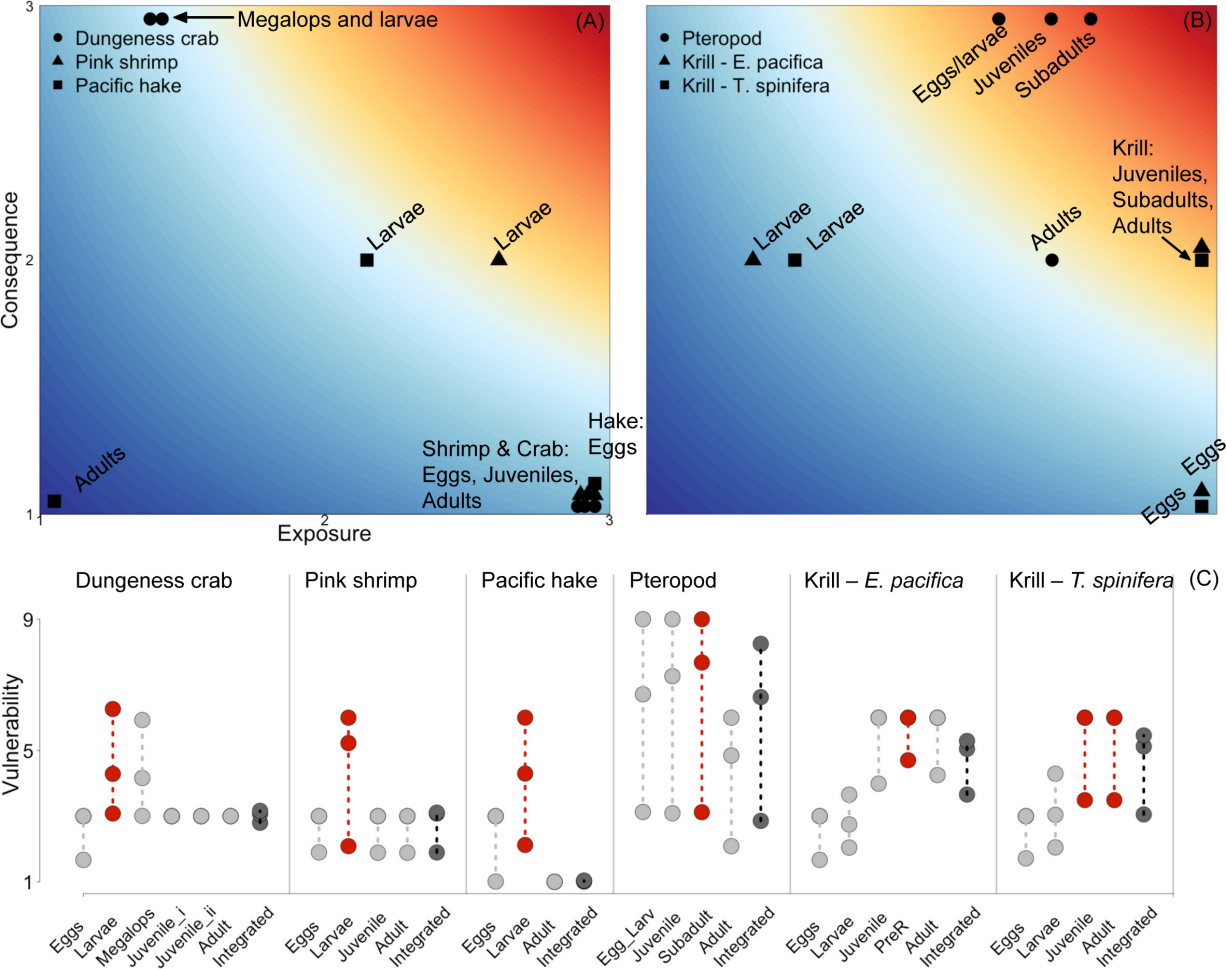
Vulnerability plots for the six species included in our assessment. (A) Exposure and consequence scores calculated with critical pH 7.65 for: crab, shrimp and hake. (B) Exposure and consequence scores calculated with critical pH 7.65 for the three planktonic species. (C) Total vulnerability across all life stages with red indicating the single life stage with maximum vulnerability and dark grey indicating integrated vulnerability. Circles and connecting lines represent the range in vulnerability estimates across the three threshold pH values 7.6, 7.65 and 7.7.

Vulnerability estimates varied considerably across life history stages, due in equal parts to differences in exposure and consequence (Fig 5A and 5B). To illustrate this variability across life history, we present results using threshold pH 7.65 (Fig 5). At the threshold 7.65, differences in vulnerability estimates across life stages of individual species ranged from 1.29 for Dungeness crab to 4.68 for the pteropods, with a mean difference of 2.96 across all species. Exposure and consequences scores within a life stage were often dissimilar, which produced a range of vulnerability estimates across life stages and species (Fig 5). In other words, many life stages had a moderate to low vulnerability estimate resulting from either high exposure paired with low consequence or the reverse (for example, Dungeness crab and pink shrimp adults and eggs, Fig 5A). In contrast, high scores for both exposure and consequence were not common for most species’ stages—the pteropod species was the only species for which this occurred (e.g., the highest vulnerability estimates were for pteropod eggs and larvae, juveniles and subadults, Fig 5B). Pacific hake adults were the only stage to have both low exposure and low consequence, as a result of our assumption that adults are unaffected by low pH.

The relative importance of specific life stages differed across species, and these were reflected in distinct life stage weights (summed elasticity, Table 4). Adult stages were most important for pink shrimp, Dungeness crab, and Pacific hake (leading to weights 0.68–0.99, Table 4). These species are among the most long-lived of the species assessed, and each have relatively long-duration adult stages. Both krill species showed a more even distribution of weights across life stages from larvae through to adults, with slightly higher weights on the juvenile and sub-adult stages (adults: 0.09–0.35, juveniles and subadults: 0.3–0.35, Table 4). Krill are shorter lived, with a short adult duration and relatively longer larval, juvenile and subadult durations.

The stage with maximum vulnerability was not consistent across species (Fig 5C); ranging in value from 4.29–6.00 (with a mean of 5.64) using the pH threshold of 7.65. We had expected that larvae would be the most vulnerable stage for all species, because larvae tend to be more sensitive to low pH [22,23]; however, this expectation was not borne out. For some species, combinations of higher consequence with moderate exposure at the larval stage made this stage the most vulnerable (Dungeness crab, pink shrimp and Pacific hake). For others, consequence scores for the later life stages were as high or higher than early life stages and coupled with higher exposure rates, made the later life stages most vulnerable (pteropods and both krill species). Consequently, it is not possible to derive a generalization about which stage tends to be the most vulnerable.

Integrated vulnerability metrics varied greatly across species and were lower on average than the maximum stage vulnerability. Using the pH threshold of 7.65, integrated vulnerability ranged substantially: between 1.02 and 6.27 (with a mean of 4.18). Pacific hake had very low vulnerability, while vulnerability was moderately-low for Dungeness crab and pink shrimp, moderately high for both krill species and high for pteropods (Fig 5).

Comparing the two metrics, integrated and maximum stage vulnerability were quite similar for some species, while for others they varied substantially. The integrated metric was far lower than the maximum stage metric for three species (Dungeness crab, pin kshrimp and Pacific hake); in hake the difference was almost six-fold. For all three species, this difference was caused by low stage weights for the stage with the maximum vulnerability (larvae), so that high vulnerability in larvae was down-weighted in comparison to the lower vulnerability for later life stages. In contrast, for pteropods and krill, the two metrics were similar. For pteropods this similarity was due to similar vulnerability at all life stages, combined with equal weighting of stages. For the two krill species, the stages that were more vulnerable also tended to have higher life history weights. Thus, for half the species, when the high vulnerability stage was coupled with low stage weight, we found the choice of method profoundly governed the vulnerability estimate.

Considerable uncertainty was introduced by using a range in pH thresholds (Fig 5). Both the maximum stage and integrated metrics showed substantial ranges in value resulting from the pH thresholds of 7.6, 7.65 and 7.7, with the maximum stage metric having larger ranges for all species other than the krill *E*. *pacifica* (mean range of 3.14 for the maximum stage metric and 1.87 for the integrated). At the high end, pteropods had over a 5-point difference in vulnerability across pH thresholds, for both metrics. Pteropod vulnerability therefore ranged between moderate-low and high, depending on the threshold value used. In almost all cases, different vulnerability estimates would be reached depending on the threshold chosen.

**Fig 6:**
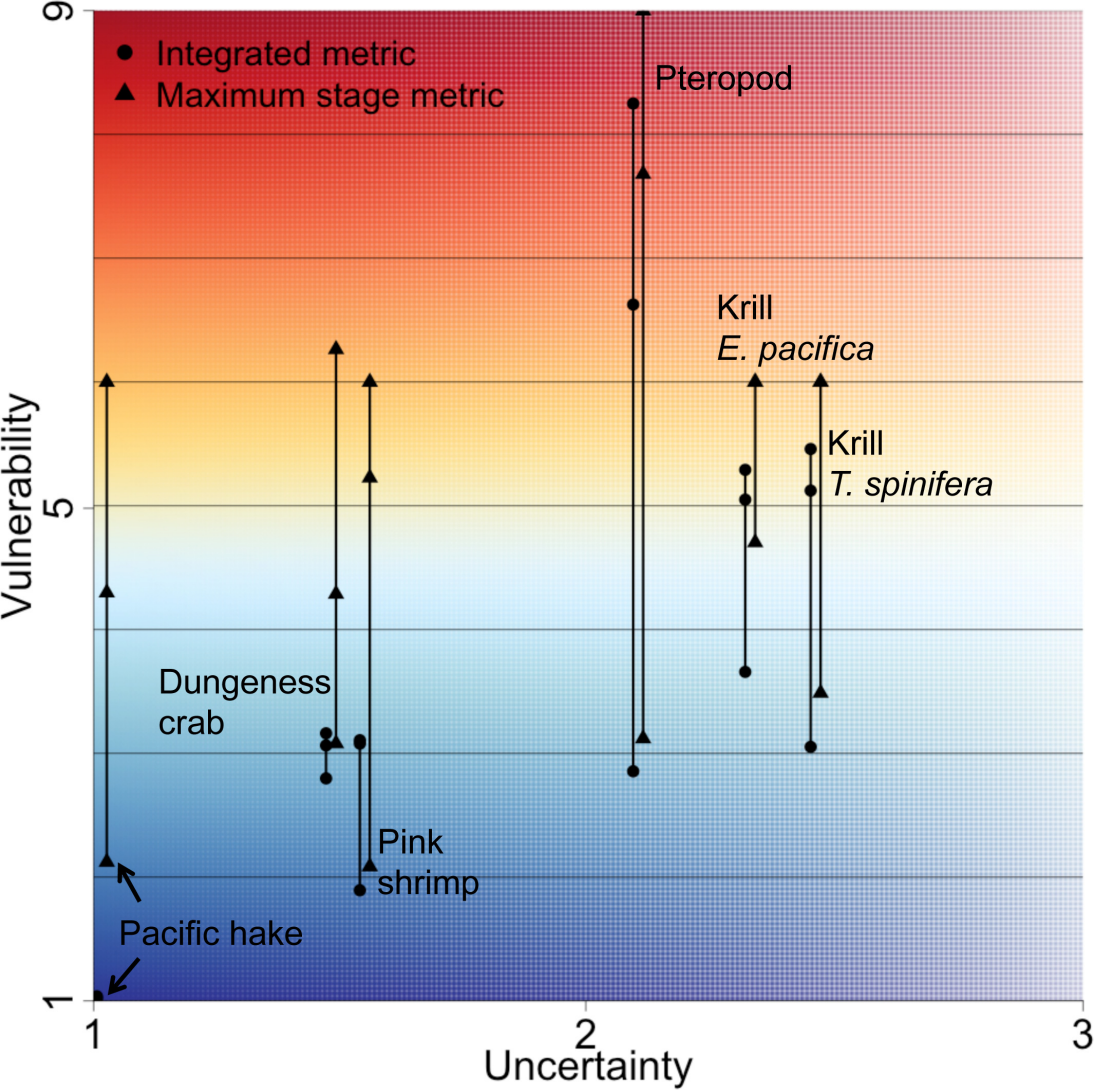
Vulnerability and uncertainty combined. Vulnerability shown as both integrated (circles) and maximum stage (triangles) plotted against uncertainty. Species vulnerabilities range due to critical pH values between 7.6, 7.65 and 7.7.

All species had high uncertainty in either exposure or consequence but not both, leading to population uncertainties ranging from 1–3 (3 would be the maximum, if uncertainty in exposure and uncertainty in consequence were both 3; Table 4, Fig 6). Uncertainty in consequence was consistently high, as there was limited information on the response of species’ life stages to OA; pteropods were the exception with low uncertainty in consequence, but high uncertainty in the other areas. By definition, uncertainty was closely related to the amount of previous research across a species’ life stages; hence uncertainty in exposure, which was related to knowledge about life stage distributions, was low for most stages of Pacific hake, Dungeness crab and pink shrimp. These fishery species have known distributions, except for the larval stage of Dungeness crab and pink shrimp.

In some cases, the large source of uncertainty introduced from the three thresholds (range of y values in Fig 6) exceeds the uncertainty calculated directly for the exposure and consequence components (Fig 6). This was particularly noticeable for Dungeness crab, pink shrimp and Pacific hake, which had population uncertainty at the lower range (less than 2, Fig 6), but with large ranges in vulnerability resulting from different thresholds. In this case, the maximum stage metric ranged by over 3 points, greatly influencing the vulnerability conclusion. This was found because the thresholds used were on the lower end of pH values predicted in the ROMS model [31], with many more values of pH predicted near 7.7 than there are at 7.6.

## Discussion

Many anthropogenic stressors cover large spatial areas [14, 15] and may interact with species at multiple points in their life cycle (e.g., pollution, temperature changes, hypoxic events), requiring that we find a way to assess organism vulnerability as it changes through life. The notion that response to environmental stressors changes through life is not new [22, 59, 60], yet until now its relevance to applications of semi-quantitative vulnerability assessment had not been demonstrated. We propose a simple modification to a rich history of vulnerability assessment methods [9, 12, 13] that involves explicit consideration of exposure and consequence with life stage. This method resulted in clear changes through life in vulnerability to ocean acidification for all species in this assessment. Those stages with the highest consequence often had lower exposure, and vice versa, demonstrating the need to look at both concurrently. For example Pacific hake, Dungeness crab and pink shrimp larvae had high consequence with lower exposure, indicating that the distributions of sensitive planktonic larvae may naturally avoid the most harmful conditions, which for OA tend to be in the upper water column in offshore regions.

Translating life stage vulnerabilities into a single population level vulnerability estimate was sensitive to the method used. For species where the life stage that is most vulnerable also has the highest relative weight, the two metrics we used would be similar and either method could be employed (however, this was not found for most species in our assessment). In contrast, when the most vulnerable stage has a low relative weight, the single-stage and integrated metrics can vary substantially.

Using the maximum stage metric leads to a clear identification of which stage is most susceptible to a stressor, making it useful in certain circumstances. If the goal of the vulnerability assessment is to focus on an individual species to determine the most vulnerable stage or areas of high uncertainty, then this approach is appropriate. However, if the goal is to identify the most vulnerable species within a subset of species of interest, a common goal in these assessments [5], then this approach may lead to false positives. Singling out the most vulnerable stage without factoring in the relative importance of that stage may lead to a high vulnerability estimate for a species that in fact will be minimally impacted. When considering a range of species, this may result in low priority species falsely being assigned as high priority, downgrading those that are in fact more vulnerable.

On the other hand, though it contains more information about all stages of a species, the integrated approach may lead to false negatives. In circumstances where the impact on the low weight stage is truly catastrophic, the impact on the population may very well be severe but this metric would negate the effect. If 100% mortality were imposed on a stage with low elasticity, we would not want to ignore this highly vulnerable stage. For this reason, both approaches are useful as together they better frame the range of vulnerability estimates for a species. We can return to our earlier point: when the two metrics agree, the assessment is clear. In cases where the two do not agree a more complicated model, for example including density dependence, may be needed to gain insight into the true impact of exposure and consequence for the population.

Our assessment highlighted the value of a detailed, stage-specific approach to judge vulnerability as functions of both exposure and vulnerability. For example, three species had considerable fisheries value: Pacific hake, pink shrimp and Dungeness crab. Of those three, none were found to have high vulnerabilities (1–6, depending on which metric was used), and each had low levels of uncertainty (Fig 6). Some of these species might have been judged to be vulnerable to OA if only consequence was considered. For example, a recent study [61] revealed high consequence of Dungeness crab larval exposure to pH 7.5, yet our study suggests that larval crab exposure to such low pH is relatively rare, and therefore the larval stage vulnerability score was low-moderate.

In comparison, the three holoplankton species in our assessment, pteropods and two krill, were found to have moderate to high vulnerability and uncertainty estimates on the higher end. The high uncertainty of the vulnerability estimate highlights the value of increasing our understanding of these species’ vulnerability to ocean acidification. High uncertainty may mean that these species have true vulnerability levels that are higher or lower than those estimated. Krill are a critical component of marine food webs, and are a very important prey resource in the California Current [62], so additional knowledge of the distributions of early life stages and responses to pH will greatly enhance our assessment of krill vulnerability and therefore our understanding of the potential for broader ecological effects propagated through the food web.

Our assessment factored in uncertainty to help determine our confidence in the vulnerability estimates, but we found uncertainty to be unexpectedly large from the three pH thresholds used. Uncertainty in exposure and consequence can be used to help identify species for which we have limited biological knowledge. However, ranges in vulnerability estimates from the pH thresholds were frequently large, sometimes exceeding uncertainty estimates from exposure and consequence (Fig 6). The threshold pH is the value at which negative consequences of pH are believed to occur. We found that if the true threshold was 7.6, all species would have vulnerability below 5 (out of a maximum of 9) and be in the lower range of vulnerability estimates. In this case, the maximum stage and the integrated vulnerability metrics would be quite similar across the species, resulting in less challenge when deciding which metric is most appropriate. In contrast, if the threshold was 7.7, then we found very different vulnerabilities between metrics for some species, and higher overall vulnerability estimates. Clear identification of the appropriate pH threshold for each species would provide substantial insight into the true vulnerabilities of these species from ocean acidification. Additionally, true species vulnerability depends strongly on model predicted pH values and model uncertainty can be considerable and is best addressed by multi-model comparison [63].

Because we used the oceanographic predictions from a previously published model [31], our exposure mapping is based on monthly average pH levels and does not capture shorter duration pH levels that may impair species [41, 64]. Research has shown that pH can range by 0.499 off Monterey Bay, California and by 0.397 off Point Conception, California within a 30-day period with pH ranging up to 0.35 within the span of days [41]. Many organisms that already reside in these high fluctuating environments are likely adapted to cope with some level of fluctuation, but increased frequency of long-term extremes may cause harm [41, 64, 65]. Additionally, in the California Current, species are already experiencing pH around 7.6 [34], which is the predicted mean pH for global oceans in 2100 [66]. Thus our threshold pH of 7.6–7.7 is within the range of what some species currently experience.

There are three key assumptions regarding species characteristics that warrant consideration. First, distributions were assumed to remain constant over the next 35 years. Given the minimal information available to map current distributions of all life stages, too many additional assumptions would be required to predict future distributions. Whether OA will cause distributional shifts is unknown. However, there is evidence that such shifts have already occurred in response to temperature [67, 68], suggesting that distributions will change but with a large unknown about the role that OA will play. Second, we did not account for the possibility of avoidance behavior. Avoidance behavior is even less well documented; however there is considerable evidence of species shifting distributions to avoid hypoxic conditions, including avoidance by larvae (e.g., [69–72]). Finally, in our assessment species were not assumed to have the capacity to evolve an adapted response to low pH [73]. Further refinement of the approach could involve addressing one of these assumptions.

In addition to threats from ocean acidification, these marine species will be experiencing warming oceans, hypoxia, point source pollution, and other stressors, all highlighting the importance of scaling up from single stressor studies to understanding cumulative impacts [15, 74]. Tight associations between variables need to be considered as they have been shown between naturally occurring temperature and pH values [64], and between temperature with pCO_2_ and with aragonite saturation [75]. Although not within the scope of this work, the relative impact of stressors needs to be accounted for, as some are more influential than others. For example, temperature can have a stronger influence than OA [76]. While in this study we advance methods in vulnerability assessment incorporating life stage considerations, there are important steps that remain to be taken towards cumulative impacts assessment.

Understanding and predicting species responses to environmental stressors is one of the fundamental questions in modern ecology [3, 77]. Given the considerable resources required for quantifying population change, less data-dependent methods are valuable tools [78]. Obtaining predictive power requires combining research from numerous approaches: physiological tolerance studies, behavioral studies, direct observation and population modeling [3], but has also been argued to be likely not achievable [79]. Consequently, the extensive level of effort needed to gain insights into possible futures is only warranted in cases where a species has some reasonable risk of exhibiting ecologically significant population-level effects. Vulnerability assessment is a critical point in the risk framework that can help direct management. Here we have introduced an approach that can help identify highly vulnerable stages and populations when the stressor being investigated is one which can influence species at multiple points in their life history.

## Supporting Information

S1 File (PDF)
