# Supplementary material for: Extending Vulnerability Assessment to Include Life Stages Considerations
Source: PLoS One. 2016 Jul 14;11(7):e0158917. doi: 10.1371/journal.pone.0158917 (PMC4945077; doi:10.1371/journal.pone.0158917)
Supplement: S1 File — (PDF) [file pone.0158917.s001.pdf]

Appendices

Appendix A: Details on mapping species distributions

Table A1. Description of distributions for species life stages and associated maps.

Species	Life Stage	Assumptions	Citations
Metacarcinus magister	Eggs	The eggs of Dungeness crab are retained on female crabs and are found off the coasts of Washington and Oregon between October-March and off California between September-February. They are along the bottom.	(Reilly 1983, Pauley et al. 1986, Rasmuson 2013)
	Zoea	Dungeness crab larvae have exhibited movement far offshore, starting over the continental shelf and progressively moving out past the continental shelf before metamorphosing into megalops. As a result, larvae were mapped as close to shore for the first month in the water column and progressively moving farther out. Down to a depth of 70 m.	(Reilly 1983, Hobbs and Botsford 1992, Hobbs et al. 1992, Rasmuson 2013)
	Megalops	Megalopae have also been found off the continental shelf, but move into the nearshore environment to settle. Megalopal distributions were only mapped for the planktonic portion of their life since once they move inshore to settle, they tend to go into estuaries – regions for which we do not have any oceanographic estimates.<br>Their distributions match that of Zoea in the last month of where zoea are found before metamorphosing also at 70 m.	(Reilly 1983, Rasmuson 2013)
	Juveniles i and ii	Research has shown that newly settled juvenile crabs tend to settle in estuaries, however given the relatively few estuaries along the outer coast of the California Current Rasmuson (2013) note that they settle where adults are found.	Rasmuson (2013)
	Adults	Dungeness crab adults are predominantly found between 30-90 m depth; they are only occasionally found in the surfzone. Thus the distribution of adult Dungeness crab has been assumed to be between 30-90 m depth, from the outer coast of Washington to Santa Barbra California. Along the bottom.	Rasmuson (2013)
Pandalus jordani	Eggs	Females retain their eggs and ovigerous females are found off Oregon and Washington in October-March, off Northern California in October-April and Southern California November-June. Eggs are assumed to be where	(Dahlstrom 1970, 1973)

		adults are found along the bottom of the ocean. Found along the bottom.	
	Larvae	The distribution of pink shrimp larvae has been minimally investigated, with most of the research conducted in the 1970s. The estimated distribution of pink shrimp larvae therefore depend on a number of assumptions. Larvae are found within 55 km of shore for the first month present in the water column, and then out to 110 km as they disperse via advection and diffusion. Therefore larval shrimp distribution covers a large area from 2 km offshore out to 110 km from during most months of the year when they are present. Pink shrimp larvae have been found between the neuston and 150 m depth, with the majority above 100 m, thus the depth of 100 m was used in this analysis.	(Rothlisberg and Pearcy 1976, Rothlisberg and Miller 1983)
	Adults and Juveniles	The highest concentration of shrimp adults and juveniles are found between 80-230 m bathymetries, along the bottom of the ocean.	(Hannah 2011)
Merluccius productus	Eggs	The distribution of pacific hake eggs comes from CalCOFI surveys (1984-2012). CalCOFI data were only used for years when greater than 500 samples were collected, and we disregarded tows with fewer than 5 individual hake egg or larvae. Survey data is available for all months except December and thus the distribution of eggs during December was assumed to be the same as January (sensitivity to using January vs. November was tested and had no impact). A convex hull was created in ArcGIS around the presence points.<br>Eggs were assumed to be found down to 150 m	(Moser et al. 1997, CalCOFI 2012)
	Larvae	Maps of hake larvae were developed using the same methods as for hake eggs.<br>Larvae were assumed to be found down to 100 m	(Cass‐Calay 2003, CalCOFI 2012)
Euphausia pacifica	Eggs	Spawning for *E. pacifica* occurs March-October, but most heavily in July and August. Due to the more rapid development time of eggs spawned earlier in the season, there are two sets of durations – March-May eggs for the early spawners, and June-October eggs for late spawners. Eggs are also thought to be found onshore, where adults are offshore – thus were mapped from the coast to the 300 bathymetry line.	(Brinton 1976, Feinberg and Peterson 2003, Feinberg et al. 2010, Harvey et al. 2010, Feinberg et al. 2013) Keister pers. comm.
	Larvae	Larvae have the same spatial distribution as eggs, and a duration of 2	(Lu et al. 2003c, Bi et al.

		months, thus are found March-June for early spawners and June-November for the late spawners	2011)<br>Peterson pers. comm.
	Juveniles	Juveniles are assumed to have the same distribution as adults which is off the shelf. They are assumed to occur from the 200 m isobaths outwards off the shelf, and down to a depth of 300 m.<br>Early spawned juveniles are present May through August and late spawned juveniles are present August through the following April.	(Lu et al. 2003c, Vance et al. 2003)<br>Peterson and Keister pers. comm.
	Sub-adults	Sub-adults have the same distribution as adults, as they are essentially adults, just slightly smaller and less fecund. The early spawned sub-adults are found July through November and late spawned sub-adults are January through the following July.	(Lu et al. 2003a, Vance et al. 2003)<br>Peterson and Keister pers. comm.
	Adults	Adults are found offshore of the shelf-break during all months of the year at a depth of 300 m.	(Lu et al. 2003a, Vance et al. 2003)<br>Peterson and Keister pers. comm.
Thysanoessa spinifera	Eggs	Thysanoessa is thought to be an on-shelf species. Thus all stages of this species are inwards of the 200 m isobath.<br>Spawning occurs February to September.	(Feinberg and Peterson 2003, Lu et al. 2003c, Feinberg et al. 2010)
	Larvae	Larvae are then present February through October developing from eggs (also on shelf)	
	Juveniles	Juveniles and adults are both found all months of the year	
	Adults	Juveniles and adults are both found all months of the year	
Limacina helicina	Eggs/Larv	There is evidence for continuous spawning through the year (Wang 2014) however, Wang found there to be much stronger spawning in the spring through fall with little to none in the winter. From this we assume eggs and larvae are present March-September which is the dominant period of spawning.<br>Depth of 100 m used for all stages from Bednarsek et al. (2014)	(Bednaršek et al. 2014, Wang 2014)<br>Pers. comm. Bednarsek
	Juveniles	Juvenile duration is thought to be approximately three months, thus they are present in the water column after larvae have developed (estimated as 2 months) and are found May-November.	Pers. comm. Bednarsek
	Sub-adults	Sub-adults are also estimated to have a three month duration and are assumed to be found in the water column July-February	Pers. comm. Bednarsek

	Adults	Adults are found year round and with the same spatial distributions of the other life stages	(Wang 2014)<br>Pers. comm. Bednarsek

Appendix A Figs

Dungeness crab, *Metacarcinus magister*, maps

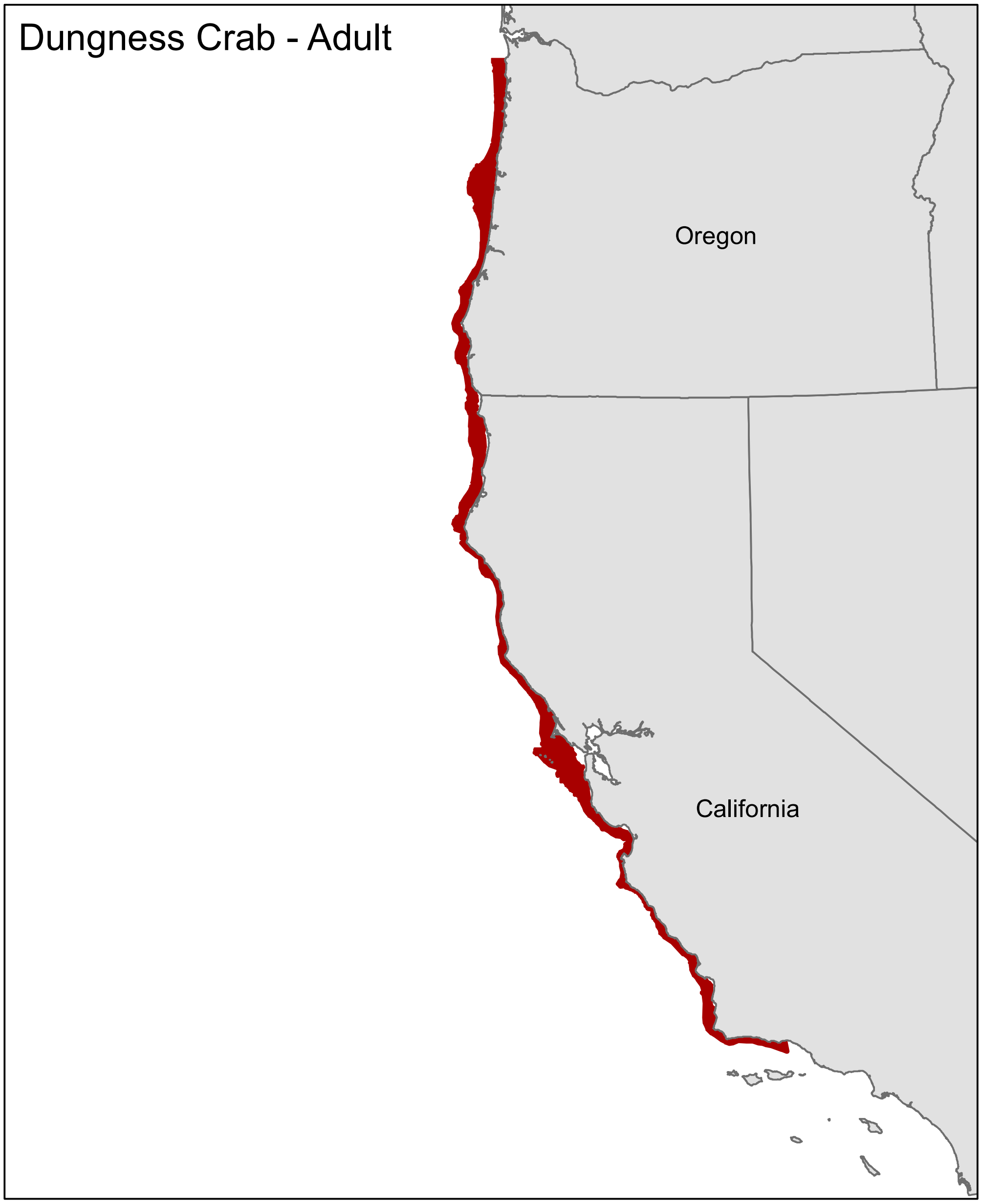

Fig A1. Adult Dungeness crab distributions, January-December

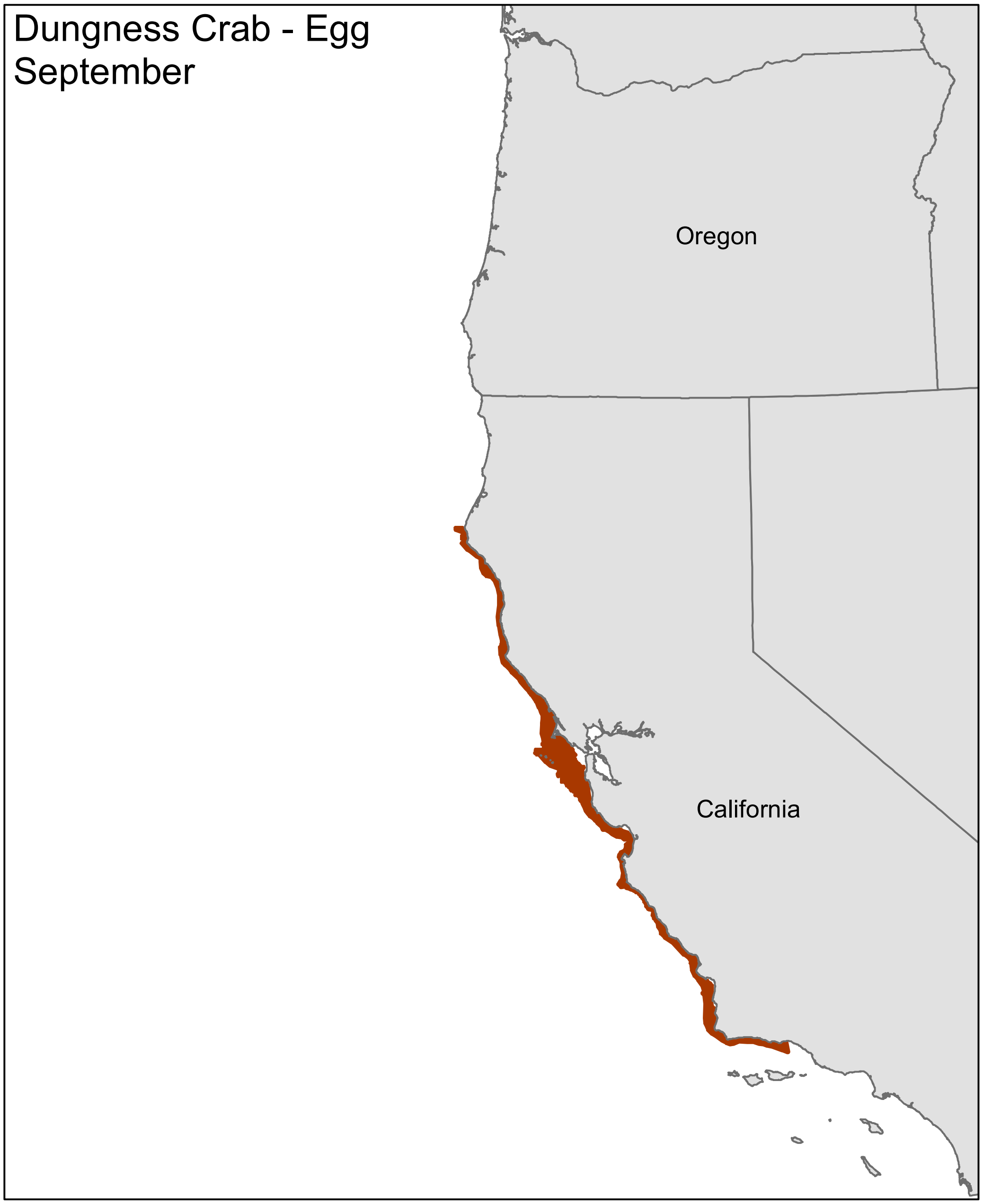

Fig A2. Dungeness crab egg distribution, September.

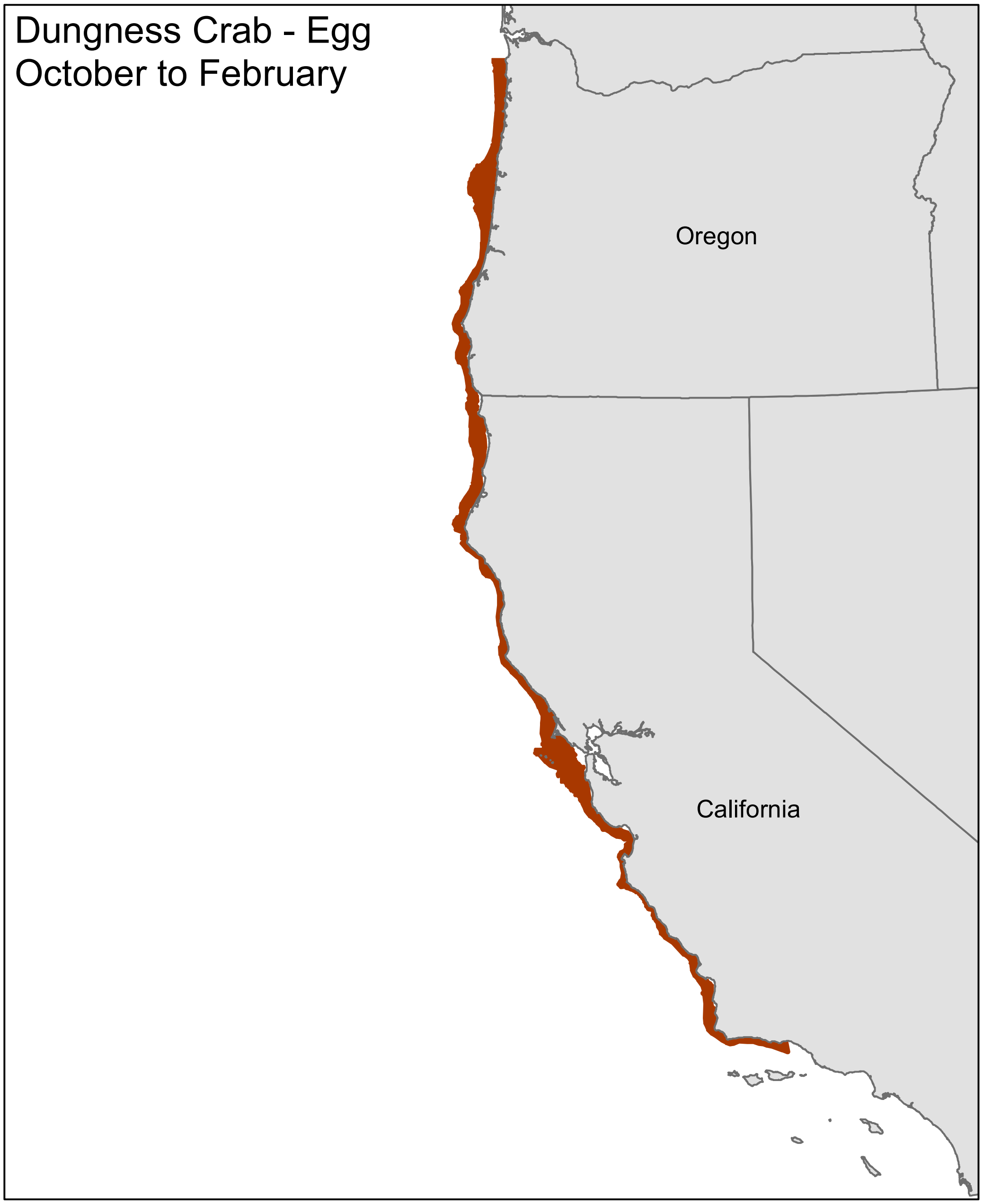

Fig A3. Dungeness crab egg distribution, October to February.

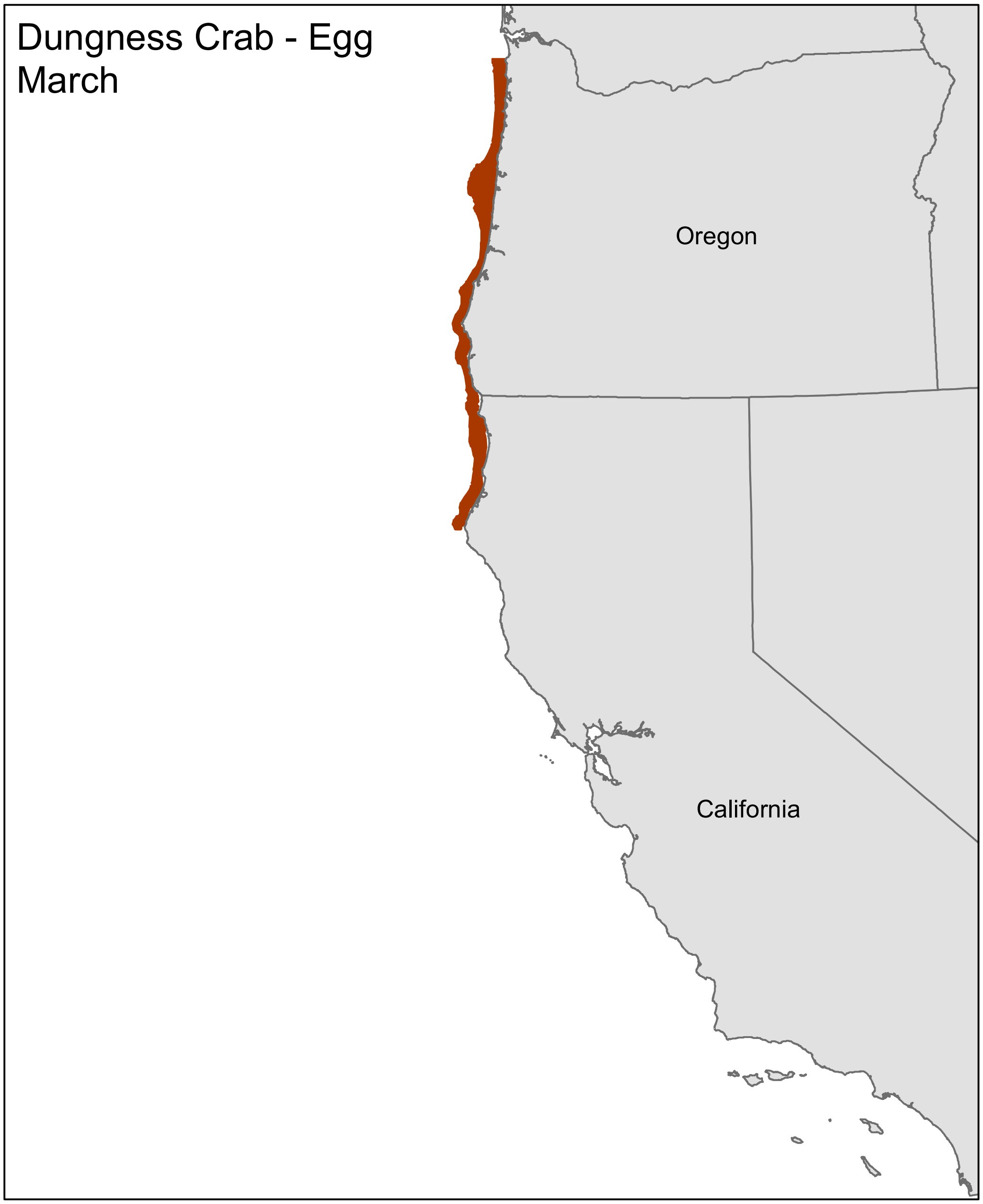

Fig A4. Dungeness crab egg distribution, March.

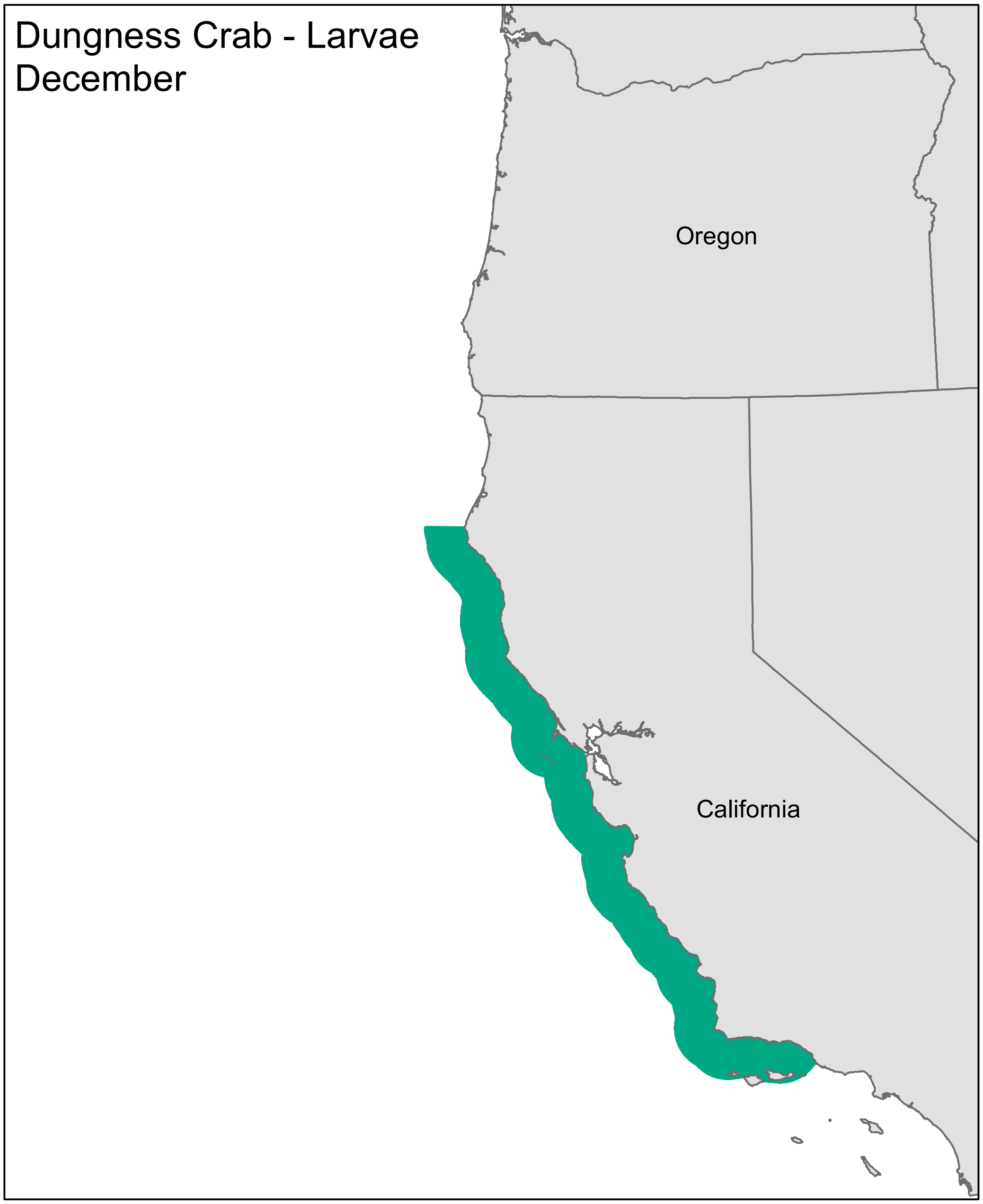

Fig A5. Dungeness crab larval distribution, December.

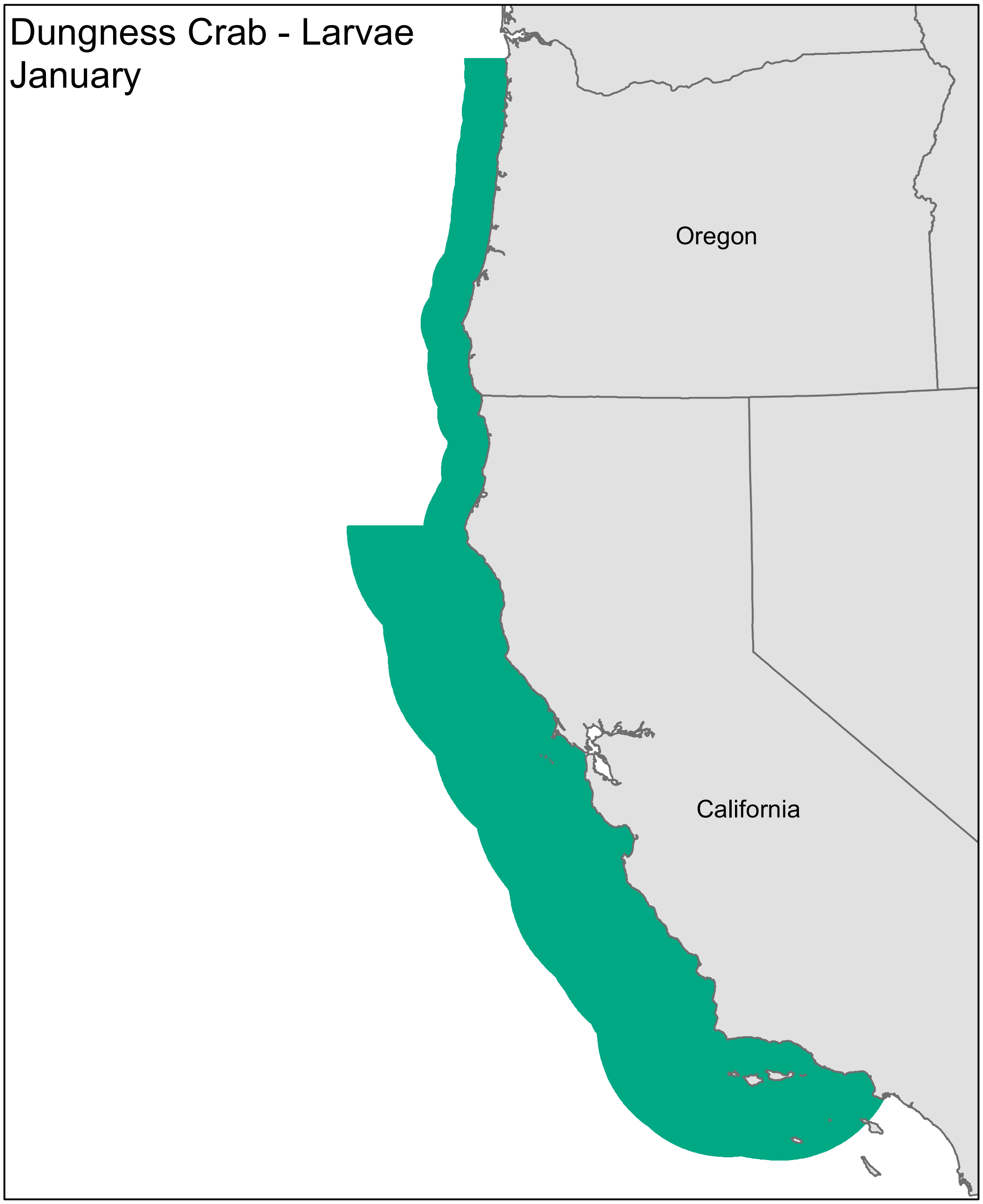

Fig A6. Dungeness crab larval distribution, January.

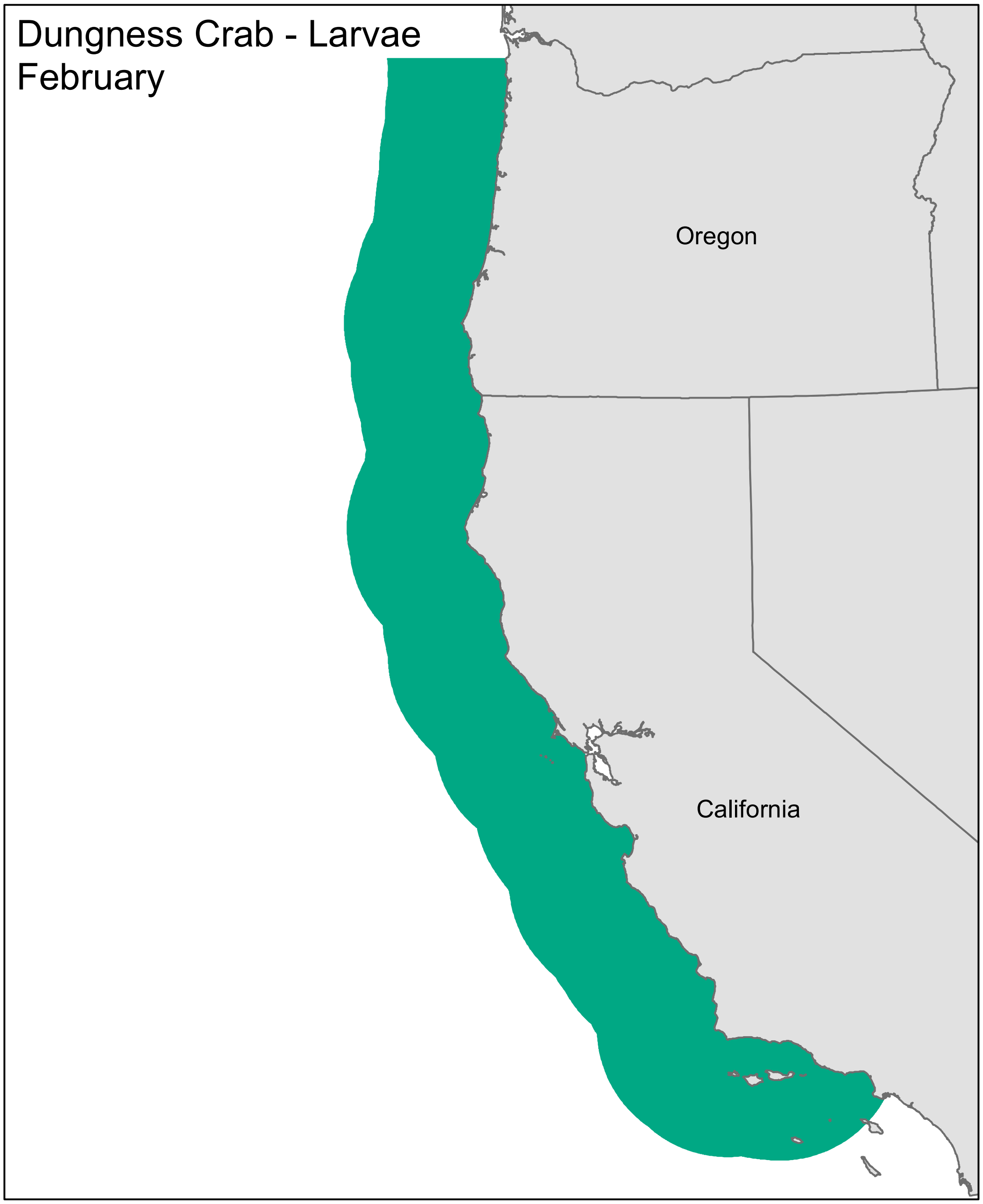

Fig A7. Dungeness crab larval distribution, February.

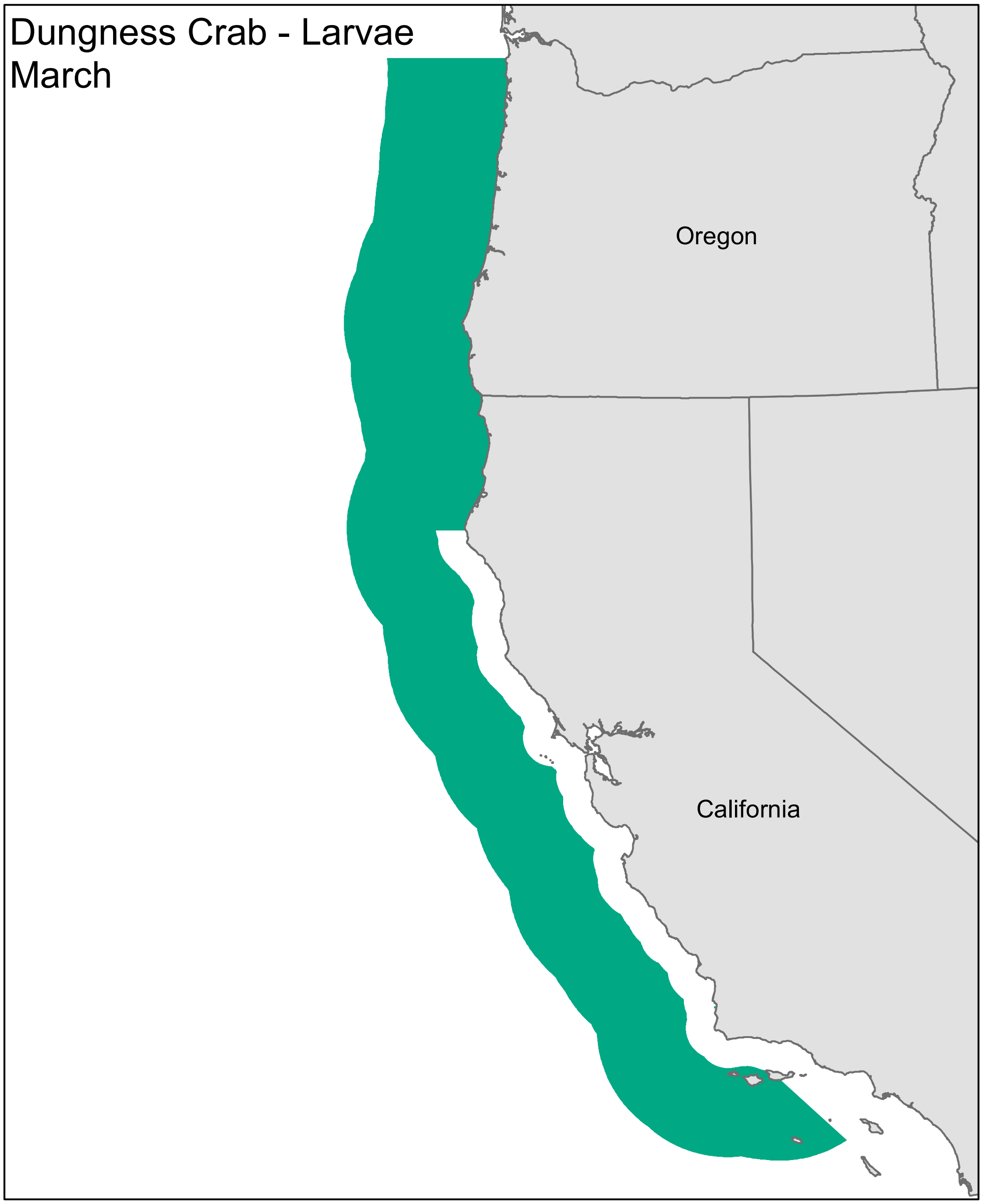

Fig A8. Dungeness crab larval distribution, March.

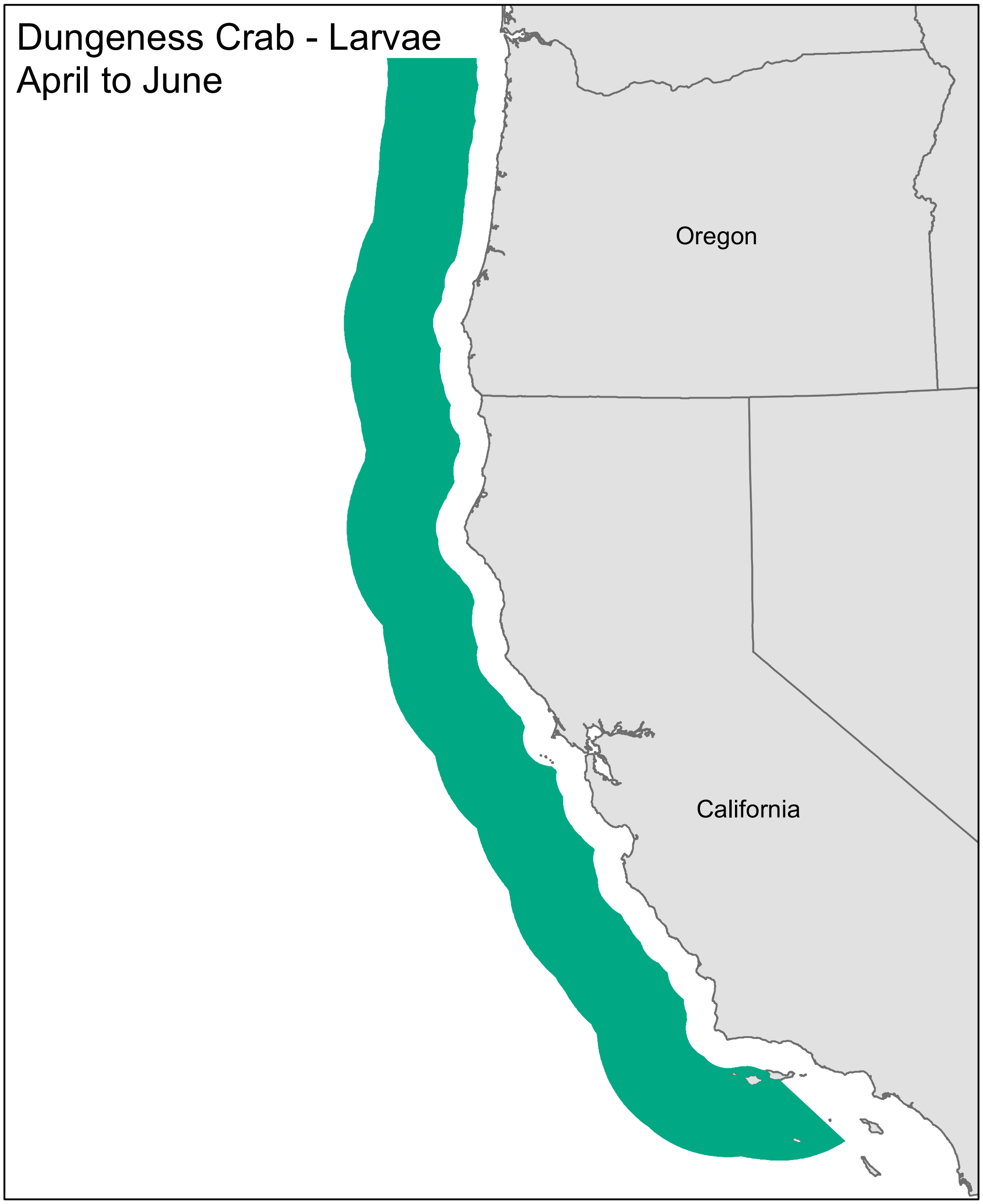

Fig A9. Dungeness crab larval distribution, April through June.

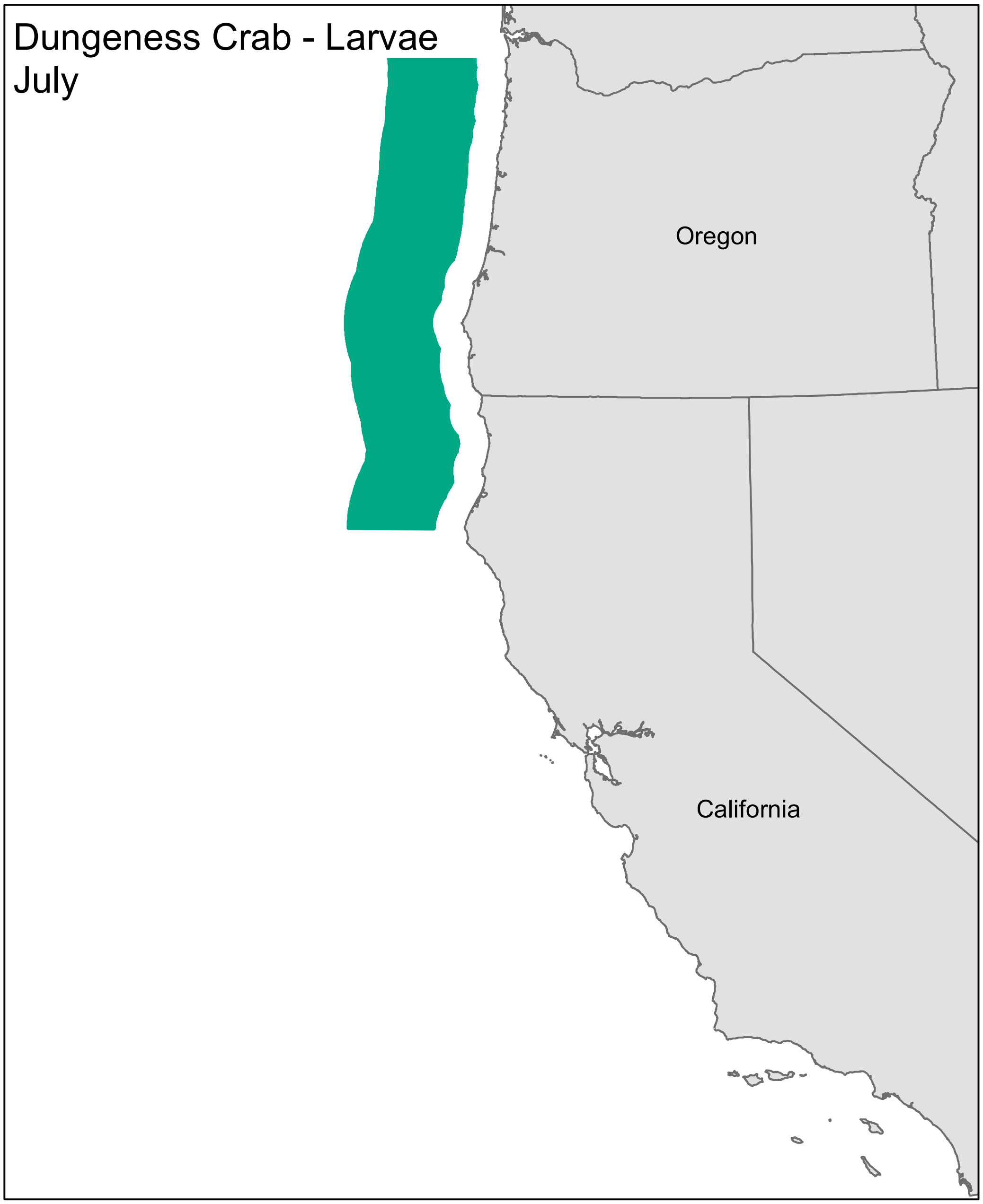

Fig A10. Dungeness crab larval distribution, July.

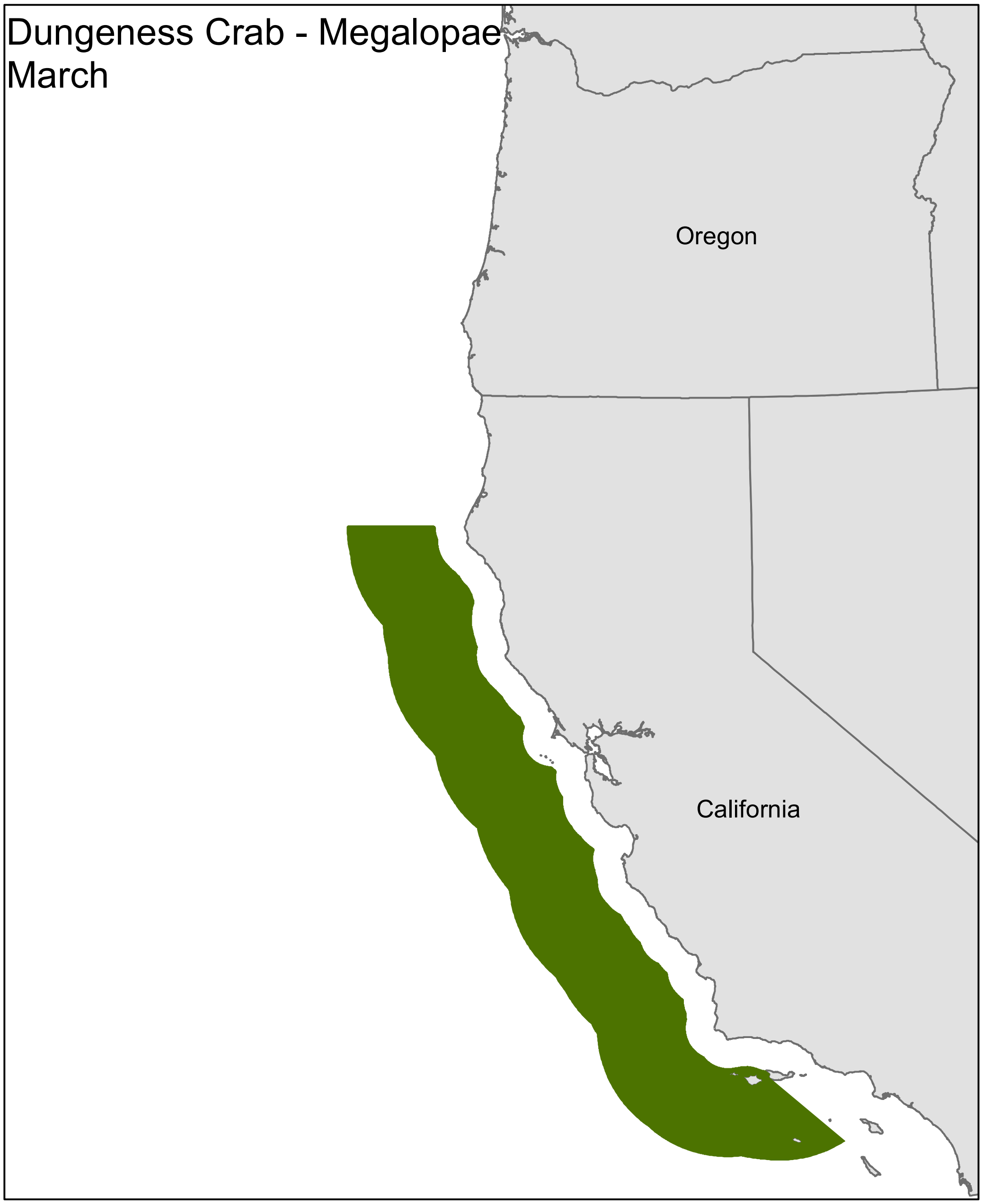

Fig A11. Dungeness crab megalopae, March.

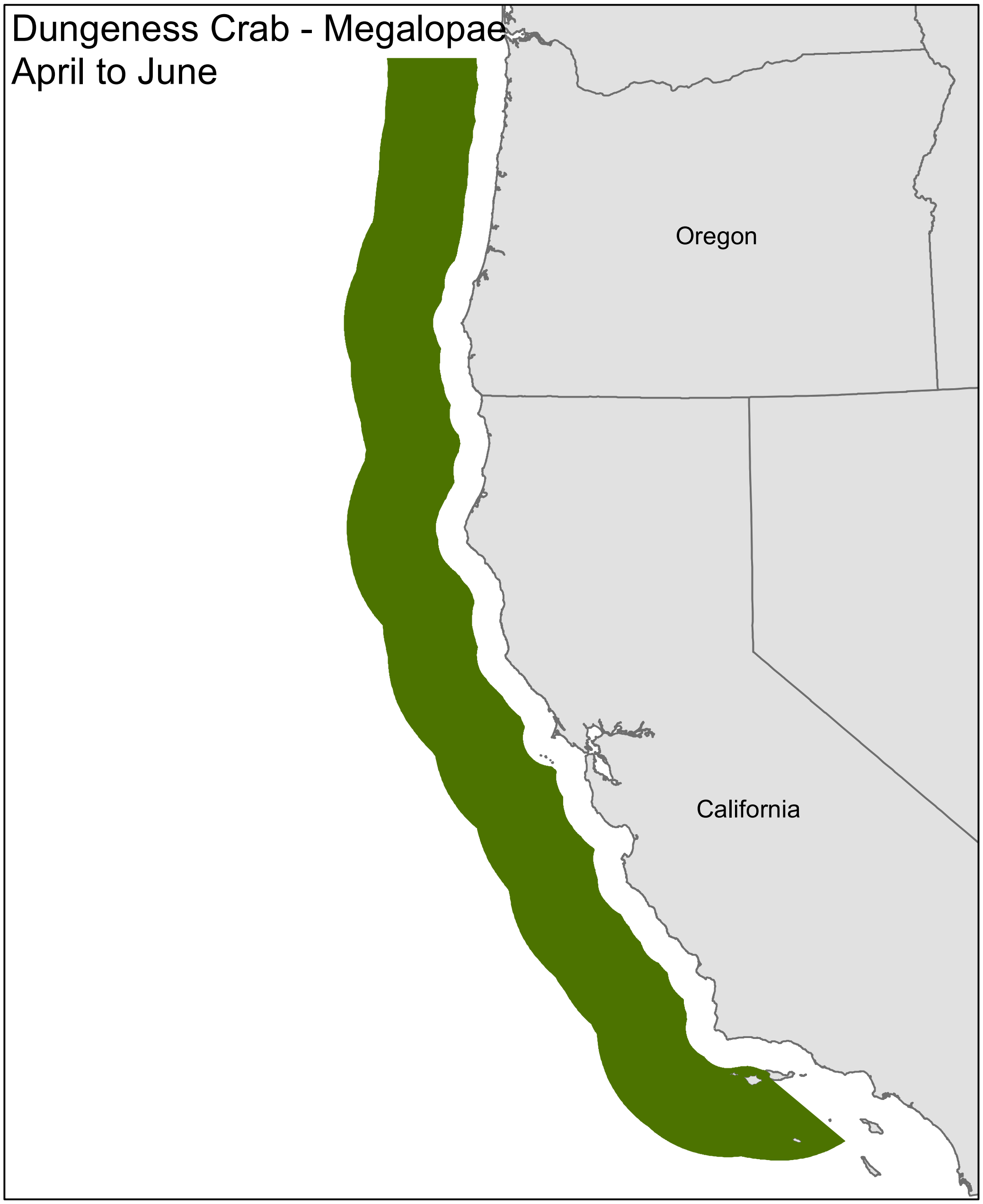

Fig A12. Dungeness crab megalopae, April through June.

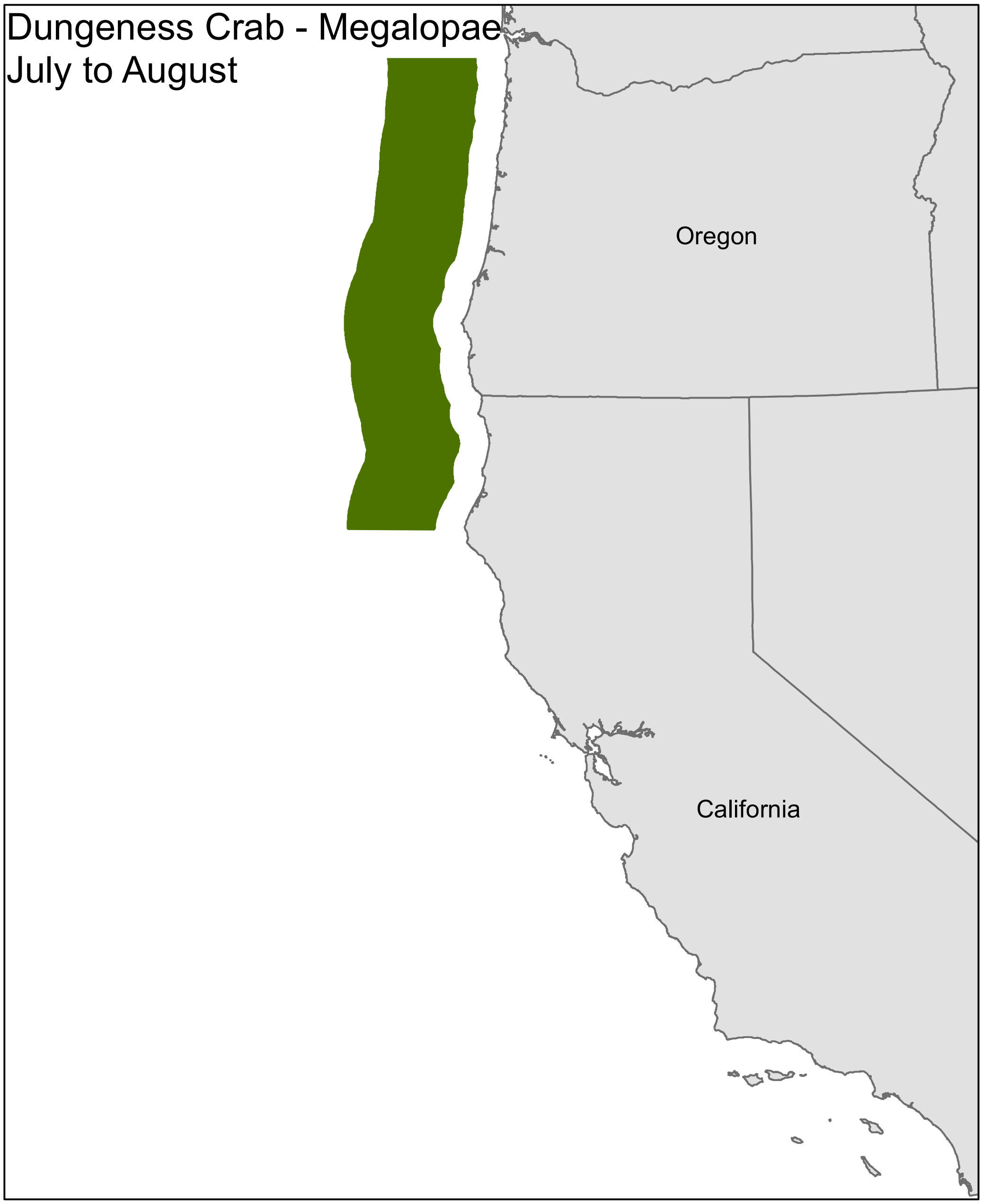

Fig A13. Dungeness crab larvae, July and August.

Pink shrimp, *Pandalus jordani*, maps

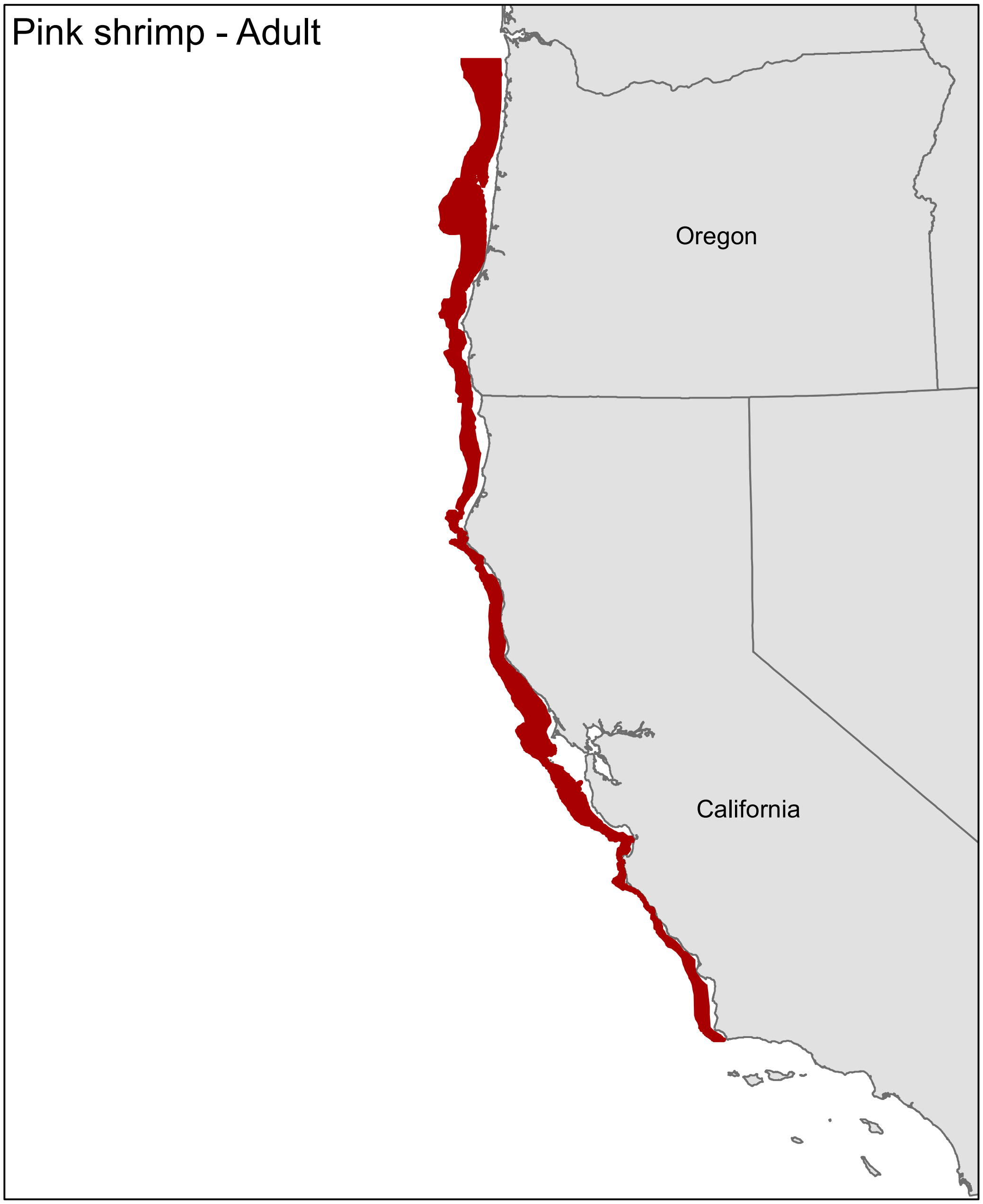

Fig A14. Pink shrimp adult distribution, all months.

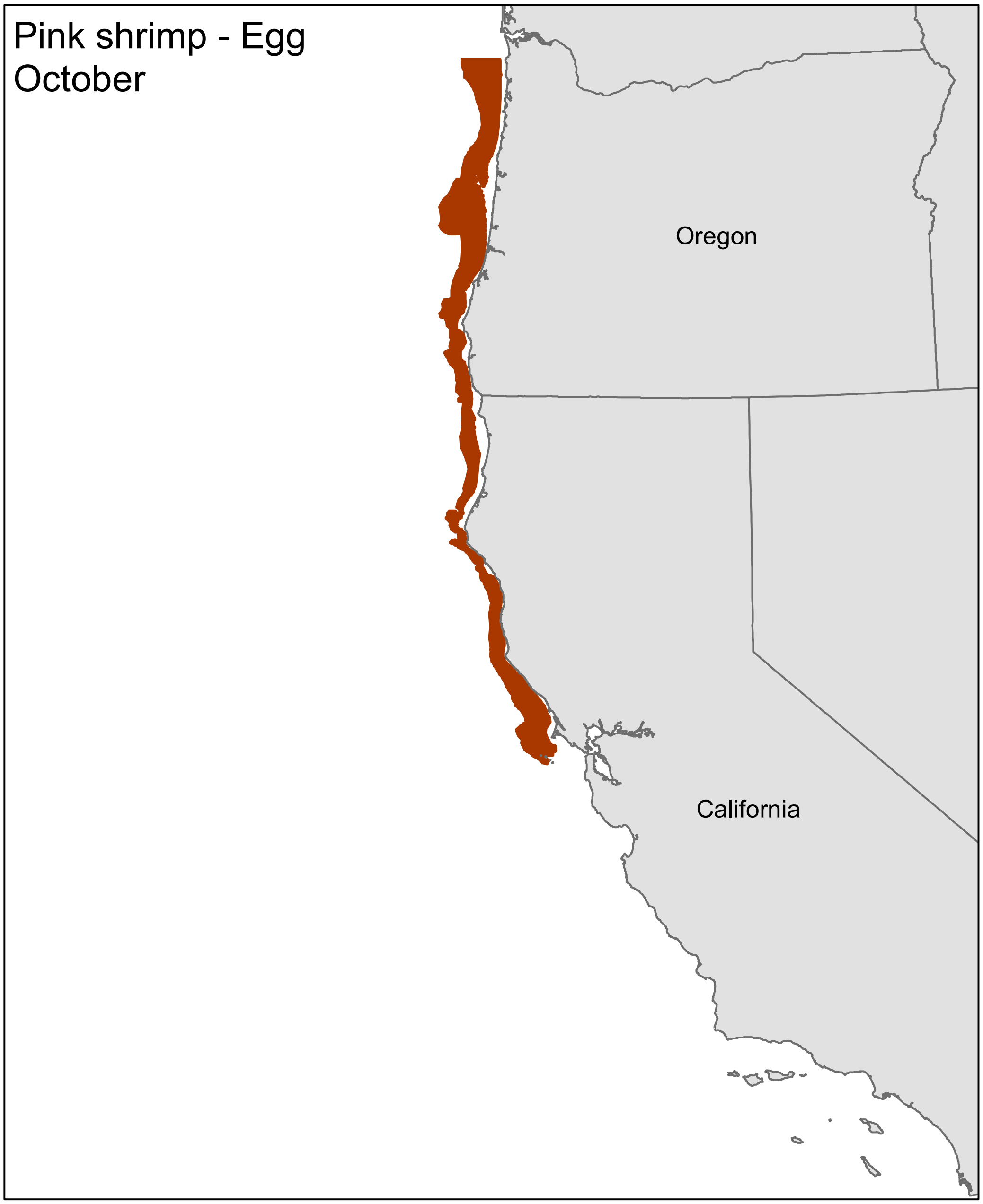

Fig A15. Pink shrimp egg distribution, October.

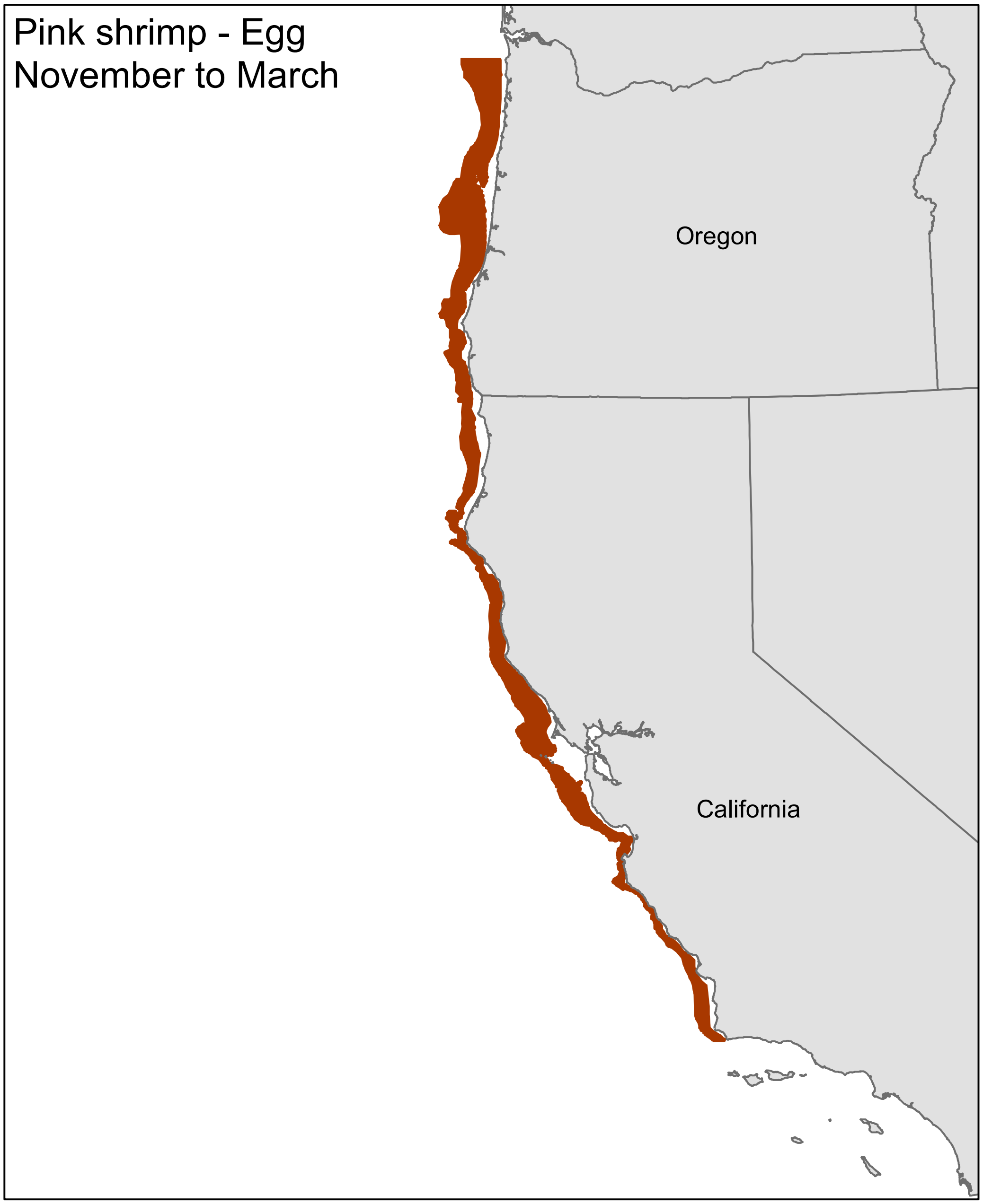

Fig A16. Pink shrimp egg distribution, November through March.

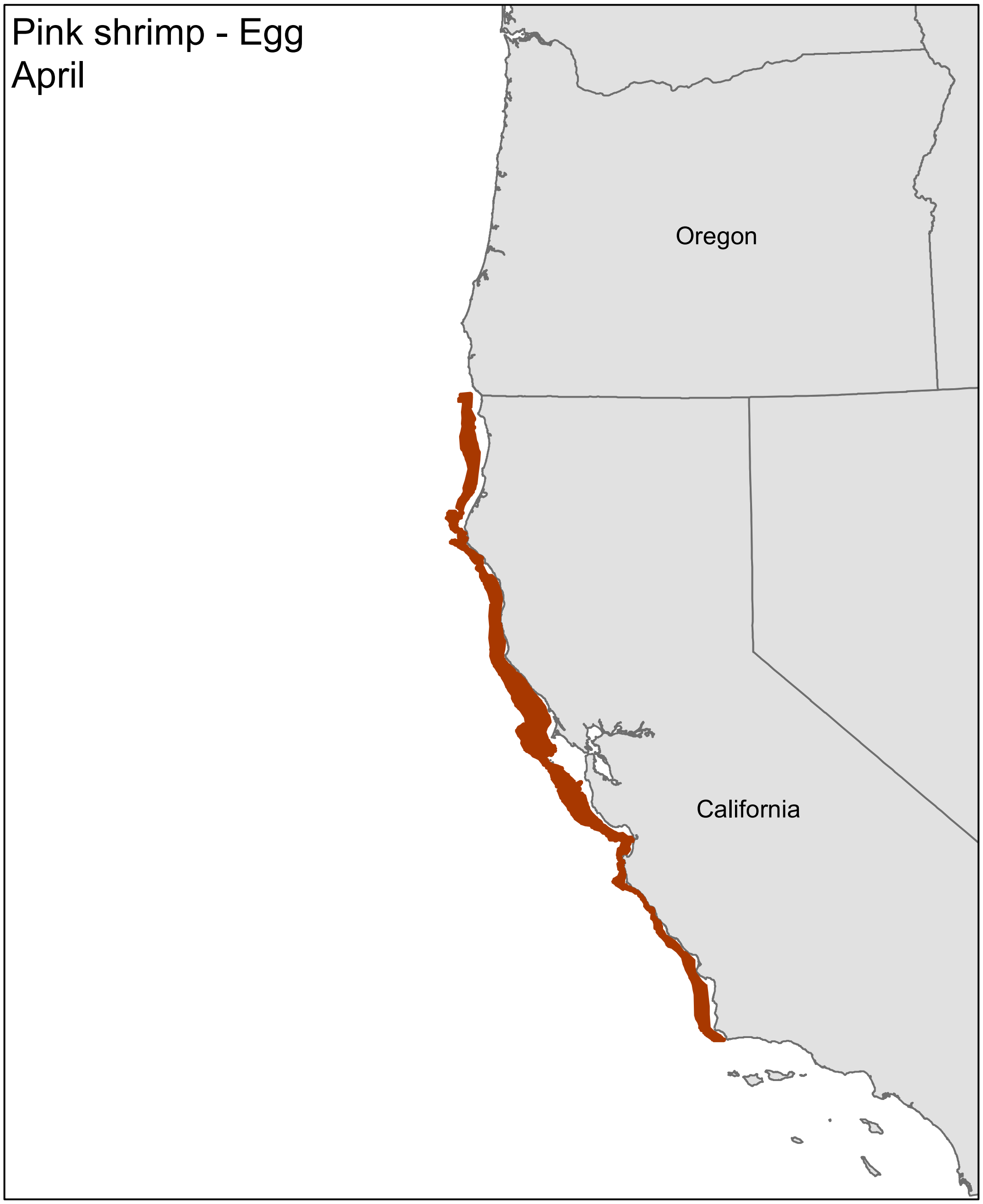

Fig A17. Pink shrimp egg distribution, April.

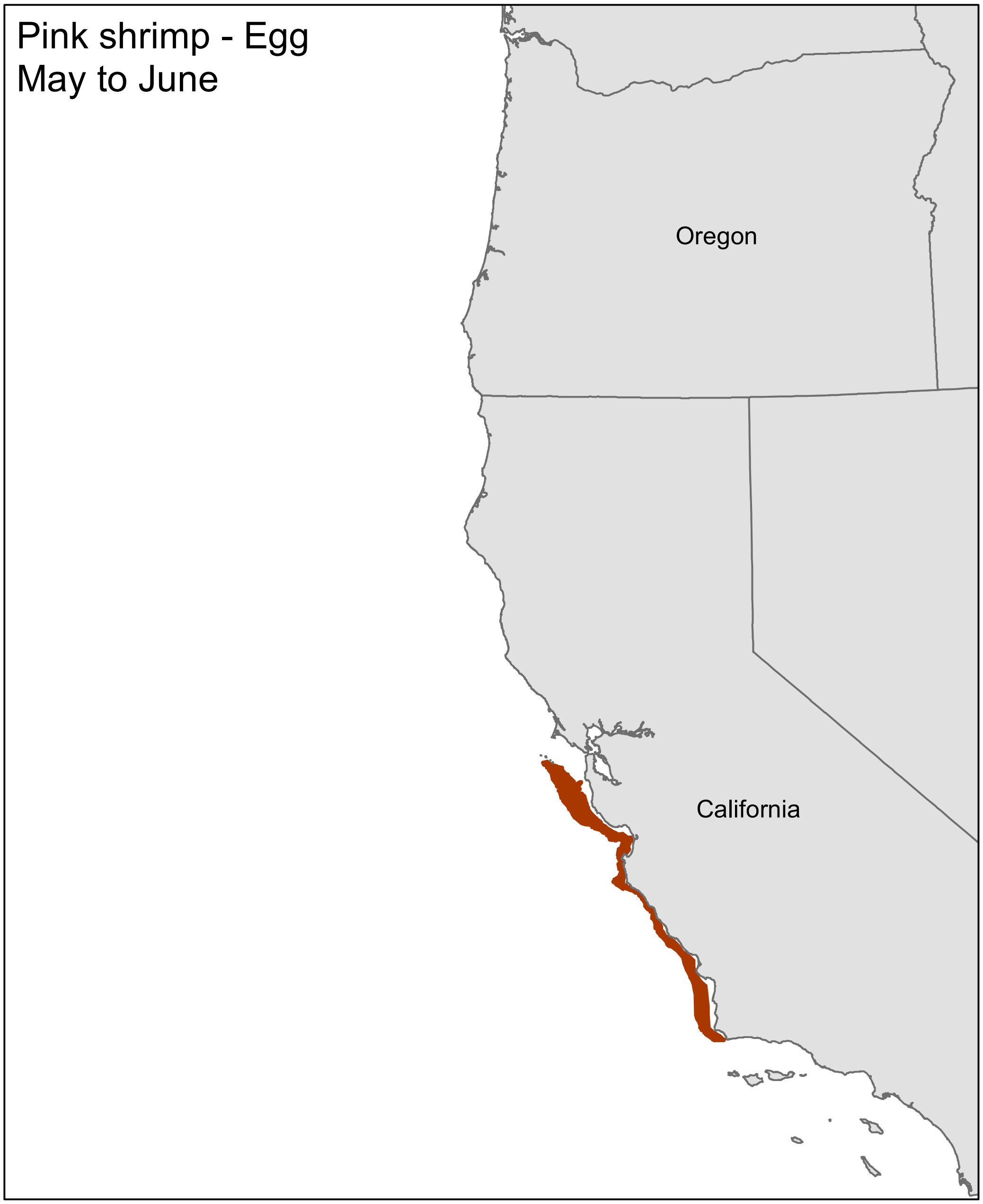

Fig A18. Pink shrimp egg distribution, May and June.

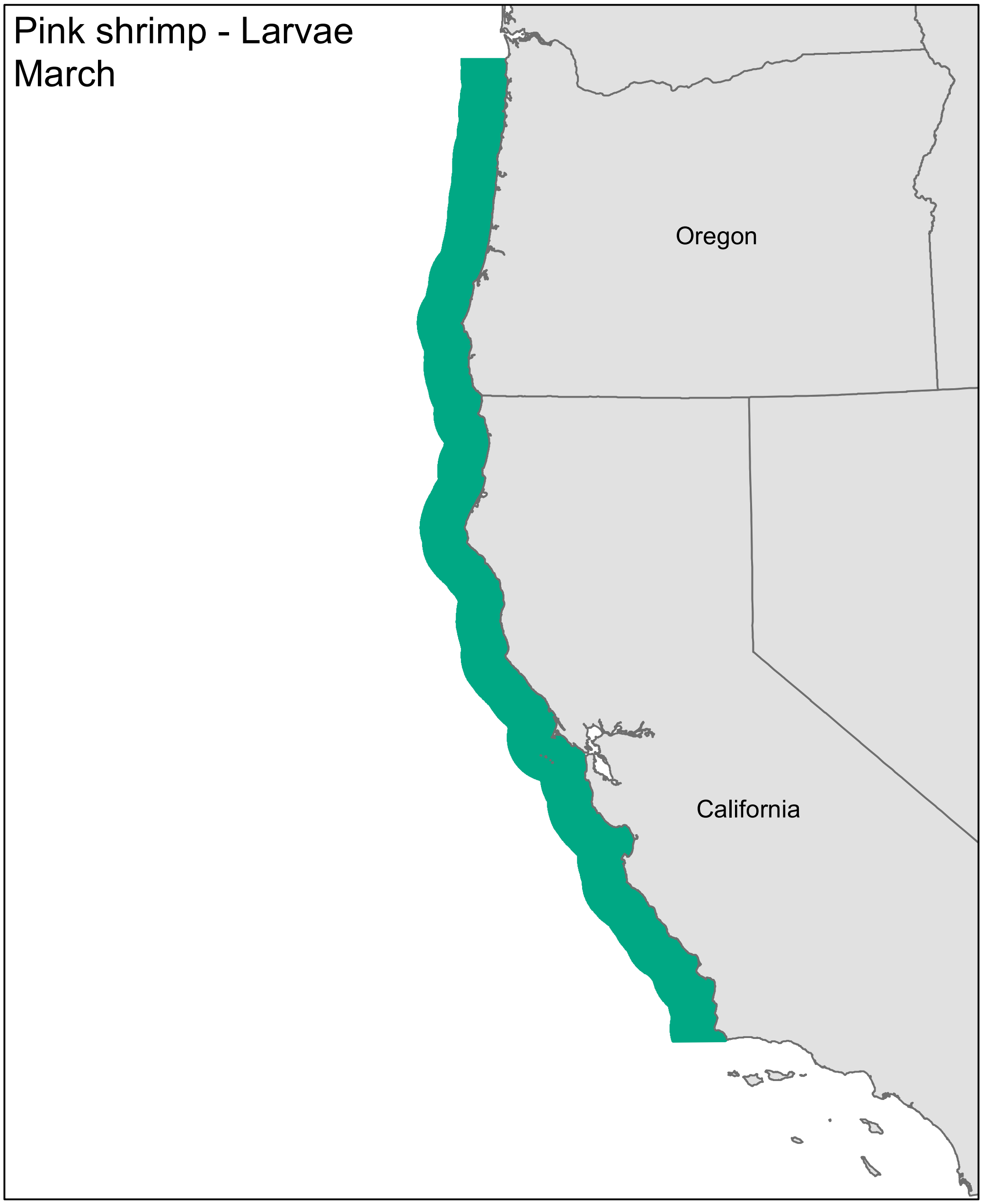

Fig A19. Pink shrimp larval distribution, March

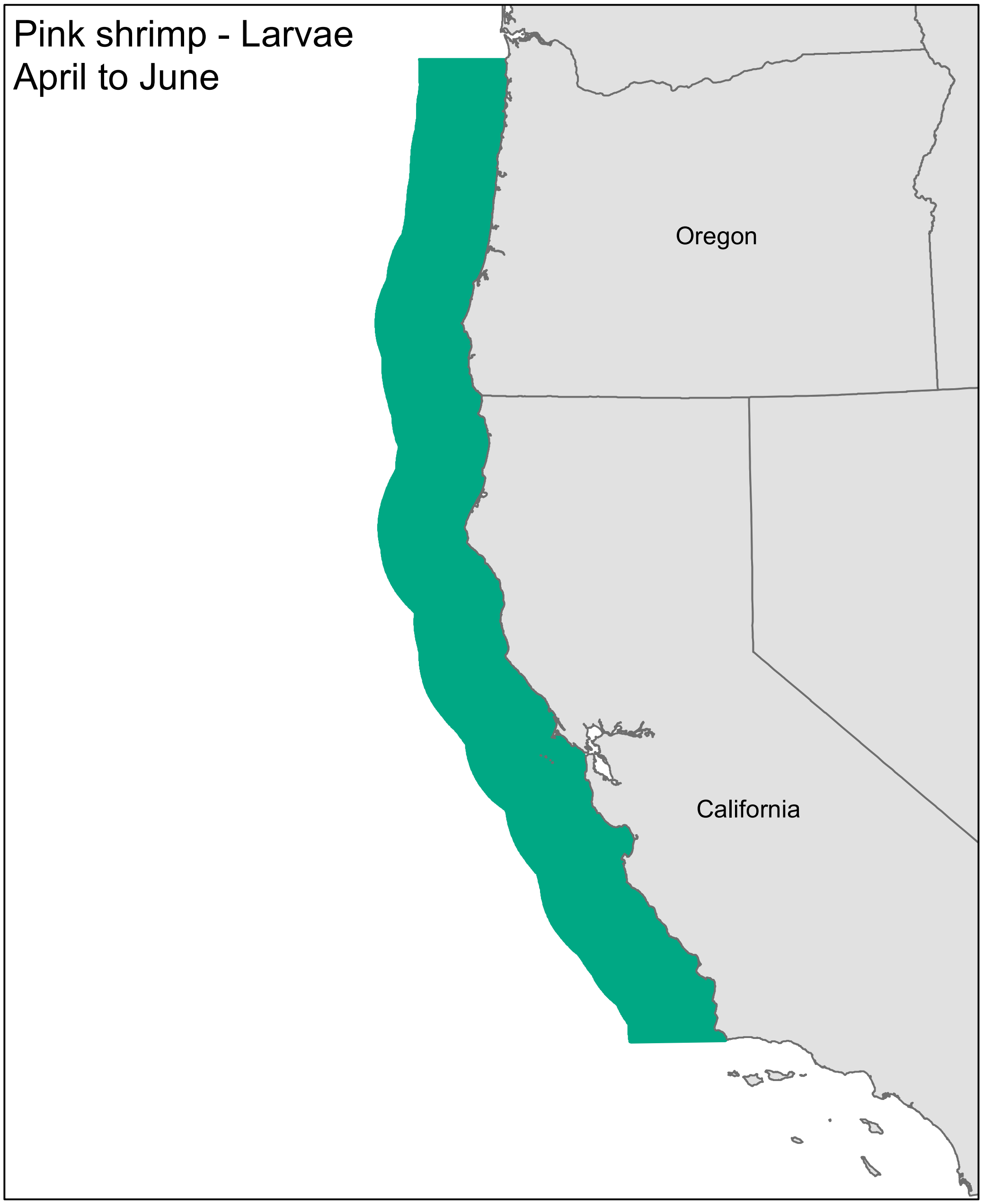

Fig A20. Pink shrimp larval distribution April through July.

Pacific hake, *Merluccius productus,* maps

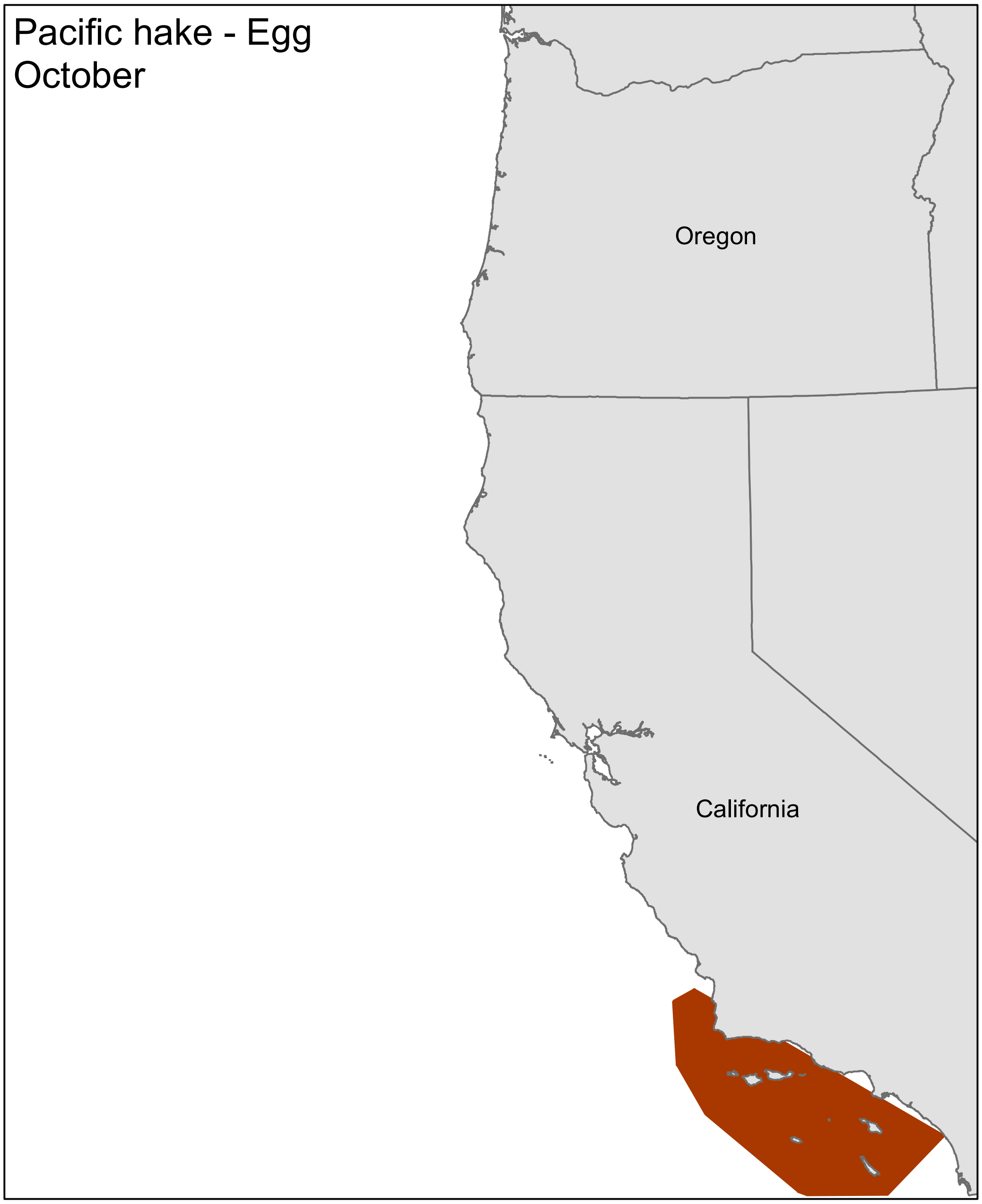

Fig A21. Pacific hake egg distribution, October.

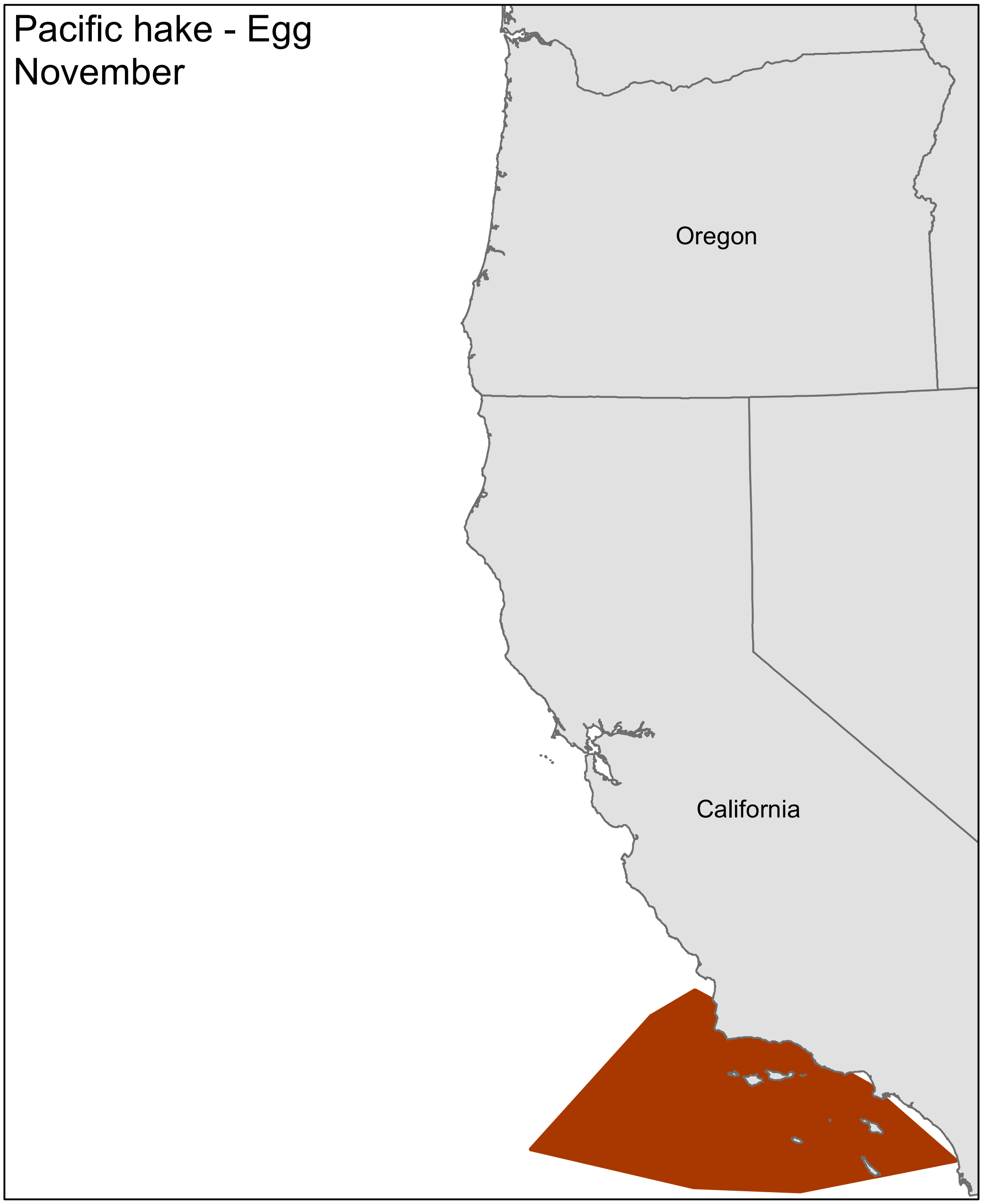

Fig A22. Pacific hake egg distribution, November

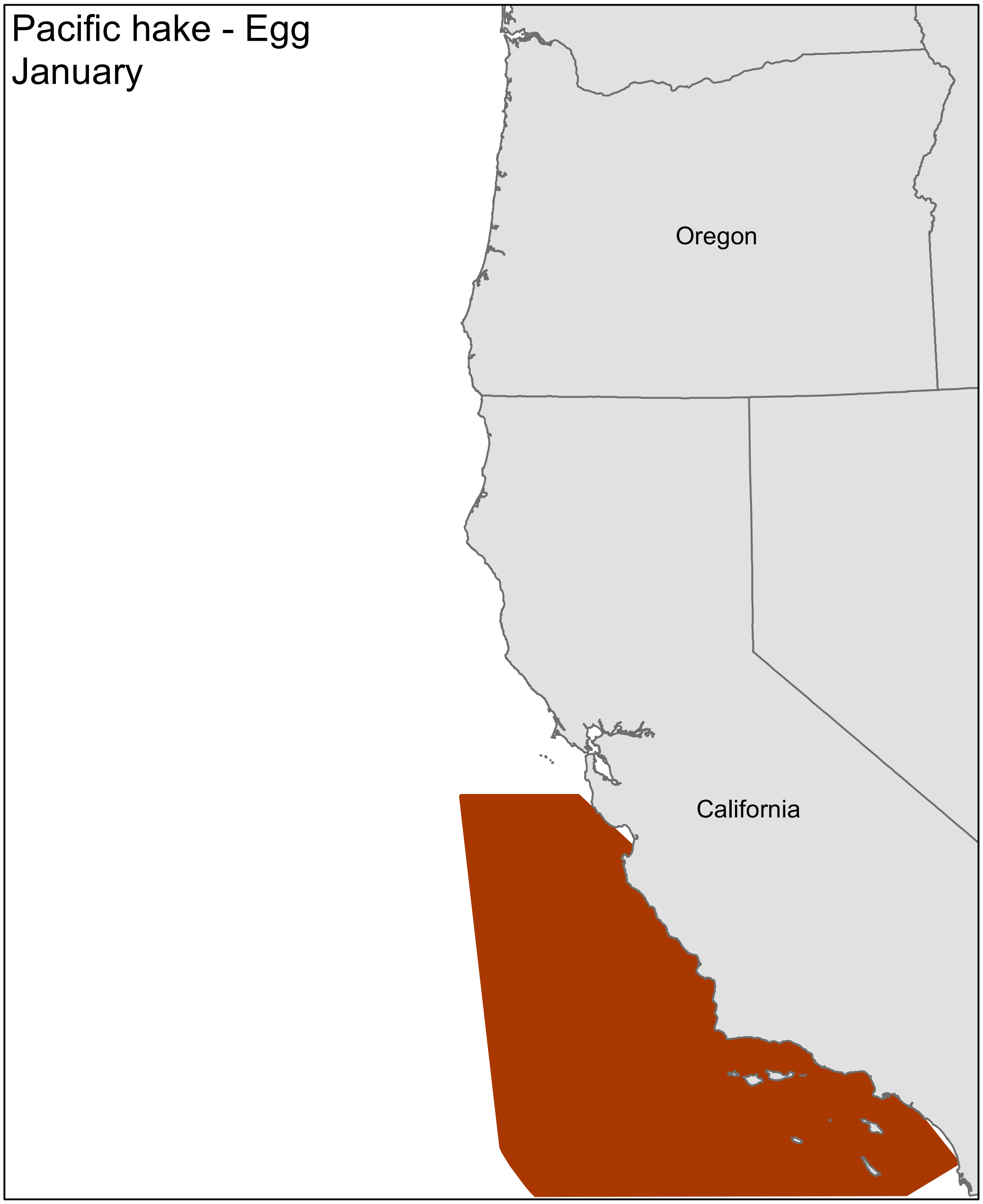

Fig A23. Pacific hake egg distribution, January. This map was used to represent December as well as there was not enough to map their distribution then. No difference in overall exposure found when November or January were used for December.

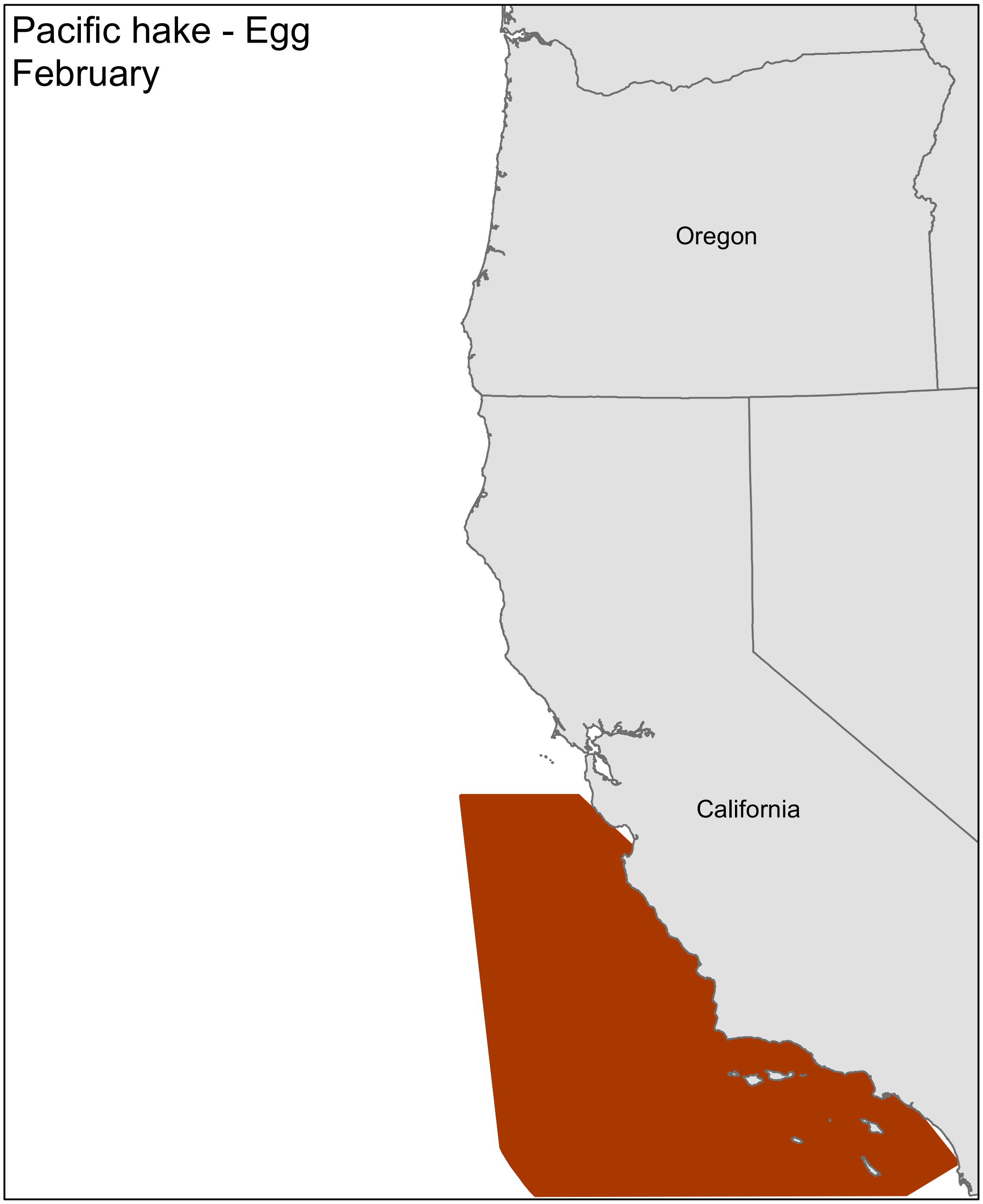

Fig A24. Pacific hake egg distribution, February.

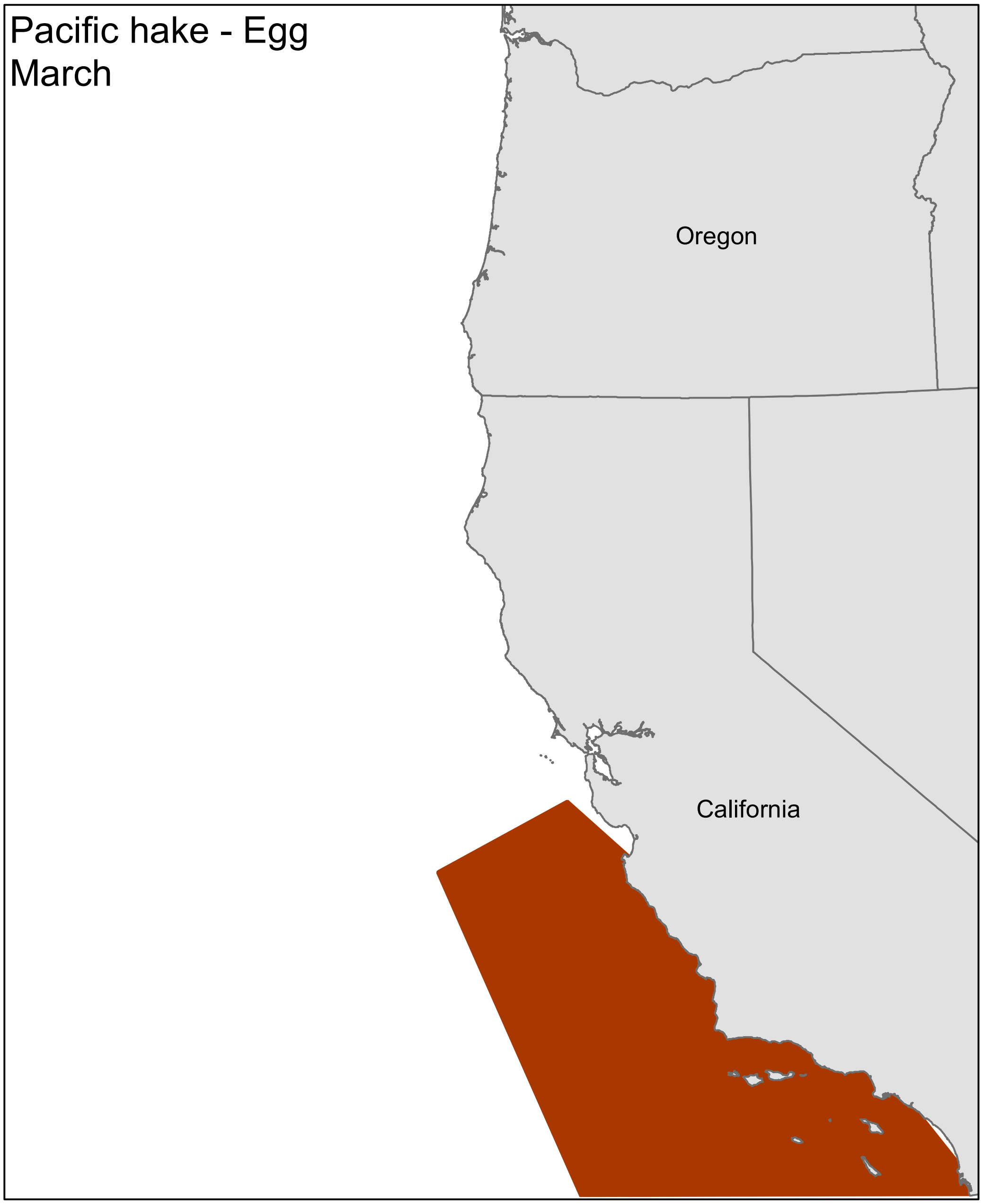

Fig A25. Pacific hake egg distribution, March.

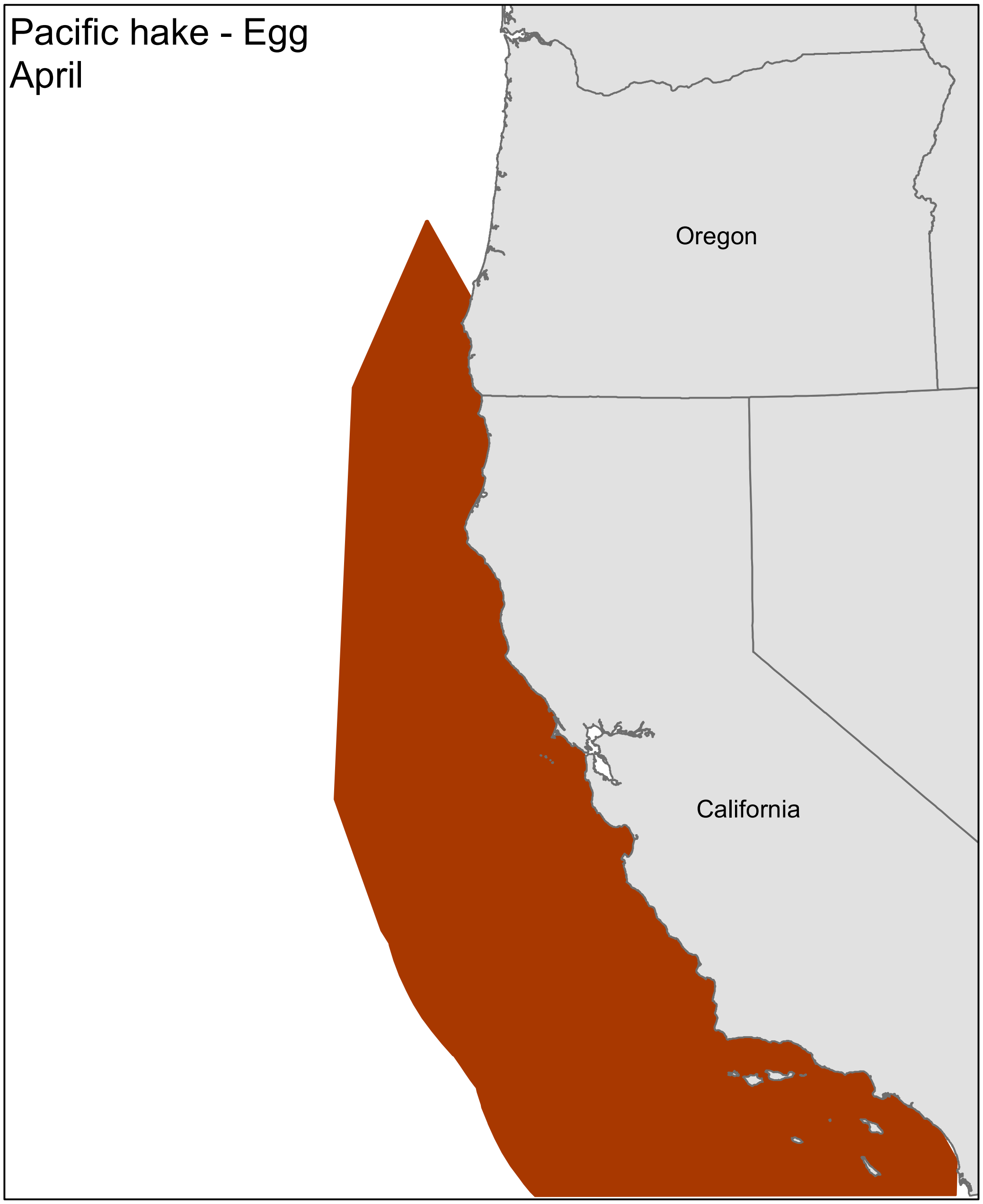

Fig A26. Pacific hake egg distribution, April.

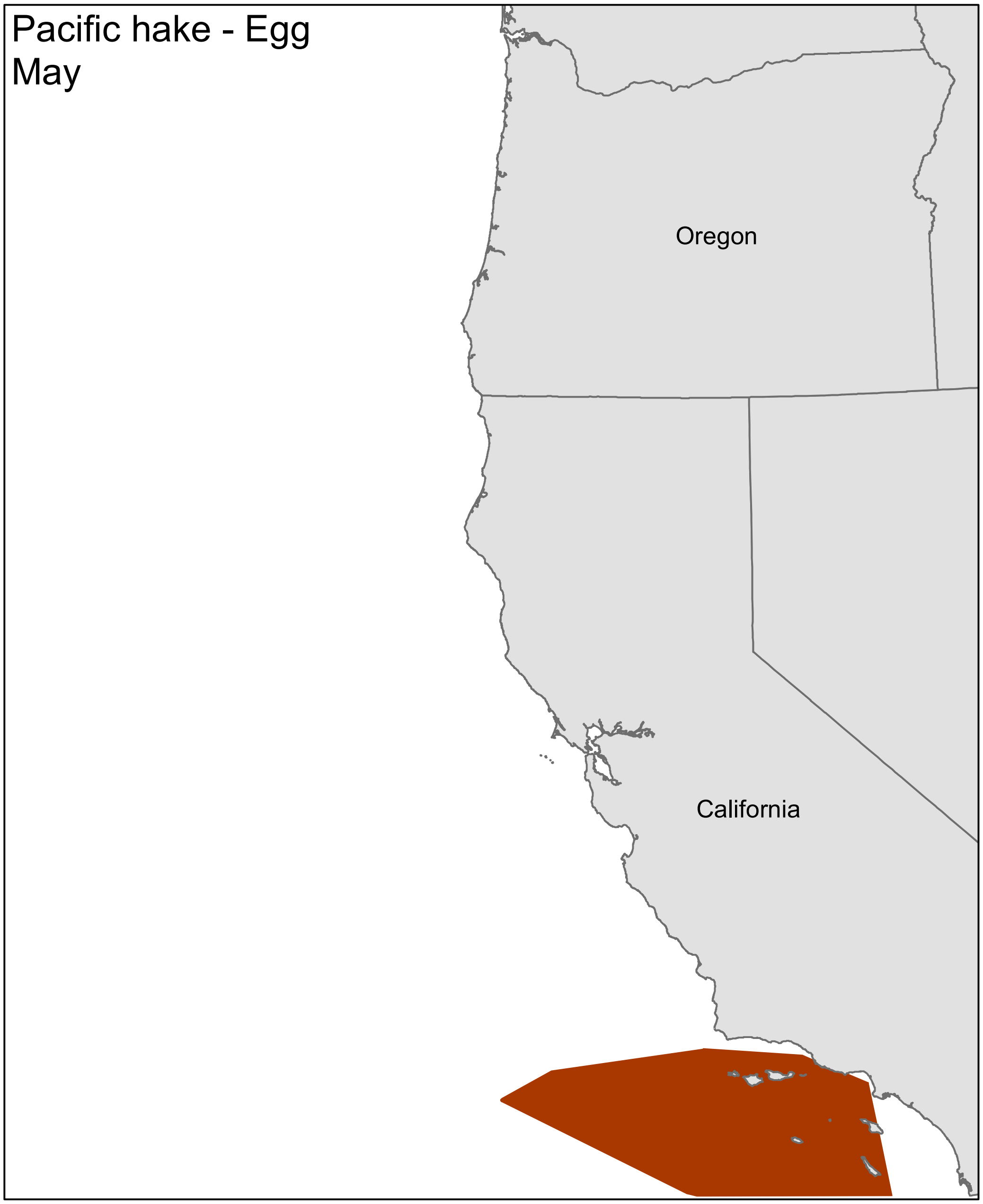

Fig A27. Pacific hake egg distribution, May.

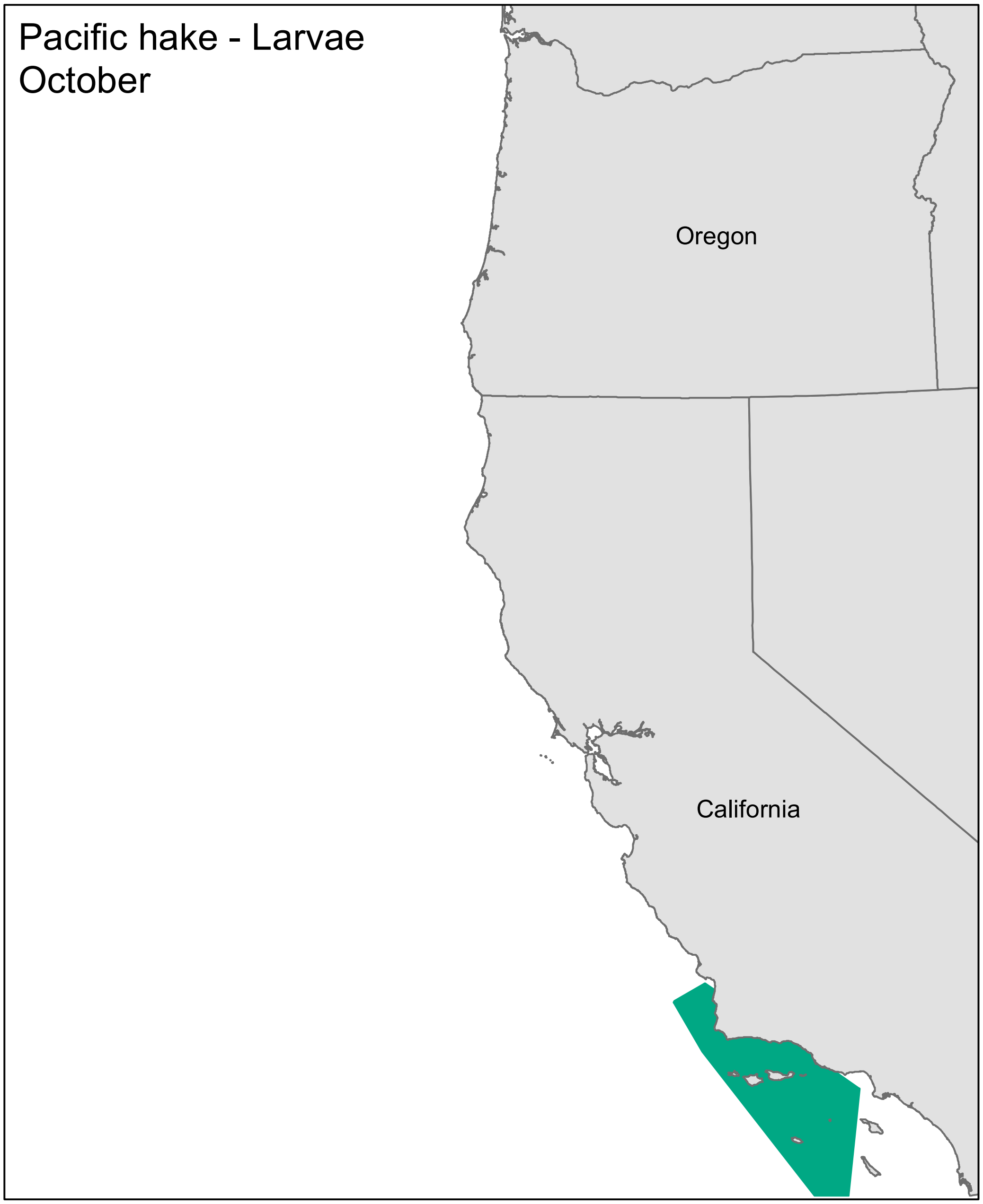

Fig A28. Pacific hake larval distribution, October.

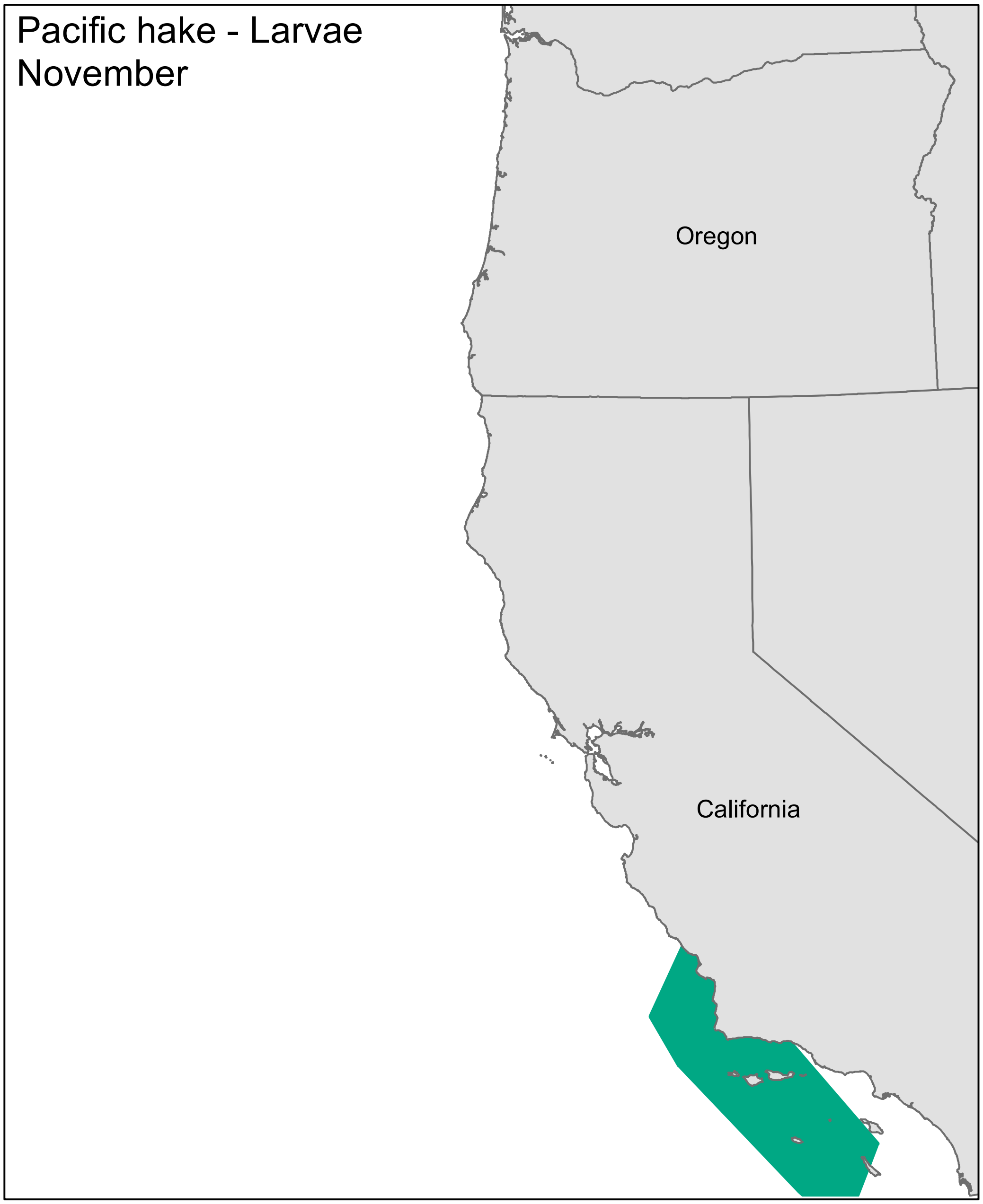

Fig A29. Pacific hake larval distribution, November.

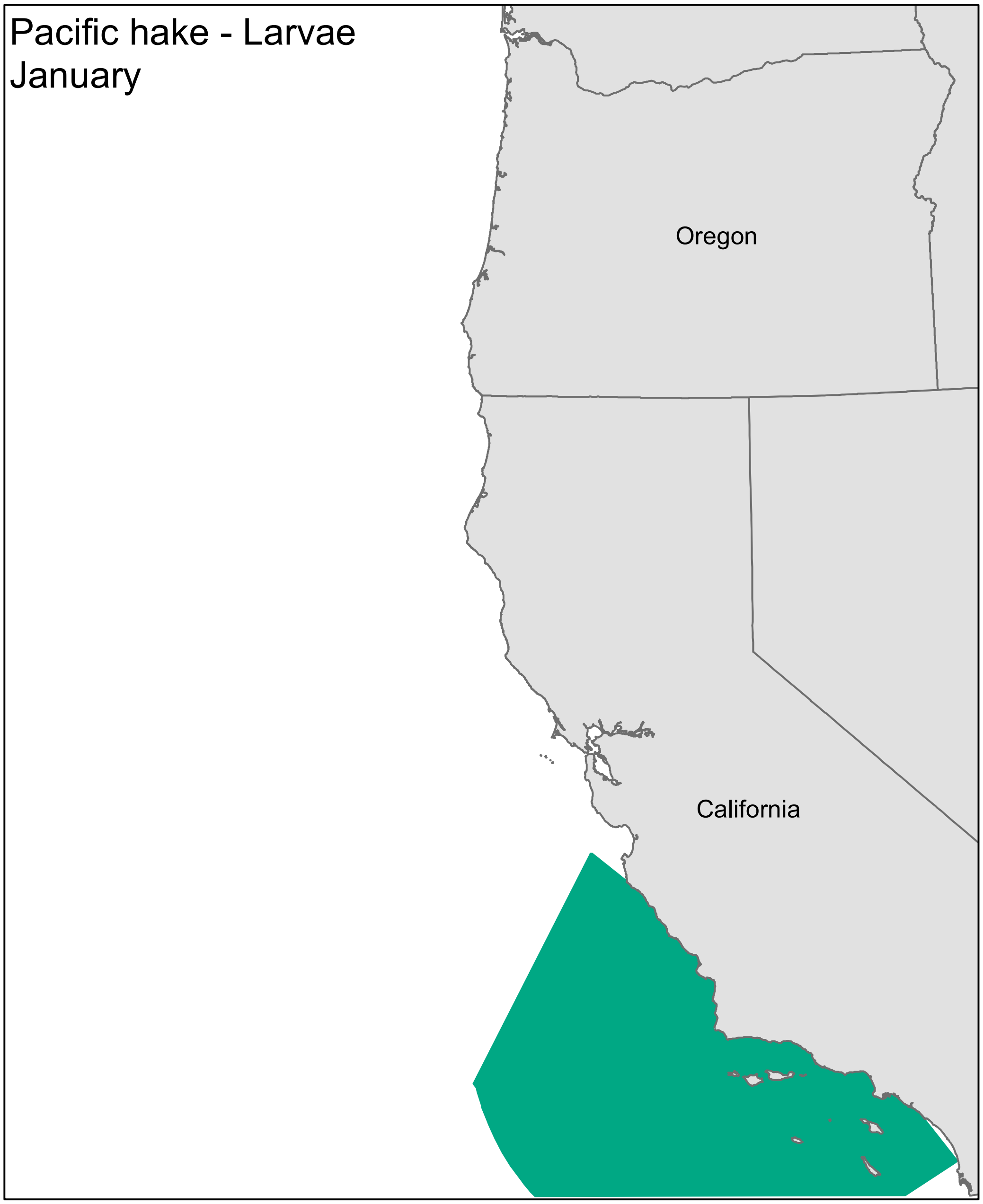

Fig A30. Pacific hake larval distribution, January. This map was used to represent their distribution in December as well.

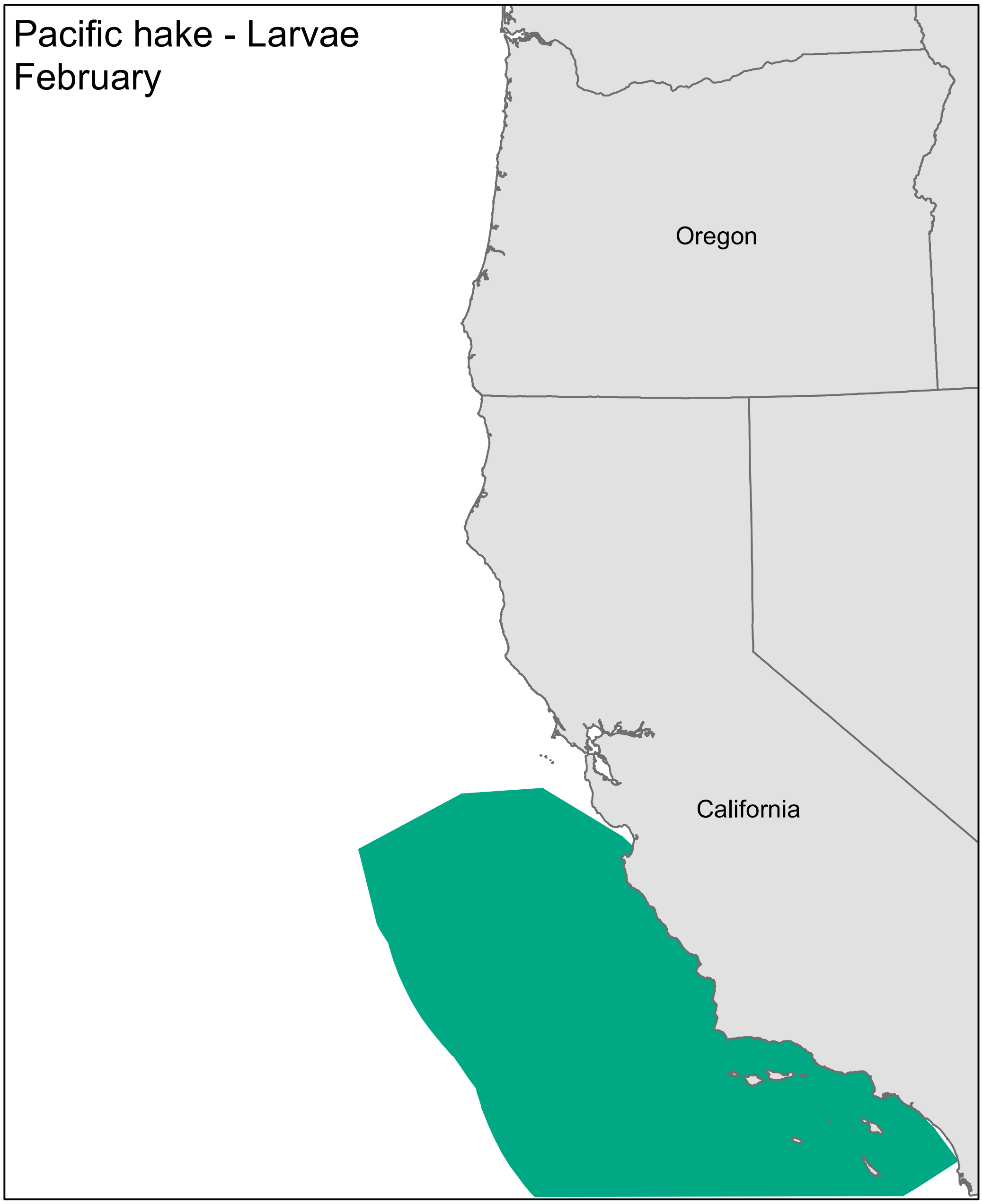

Fig A31. Pacific hake larval distribution, February.

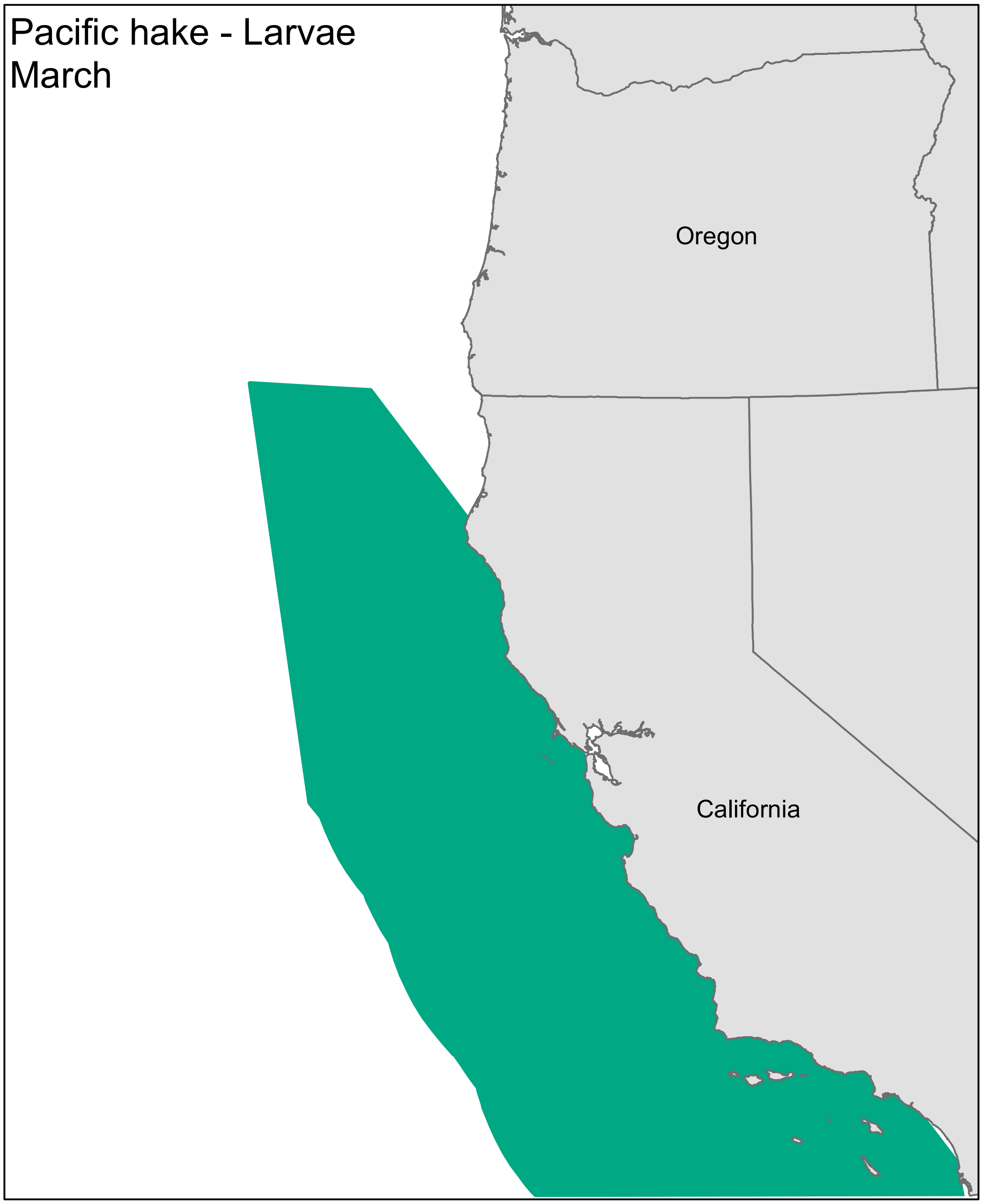

Fig A32. Pacific hake larval distribution, March.

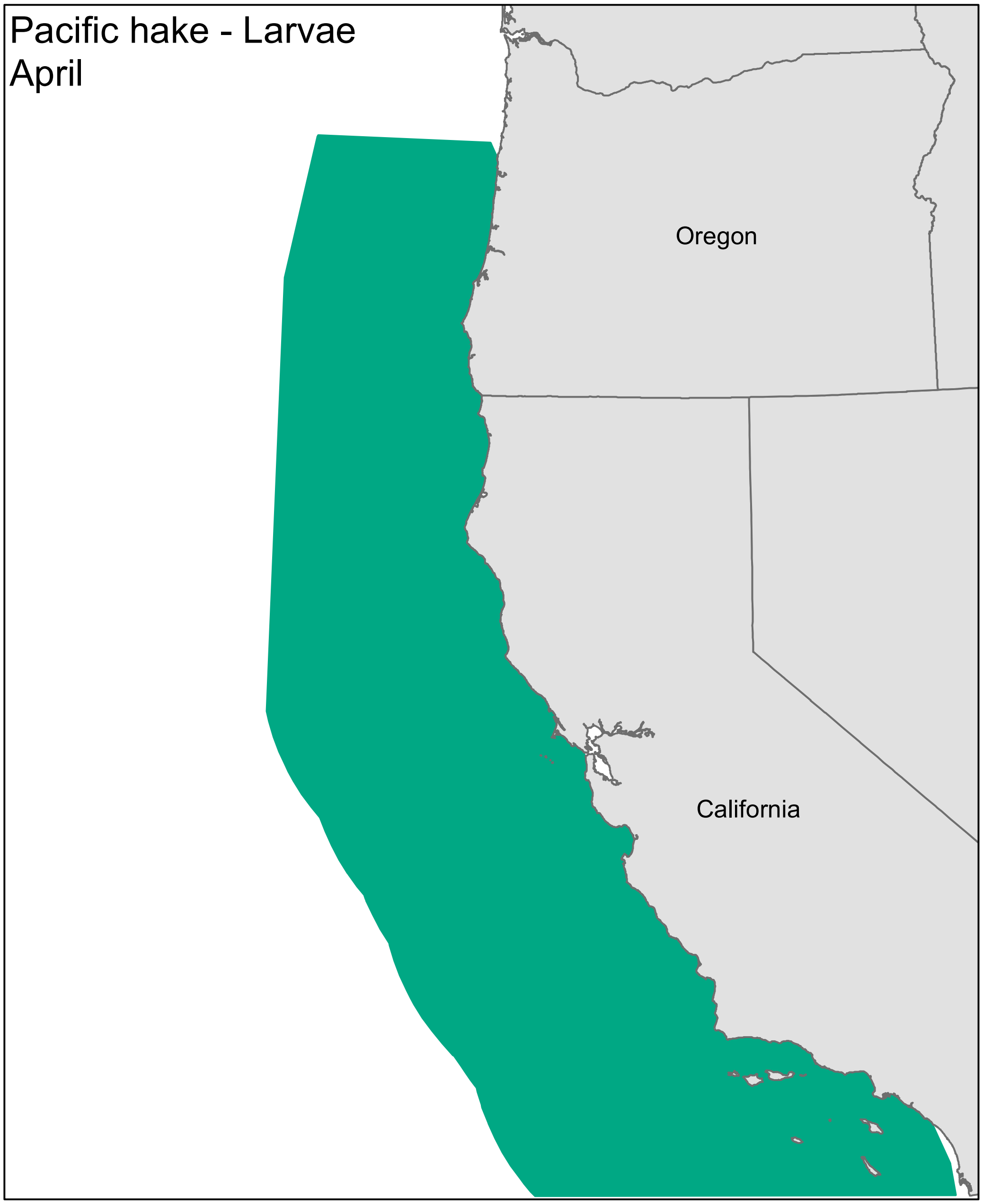

Fig A33. Pacific hake larval distribution, April. This distribution goes very far north – which may only occur during some years, however truncating the distribution had no effect on exposure calculated.

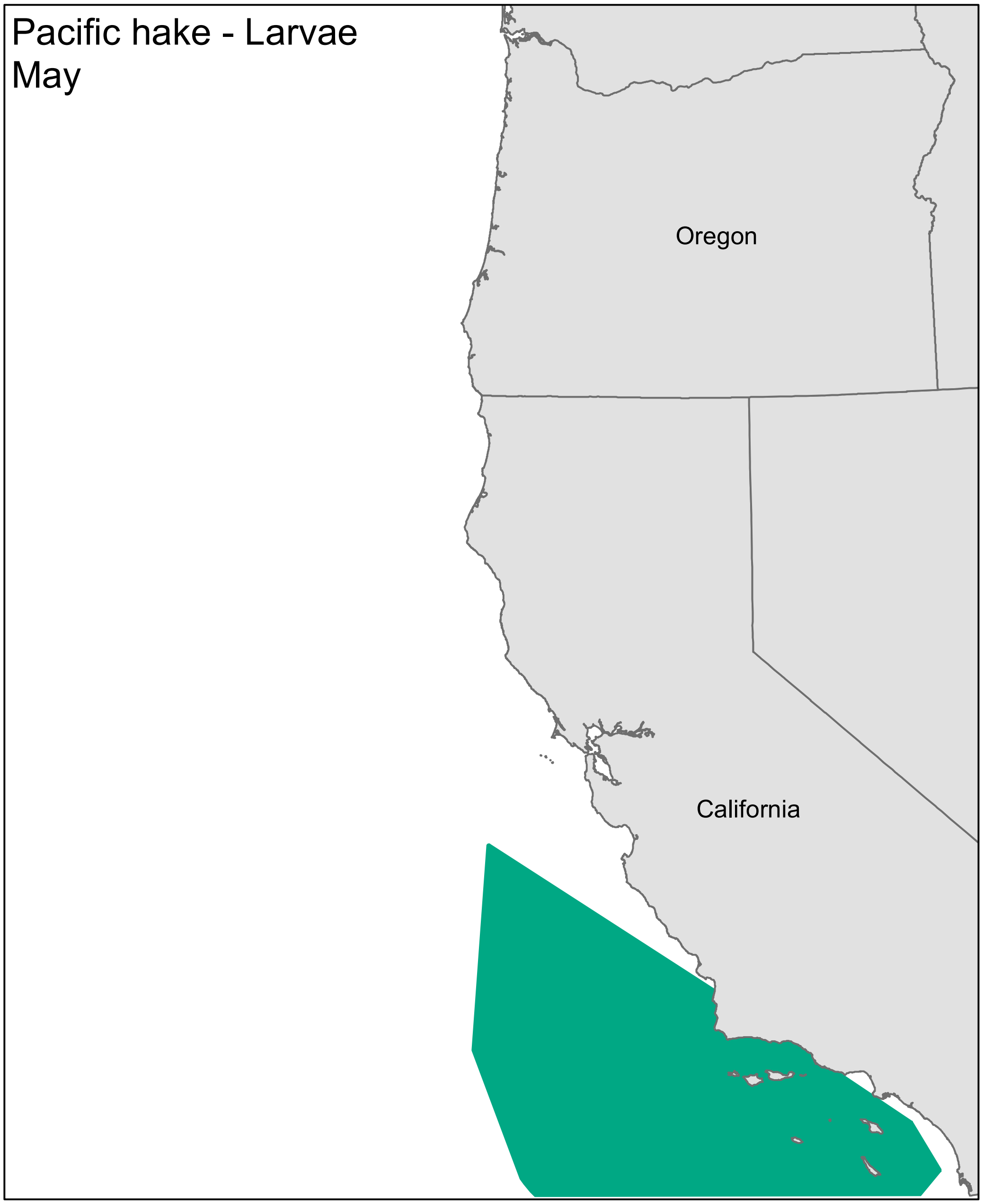

Fig A34. Pacific hake larval distribution, May.

Krill, *Euphausia pacifica*, maps

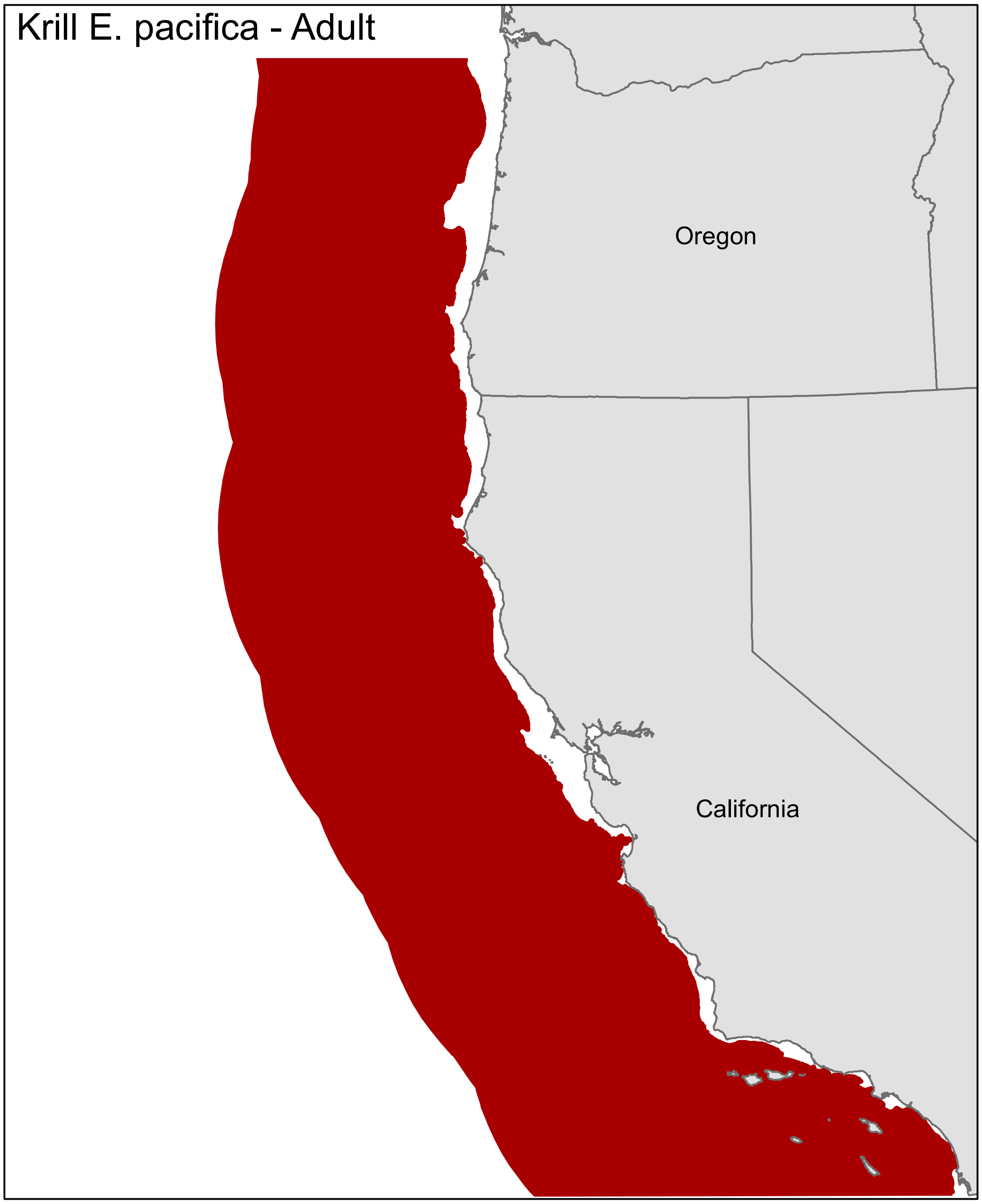

Fig A35. Krill *E. pacifica* adult, sub-adult and juvenile distribution – all months of the year.

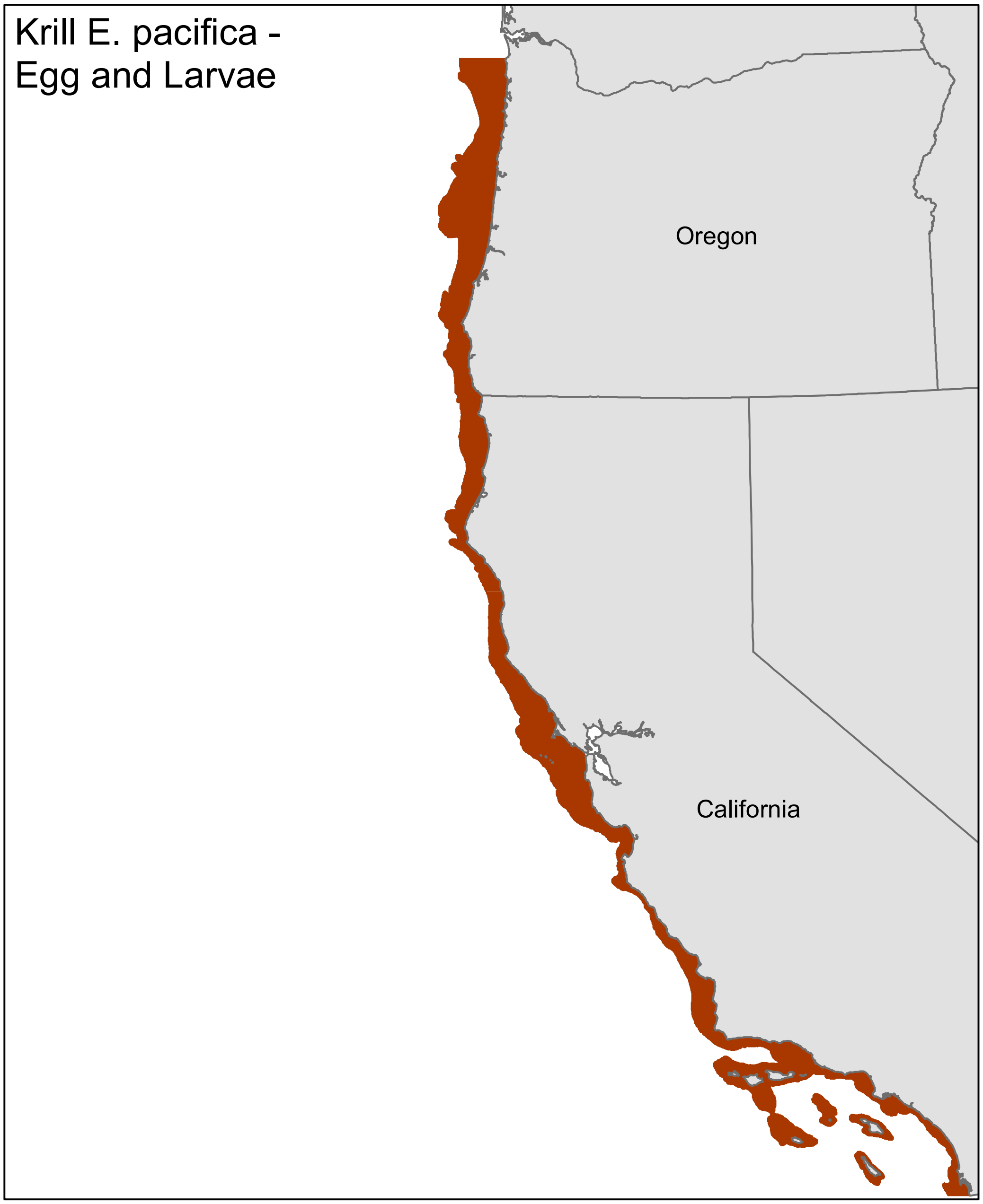

Fig A36. Krill *E. pacifica* egg and larval distribution (on shelf) months they are present.

Krill, *Thysanoessa spinifera*, maps

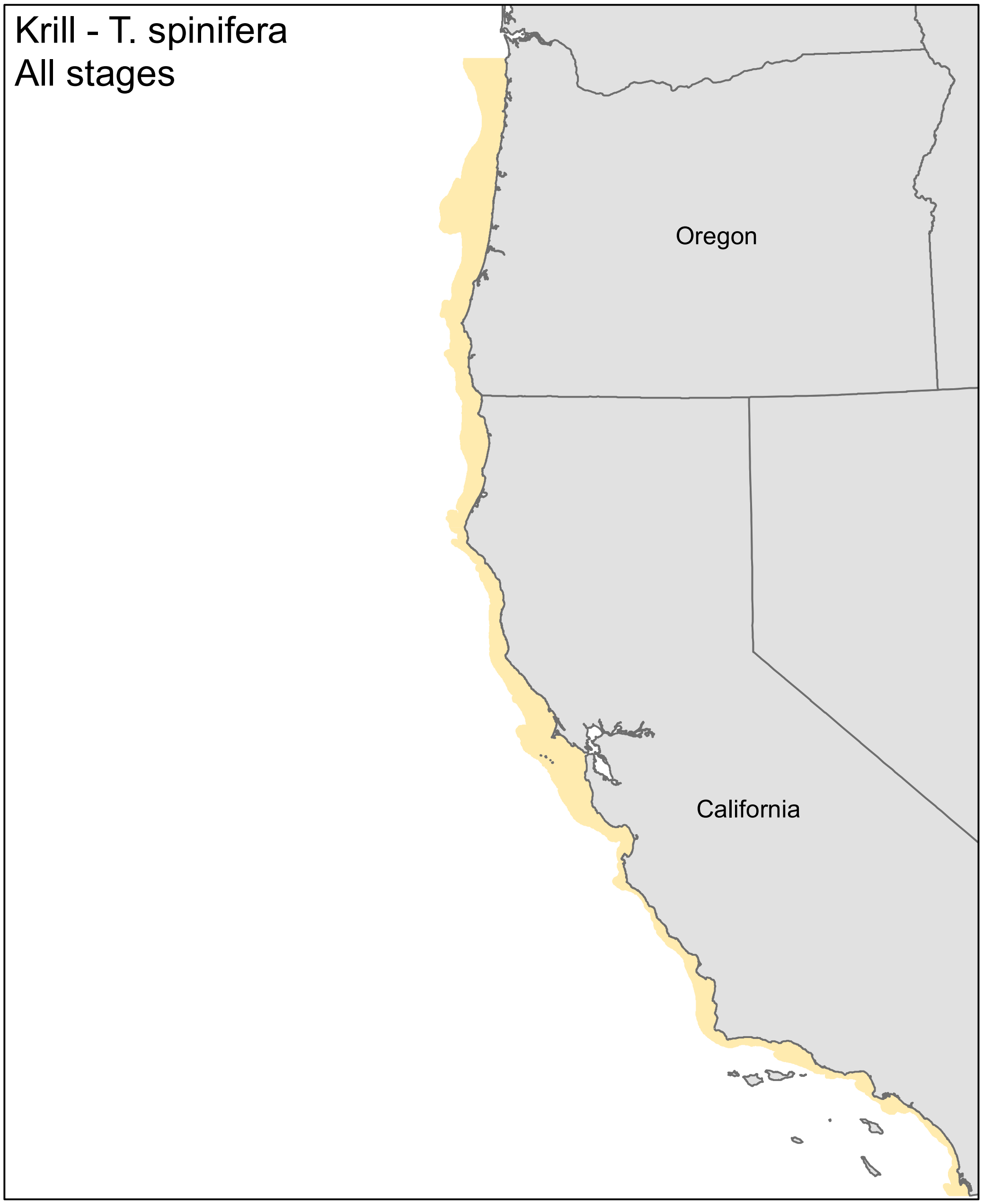

Fig A37. Krill *T. spinifera* distribution of all stages during months they are present (on shelf).

Pteropod, *Limacina helicina*, maps

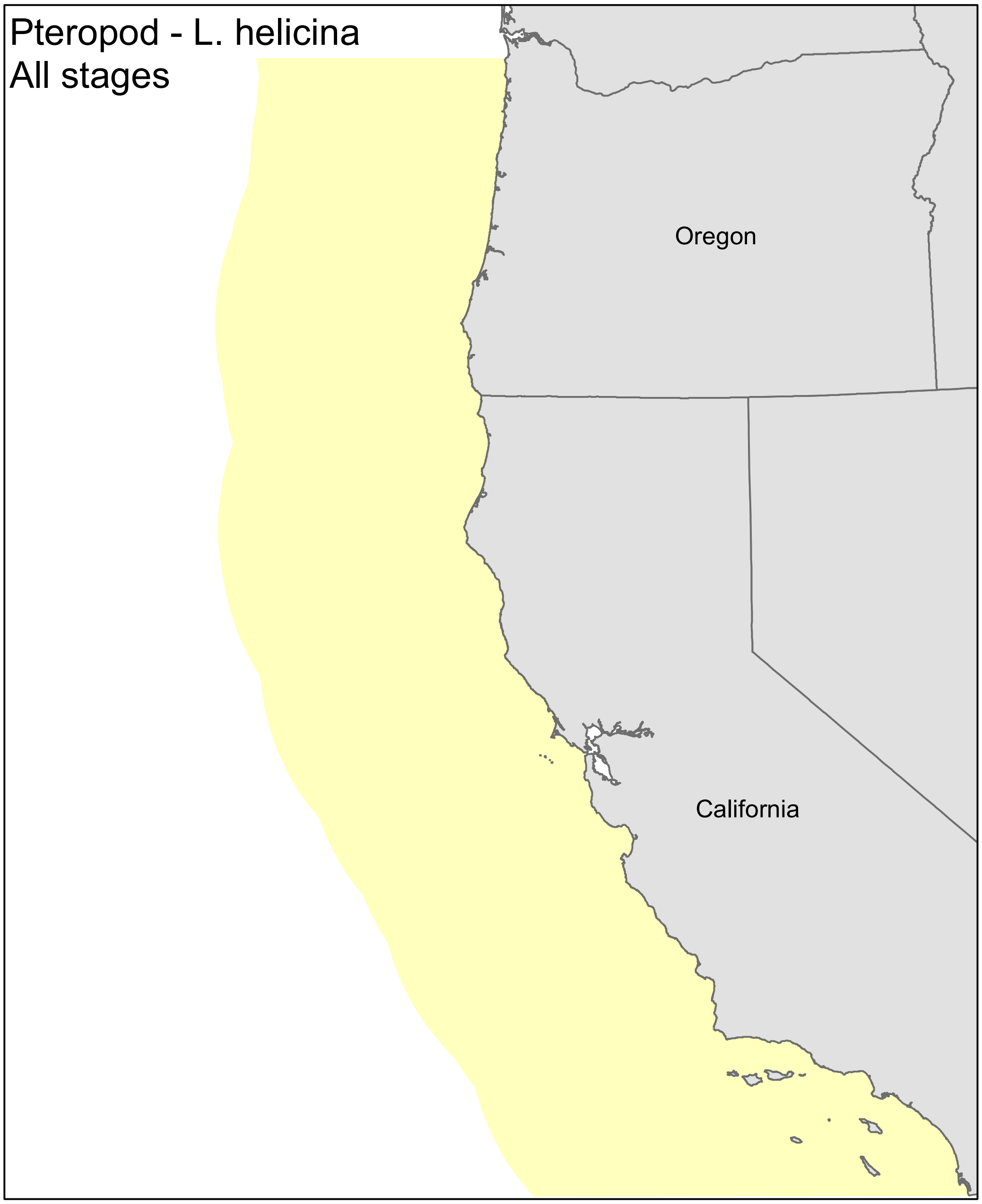

Fig A38. Pteropod *L. helicina* distribution of all stages, during months they are present.

Appendix B: Sensitivity of exposure estimates to form of pH exposure function

We tested the sensitivity of our conclusions to the shape of the pH exposure function in the process of translating exposure to particular pH values into a single exposure estimate. We considered two main approaches that bracket the range of possible effects. The first example assumed a sigmoidal relationship between consequence and pH. The second example assumed that adverse effects are minimal until pH drops below a threshold value, below which effects increase to some maximum level. This latter method was ultimately reported in the paper, but here we document the results for the sigmoidal relationship as well. In addition to these functional forms, we also considered alternative placement of these curves along the pH axis. For sigmoidal relationships, we adjusted the pH at which the exposure curve was one-half its maximum value; while for threshold relationships we simply adjusted the pH of the threshold (called the *critical* pH henceforth in the Appendix).

We explored three relationships between pH and relative consequence, and three critical pH values. The three functional forms (Fig B1 A and B) include: (1) *threshold*, (2) *sigmoid50%* for which the pH level that has no effect exceeds the critical pH by 50% (3) *sigmoid100%*; pH level that has no effect exceeds the critical pH by 100%. Note that since pH is in log space, to increasing pH by 50% simply involves adding 0.176 to the critical pH and to increasing by 100% involves adding 0.301.The three critical pH values used were 7.6, 7.65 and 7.7 (Fig B1).

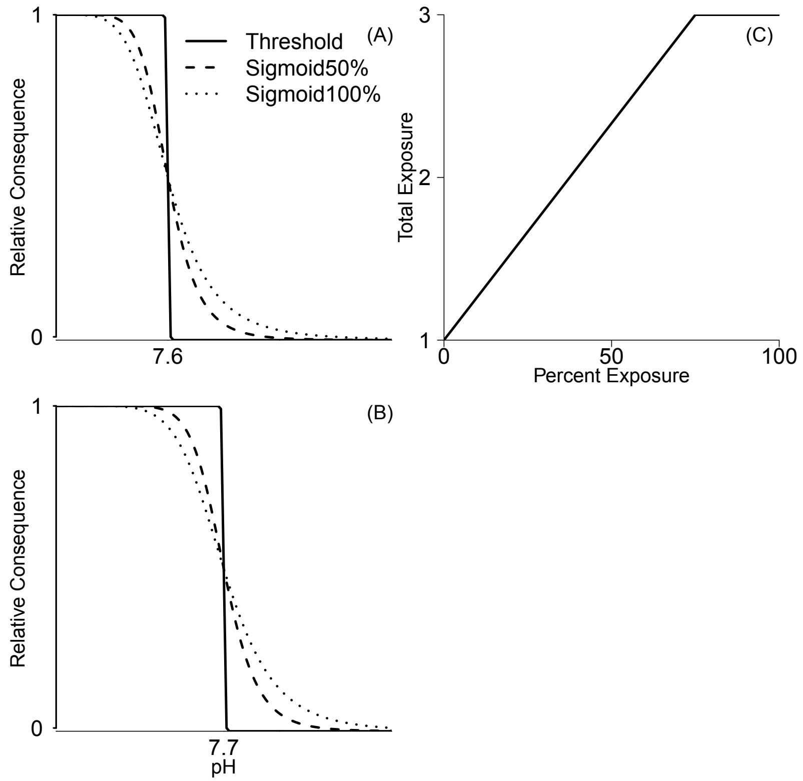

Fig B1. Determining exposure from pH values experienced. Showing the relationship between pH and relative consequence for three functional forms (A) and (B), at a critical pH of 7.6 (A) and 7.7 (B), and conversion between percent exposure and final total exposure estimate (C).

<u>Results from this sensitivity test:</u>

Exposure estimates were sensitive to the exposure function, producing a wide range of exposure estimates for each species' life history stage (Table B1). The critical pH had a larger effect on exposure estimates than the form of the exposure function (Fig B2 A and B). For example, changing critical pH from 7.6 to 7.65 increased the median exposure from 1.74 to 2.70 while changing the critical pH to 7.7 led to a median exposure of 3.00 across all species and life stages (Fig B2 A). In other words, if the critical pH was

high, then there was widespread exposure, regardless of the shape of the exposure function. In contrast, the median estimated exposure was nearly identical across functional forms (*sigmoid100* 2.29, *sigmoid50* 2.29, *threshold* 2.39) (Fig B2 B). We did however find some sensitivity to the shape of the exposure function at the lowest critical pH (Fig B2 C). This sensitivity arose because the threshold model predicts little to no exposure for many species at critical pH 7.6. However, at that pH the sigmoid curves allow for partial exposure from pH values that are slightly above the critical. For example, very little of the distribution of eggs and larvae for *L. helicina* will experience pH at or below the critical 7.6 (Fig B3) so the threshold model predicts low exposure (1.04). Conversely, substantial portions of the distribution are predicted to experience pH slightly above the critical (Fig B3) and under the sigmoid models these get partial exposure scores (1.48 and 1.68 for sig50 and sig100 respectively, Table B1).

This provided a forum to test the sensitivity of our conclusions to the form of the relationship. We found that the shape of the curve (sigmoidal versus threshold) had much less of an influence on estimates of exposure than did the value of the critical pH and thus only presented results for the threshold relationship. Other stressors might not exhibit the same lack of sensitivity to functional form, and including variation in the shape of the curve in addition to the critical would only add a source of uncertainty.

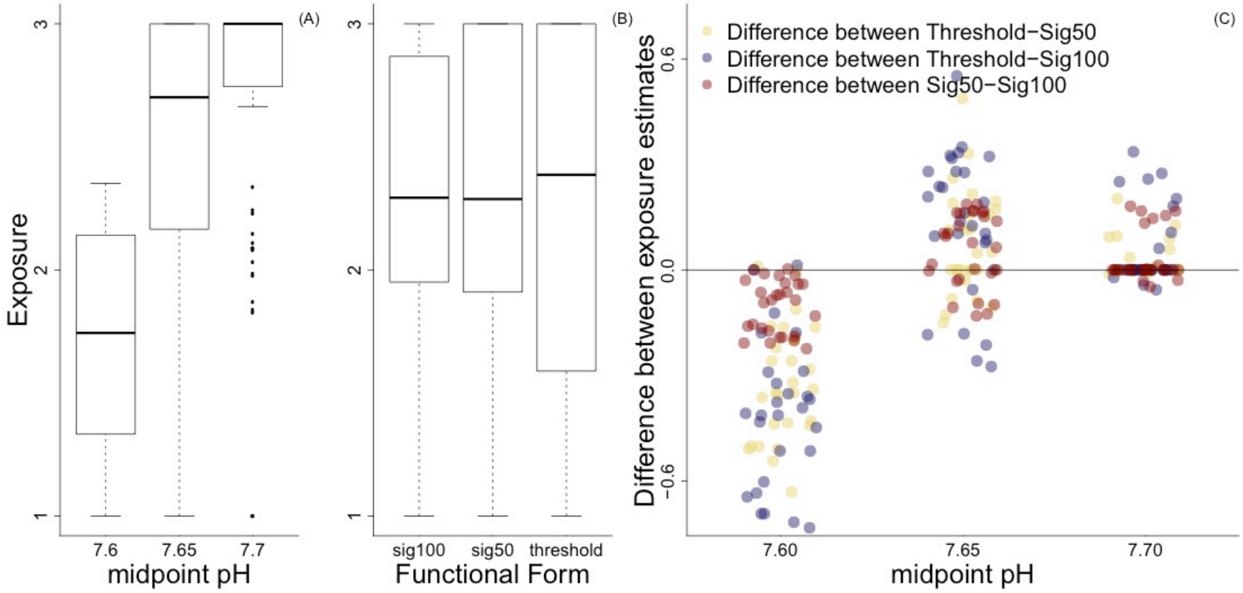

Fig B2. Comparison of exposure estimates when using different mid-point pH values. Comparison is across all species and life stages (A), different forms of the functional relationship (B) and a plot of the differences in exposure estimates when comparing pairs of the three methods used (C).

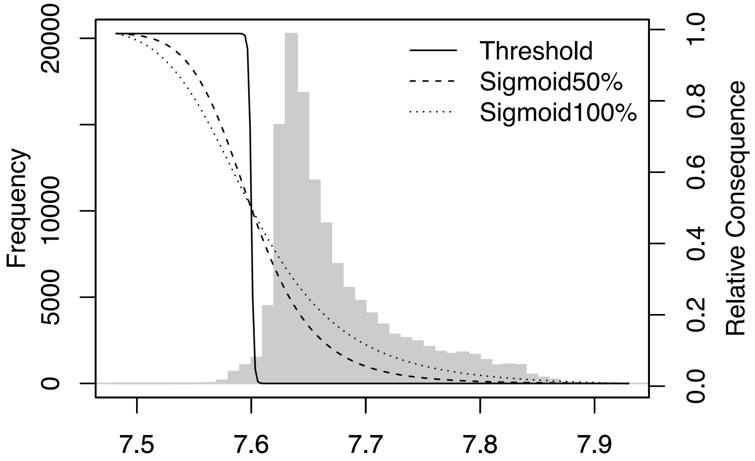

Fig B3. Histogram of pH values experienced by *L. helicina* larvae/eggs. Different functional forms of the relationship between pH and relative consequence are plotted on top assuming critical pH 7.6.

Table B1. Exposure estimates using all model functional forms and midpoints.

Species	Life Stage	Thresh mid 7.6	Thresh mid 7.65	Thresh mid 7.7	Sig50 mid 7.6	Sig50 mid 7.65	Sig50 mid 7.7	Sig100 mid 7.6	Sig100 mid 7.65	Sig100 mid 7.7
Metacarcinus magister	Eggs	1.67	3.00	3.00	2.11	2.95	3.00	2.18	2.76	3.00
	Larvae	1.03	1.43	2.09	1.23	1.58	2.08	1.39	1.70	2.11
	Megalops	1.00	1.39	1.98	1.19	1.51	1.98	1.35	1.65	2.03
	Juvenile i	1.49	3.00	3.00	2.00	2.81	3.00	2.09	2.65	3.00
	Juvenile ii	1.49	3.00	3.00	2.00	2.81	3.00	2.09	2.65	3.00
	Adult	1.49	3.00	3.00	2.00	2.81	3.00	2.09	2.65	3.00
Pendalus jordani	Eggs	1.90	3.00	3.00	2.22	3.00	3.00	2.26	2.84	3.00
	Larvae	1.04	2.61	3.00	1.55	2.28	3.00	1.76	2.28	2.82
	Juveniles	1.89	3.00	3.00	2.17	2.95	3.00	2.21	2.77	3.00
	Adult	1.89	3.00	3.00	2.17	2.95	3.00	2.21	2.77	3.00
Limacina helicina	Eggs & larvae	1.04	2.24	3.00	1.48	2.12	2.80	1.68	2.15	2.66

	Juvenile	1.03	2.42	3.00	1.53	2.21	2.87	1.72	2.21	2.73
	Subadult	1.04	2.56	3.00	1.58	2.30	2.97	1.77	2.28	2.80
	Adult	1.04	2.42	3.00	1.54	2.23	2.90	1.74	2.23	2.75
Merluccius productus	Eggs	1.01	3.00	3.00	1.64	2.51	3.00	1.86	2.45	3.00
	Larvae	1.06	2.15	3.00	1.50	2.18	2.91	1.71	2.21	2.74
	Adult	1.00	1.00	1.00	1.00	1.00	1.00	1.00	1.00	1.00
Euphausia pacifica Early spawners	Eggs	1.67	3.00	3.00	2.03	2.85	3.00	2.11	2.68	3.00
	Larvae	1.02	1.37	1.83	1.18	1.46	1.84	1.31	1.55	1.87
	Juveniles	1.99	3.00	3.00	2.25	3.00	3.00	2.28	2.87	3.00
	Sub-adults	2.35	3.00	3.00	2.34	3.00	3.00	2.34	2.92	3.00
	Adult	2.13	3.00	3.00	2.29	3.00	3.00	2.30	2.90	3.00
Euphausia pacifica Late spawners	Eggs	1.59	3.00	3.00	2.02	2.85	3.00	2.11	2.68	3.00
	Larvae	1.02	1.59	2.34	1.28	1.69	2.24	1.45	1.80	2.23
	Juveniles	2.19	3.00	3.00	2.30	3.00	3.00	2.31	2.90	3.00
	Sub-Adults	1.87	3.00	3.00	2.23	3.00	3.00	2.26	2.86	3.00
	Adults	2.13	3.00	3.00	2.29	3.00	3.00	2.30	2.90	3.00
Thysanoessa spinifera	Larvae	1.71	3.00	3.00	2.05	2.83	3.00	2.12	2.67	3.00
	Juveniles	1.02	1.52	2.15	1.25	1.61	2.09	1.40	1.70	2.09
	Adult	1.74	3.00	3.00	2.09	2.89	3.00	2.16	2.72	3.00

Appendix C: Life stage consequences

Table C1. Consequence rankings and citations. Provided for each species' life history stage used in the assessment.

Species	Life Stage	Consequence	Citations	Justification	Consequence Uncertainty
Metacarcinus magister	Eggs	*1*	(Miller 2015)	There are no papers directly on the eggs of this species, Miller did some preliminary experiments and found no impact on survival, however experiments were not standardized. Experiments on other crab species have found no impact on growth but longer time to hatching (Long et al. 2013)	3
	Larvae	*3*	(Christmas 2013, Descoteaux 2014, Miller 2015)	Descoteaux found a small impact on survival in their low pH 7.4-7.6 experiment and possibly at intermediate pH ~7.75 Christmas also found in a five day experiment – no impact on feeding rate or survival but changes in movement patterns – low pH at 7.5. Miller 2015 found a 2.5-3 fold decline in survival of larvae in waters with pH 7.5 and 7.1	2
	Megalops	*3*	Miller unpublished results, conclusions from larvae	Miller also did some preliminary experiments on megalops and found an impact on survival, but with no published literature, we gave them the same response to OA as larvae, but with higher uncertainty.	3
	Juvenile i	*3*	Miller unpublished results	In Miller's preliminary experiment, juveniles from the first instar onwards appeared to show some coping capacity, but this is highly uncertain.	3
	Juvenile ii	*1*	NA	Juveniles have a second phase after survival through the first year and since this stage is	3

				getting more similar to adults, we assumed their sensitivity to OA to be more similar to adults – but again high uncertainty	
	Adult	*1*	(Pane and Barry 2007, Hans et al. 2014)	Adult crabs show clear ability to buffer in hypercapnia situations, as do other crab species	1
Pendalus jordani	Eggs	*1*	(Arnberg et al. 2012)	No impact on hatching success after two weeks in pH 7.6 for *P. borealis*	2
	Larvae	*2*	(Bechmann et al. 2011, Arnberg et al. 2012)	Both the cited papers on *P. borealis* found no impact on mortality but an increase in development and pers. comm. with both primary authors suggest a rating of 2 for consequence, a pH=7.6 was used.	2
	Juvenile	*1*	NA	The sensitivity of shrimp juveniles was assumed to be equivalent to that of adults, but ranked as highly uncertain	3
	Adult	*1*	(Hammer 2012)	Hammer found *P. borealis* to be able to buffer low pH waters, pH=6.86 was used, and an ability to tolerate such low levels demonstrates clear tolerance	2
Limacina helicina	Eggs & larvae	*3*	(Bednaršek et al. 2012)	There are no papers on the eggs and larvae of *L. helicina* so we assumed their sensitivity level to be the same as later life stages of the same species – recognizing that we do not have as high confidence in this conclusion. Pers. comm. Nina Bednarsek	2
	Juvenile	*3*	(Bednaršek et al. 2012, Bednaršek et al. 2014)	Juveniles are the most studied with clear dissolution of shells when in undersaturated waters. Undersaturation in the system occurd at pH=7.7 in Bednarsek 2012	1
	Subadult	*3*	(Bednaršek et al. 2012, Bednaršek et al. 2014)	Subadults were also collected in the California Current analysis and found to have strong shell dissolution in undersaturated	1

				waters.	
	Adult	*2*	(Bednaršek et al. 2012, Bednaršek et al. 2014, Busch et al. 2014)	Busch et al. (2014) found shell dissolution in adult pteropods in undersaturated conditions, but only a severe impact on mortality in waters highly unsaturated, demonstrating some tolerance in a 5-week period to undersaturation. As well, from conversations with Nina Bednarsek, adults may be sensitive like earlier stages, but have a thicker external organic layer with slight resistance to OA.	2
Merluccius productus	Eggs	*1*	(Munday et al. 2009, Bignami et al. 2013, Frommel et al. 2013, Frommel et al. 2014)	Numerous papers have been published on different teleost fish species, none of which are on the same genus as hake. Some are cited here, and conclusions were made through literature review and recommendations from experts (pers. comm. Phil Munday and Sean Bigmani)<br>Most have found no impact on fish eggs.	3
	Larvae	*2*	(Munday et al. 2009, Bignami et al. 2013, Frommel et al. 2013, Frommel et al. 2014)	Fish larvae tend to show more variable responses, with some having no effect, some showing increase in otolith size and some with clear impacts on larval growth and organ development.	3
	Adult	*1*	(Bromhead et al. , Melzner et al. 2009, Kroeker et al. 2010)	Although adult coral reef fish have shown some behavioral responses to OA, we assume no real impact on Pacific hake adults. Example paper nothing this: Bromhead et al. (2014)	1
Euphausia pacifica Early and late spawners	Eggs	*1*	[Cite McLasky paper once it is out – shortly], (Kawaguchi et al. 2011, Kawaguchi et al. 2013)	Kawaguchi did not find any impact on hatching success for *E. superba*. McLaskey preliminary results also found no change in hatching success of eggs	2
	Larvae	*2*	[Cite McLasky paper once	McLaskey found a delay in development	2

			it is out – shortly]	time, but no clear impact on survival, thus a value of 2.	
	Juveniles	*2*	(Saba et al. 2012)	Assumed to be same as adults	3
	Sub-adults	*2*	(Saba et al. 2012)	Assumed to be same as adults	3
	Adult	*2*	(Saba et al. 2012)	This paper was on *E. superba* and the authors found that there was an increase of both ingestion and metabolism suggesting that there could be a negative consequence because of increasing energy requirements	3
Thysanoessa spinifera	Eggs	*1*	[Cite McLasky paper once it is out – shortly], (Kawaguchi et al. 2011, Kawaguchi et al. 2013)	All *T. spinifera* consequences were assumed to match *E. pacifica*	3
	Larvae	*2*	[Cite McLasky paper once it is out – shortly]	All *T. spinifera* consequences were assumed to match *E. pacifica*	3
	Sub-adults	*2*	NA	All *T. spinifera* consequences were assumed to match *E. pacifica*	3
	Adult	*2*	(Saba et al. 2012)	All *T. spinifera* consequences were assumed to match *E. pacifica*	3

Appendix D: Life history model parameters and details

***Dungeness crab,* Metacarcinus magister**

The model developed for Dungeness crab includes four life history stages: eggs, larvae, juveniles and adults. Other models of Dungeness crab model the population of males and females separately (McKelvey et al. 1980), however, to maintain a simple approach, this model includes both males and females grouped together. Life stage parameters were obtained from the literature and are listed with their sources in Table 1.

Table D1. Parameter values for four Dungeness crab life history stages, and their sources. Note that survival values are rounded to 4 decimal places although more were used in the code.

Parameter	Values (daily)	Source	Notes (this column may be cut for publication)
e_{sc} = egg survival	0.9999 day^{-1}	(Shirley et al. 1987)	The authors found 98% survival over their 160-day experiment. Egg survival is likely high due to female retention of eggs.
e_{dc} = egg duration	122 days	(Shirley et al. 1987, Rasmuson 2013)	Eggs are found on females for ~4 months per of the year (Rasmuson 2013, Table 3.1). Eggs survived hatched after 42-160 days (Shirley et al. 1987).
l_{sc} = larval survival	0.9361 day^{-1}	(Hobbs et al. 1992)	Hobbs et al. (1992) found ~13% survival over one month through field estimates. Lab based estimates have also be found (Reed 1969). Others have used a combination of the two (Moloney et al. 1994), however, the field estimate was used here as lab estimates do not account for predation (Rumrill 1990), and many species predate on crab larvae.
l_{dc} = larval duration	122 days	(Rasmuson 2013)	Crab larvae are present in the water column for a range of 105-215 days off California and 89-143 off OR and WA (Rasmuson 2013). An assumed value of ~120 days was used for this analysis.

$j1_{sc}$ = juvenile 1 survival	0.9931 day^{-1}	(Stevens and Armstrong 1984, Wainwright et al. 1992)	There are two papers with estimates of juvenile survival which are highly different – Wainwright et al. (1992) estimates 8.1% survival per year, by regressing the logarithm of population abundance on age. From this method they found 5 estimates of annual survival were found with an average of 8.1%.<br>Alternatively Stevens and Armstrong (1984) write that since mortality rates are unknown, they use an assumed range of 0.5-0.8. Since the latter uses an assumed survival rate, the estimate from Wainwright et al. (1992) was used.<br><br>There is support fort this survival rate, and now this stage represents early settled megalopae and early juveniles, until they reach the next summer and are 1+ age
$j1_{dc}$	365 days	(P. Sean MacDonald pers. comm.)	This stage is present for the first year – from recommendations by P Sean McDonald.
$j2_{sc}$ = juvenile survival	0.9955 day^{-1}	(Wainwright et al. 1992, Armstrong et al. 2003)	Armstrong et al. (2003) write that the Wainwright paper reported survival from 1+ to 2+ as 19.5%
$j2_{dc}$ = juvenile duration	365 days	(Rasmuson 2013)	Fig 3.2 – they have lived for 1 year in the J1 stage and so now they are second year juveniles and last for another year before recruiting<br>QUESTION: does this last 12 months or 24???
a_{sc} = adult survival	0.9991 day^{-1}	(Higgins 1997, Higgins et al. 1997)	Higgens et al. (1997) report estimates of adult survival Table 1 that average to a value of 0.725. Some authors have suggested that males and females may have different

			survival rates (McKelvey et al. 1980) however the specific values are not known.
f_{ac} = adult fecundity	2739.73 day^{-1}	(McKelvey et al. 1980, Rasmuson 2013)	Females produce between 1.5-2.5 million eggs, thus the value of ~2 million was used, and divided by 12 to get a per month estimate, then by 2 to determine the number per female in the population (since it is a male and female model). Fecundity is not correlated to egg carapace width (Rasmuson 2013, citing: (Wickham 1979a, Wickham 1979c, Hankin et al. 1989))

Transition and Elasticity Matrices
Using values in Table 1. The final transition matrix is:

	Eggs	Larvae	Juvenile1	Juvenile2	Adults
Eggs	0.99	0	0	0	2739.73
Larvae	0.01	0.94	0	0	0
Juvenile1	0	0	0.99	0.00	0
Juvenile2	0	0	0	0.99	0
Adults	0	0	0	0	1.00

In order to ensure a null growth rate, $\lambda = 1$, all values in the transition matrix were multiplied by the multiplier 0.996786.

The output was the following elasticity matrix:

	Eggs	Larvae	Juvenile1	Juvenile2	Adults
Eggs	0.1556	0	0	0	0.0018
Larvae	0.0018	0.0251	0	0	0
Juvenile1	0	0.0018	0.1673	0	0

Juvenile2	0	0	0.0018	0.2042	0
Adults	0	0	0	0.0018	0.4387
**Sums**	**0.1574**	**0.0269**	**0.1691**	**0.2060**	**0.4405**

***Pink shrimp,* Pandalus jordani**

The life stage model for *Pandalus jordani*, pink shrimp, consists of four life history stages: eggs, larvae, juveniles, and adults. Males and females are both modeled in the same pool of individuals. The final life stage of adults currently lumps all reproductively mature individuals into one stage. This stage however, could be subdivided into two – of adult 1 and adult 2 – which would represent the fact that adult shrimp differ in size, and the larger shrimp are more fecund. Life stage parameters were obtained from the literature and are listed in Table 1.

Table D2. Life history parameters and sources for *Pandalus jordani*. Stages include eggs, larvae, juveniles and adults. Parameters include survival rates and duration times.

**Parameter**	**Value**	**Source**	**Notes**
e_{sp} = egg survival	0.9949043 day^{-1}	(Brillon et al. 2005)	This paper reports that eggs can be lost from females due to cannibalism by neighbouring shrimp, of being knocked off during scavenging (in Icelandic waters). As a result, egg mortality might be 'high' for this species.
e_{dp} = egg duration	152 days	(Modin and Cox 1967)	These authors just write that the species takes ~5 months for eggs to develop
l_{sp} = larval survival	0.9629563 day^{-1}	(Rothlisberg 1975)	Field based estimate was from sampling at different times and observing that ~1% or less made it to stage VII in each of the two years sampled (p.104 and Fig 28). This would give daily mortality of 0.01^(1/duration of larvae) = 0.9629563
l_{dp} = larval duration	122 days	(Rothlisberg 1975)	Fig 22 in this paper shows trends of larval stage over time, based on different temperatures. Larvae seem to reach their final stage after between 90-120 days, which I think is why they seem to write ~100 days later on. (Table XI has actual values for time and it is 96 or 111 days for temp 14 and 11 C respectively)
j_{sp} = juvenile survival	0.9958988 day^{-1}	(Rothlisberg 1975)	Rothlisberg (1975) uses the value 1.5, and so do Rothlisberg and Miller (1983). This translates to 0.223 total – and seems to represent 1.5 years, giving 0.223^(1/537) for daily survival. And so monthly survival = $(1.5^{(1/537)})^{30}$ = 0.92102

j_{dp} = juvenile duration	457 days	(Rothlisberg 1975)	Rothlisberg (1975) writes that there are ~18 months between hatching and commercial recruitment (hence 18-larvalduration). However, Hannah writes that the juveniles recruit to the fishery after a few months of the season starting, so juveniles would be around ~12 months
a_{sp} = adult survival	Winter mortality =0.9970535 day^{-1} Summer mortality = 0.9980347 day^{-1}<br><br>Weighted survival = 0.9975717 day^{-1} (assuming summer is 5 months and winter 7)	(Hannah 1995, Gallagher et al. 2004)	Gallagher et al. (2004) believe there is a difference in summer and winter mortalities due to differing levels of predation. Winter mortality is for Nov-March and summer is April-October, when Pacific whiting are migrating north and consume pink shrimp.<br>Hannah (1995) found range in monthly natural mortalities of 0.03-0.17.<br>RANGES in mortality rates used by Gallagher were Mw 0.03-0.10 and Ms = 0.04-0.12 (using the SD from Hannah's results)
f_{ap} = adult fecundity	3.425 eggs day^{-1}	(Hannah 1995)	The number of eggs seems to depend on the size of the shrimp (Hannah 1995). Smaller individuals produce ~2000 eggs and larger ones produce ~3000 eggs. This is why I am trying to decide between a model with one adult stage, that produces ~2500 eggs or two that produce 2000 and 3000 eggs respectively.<br>The number of eggs is divided by 12 to get monthly rates and then by 2 to get female-only egg production.<br>With one adult stage – lambda will be larger, because the first year they will produce 2500 and the number that survive to the next year will make the same amount, but in that first year they will have contributed 2500 and not 2000

Transition and Elasticity Matrices:

Using values in Table C2 the final transition matrix is:

	Eggs	Larvae	Juvenile	Adults

Eggs	0.9906	0	0	3.4247
Larvae	0.0043	0.9626	0	0
Juvenile	0	0.0004	0.9952	0
Adults	0	0	0.0007	0.9976

In order to ensure a null growth rate, $\lambda = 1$, all values in the transition matrix were multiplied by the multiplier 1.000011.

The output was the following elasticity matrix:

	Eggs	Larvae	Juvenile	Adults
Eggs	0.1395	0	0	0.0013
Larvae	0.0013	0.0342	0	0
Juvenile	0	0.0013	0.2734	0
Adults	0	0	0.0013	0.5477
**Sums**	**0.1408**	**0.0355**	**0.2747**	**0.5490**

Pacific Hake, **Merluccius productus**

This model is female only, as the model is taken from Smith (1995) and provides numbers of surviving female embryos produced per individual per day. The Lefkovitch matrix that is provided by Smith (1995) includes two larval stages and four juvenile stages. In order to finish our model with only one elasticity for each life stage (eggs, larvae, juveniles, pre-recruits and adults) we combined the estimates for larval and juvenile stages into 1, through waiting survivals by duration of each sub-stage. This model is also on a daily time step since that is what Smith (1995) used and as a result all survival rates are shown in daily rates.

Table D3. Pacific hake model parameter values. Note that for all of this is Smith (1995) Table 4, there is also some information in Hollowed (1992) but Smith was the main source used.

**Parameter**	**Value**	**Citation**
e_{sh} = egg survival	0.865 day^{-1}	(Hollowed 1992)
e_{dh} = egg duration	16 days	(Smith 1995)
l_{sh} = larval survival	0.9325446 day^{-1}	(Smith 1995)
l_{dh} = larval duration	112 days	(Smith 1995)
j_{sh} = juvenile survival	0.9955708 day^{-1}	(Smith 1995)
j_{dh} = juvenile duration	602 days	(Smith 1995)
pr_{sh} = pre-recruit survival	0.999452 day^{-1}	(Smith 1995)
pr_{dh} = pre-recruit duration	730 days	(Smith 1995)
a_{sh} = adult survival	0.999452 day^{-1}	(Smith 1995)
f_{prh} = pre-recruit fecundity	10.5 surviving female embryos/female/d (Smith gives it as sfe/f/d)	(Smith 1995)
f_{ah} = adult fecundity	199.2 sfe/female/d	(Smith 1995)

Transition and Elasticity Matrices:

The transition matrix using parameter values from Table D3 is:

	Eggs	Larvae	Juvenile	Pre-recruit	Adults
Eggs	0.8503	0	0	10.5000	199.2000
Larvae	0.0147	0.9325	0	0	0
Juvenile	0	0	0.9952	0	0
Pre-recruit	0	0	0.0003	0.9983	0
Adults	0	0	0	0.0011	0.9995

In order to ensure a null growth rate, $\lambda = 1$, all values in the transition matrix were multiplied by the multiplier 1.000133.

Elasticity matrix:

	Eggs	Larvae	Juvenile	Pre-recruit	Adults
Eggs	0.0017	0	0	0	0.0003
Larvae	0.0003	0.0043	0	0	0
Juvenile	0	0.0003	0.0661	0	0
Pre-recruit	0	0	0.0003	0.2007	0
Adults	0	0	0	0.0003	0.7256
SUMS	0.0021	0.0046	0.0665	0.2010	0.7259

Krill,* Euphausia pacifica*—Model Methods and Parameters

The model for *Euphausia pacifica* is a stage structured model that includes eggs, larvae, juveniles, pre-recruits and adults. Detailed durations and survivals are available for the larval stages of nauplius, calyptopes and furcilia (Rumsey and Franks 1999, Bi et al. 2011) but for our purposes these were lumped into one larval stage. The model groups males and females together assuming the same parameter values for both.

E. pacifica has biological characteristics requiring a slightly different approach from the other models. Depending on when eggs are spawned, there seems to be some evidence that the juveniles mature after a different number of months (pers comm. Julie Keister, (Harvey et al. 2010)). Brinton (1976) notes that full size was reached after 7 months for early year recruits and about 1 year for summer recruits. If spawned in early spring, then they may mature into pre-recruits and thus start spawning after only a few (2) months. However if they are spawned towards the end of the season, then they will likely overwinter as juveniles and mature in the following spring (5 month juvenile stage). As a result we developed two separate models to investigate the different conclusions that would be reached depending duration of the juvenile stage. The two models therefore only differed in their juvenile durations. Brinton (1976) did note that survival is lower for those spawned earlier in the year, however here we assume them to be constant. Different exposures were calculated for each krill 'type' based on the timing of when each spawning population is present in the water column.

Table D4. Life history stages included in the model for *E. pacifica*. Including survival rates and duration times with sources provided. Note: there are two alternative sources for early life stage parameter values – models have been tested for both and show little difference in population growth rate.

Parameter	Value (daily values)	Source	Notes
e_{se} = egg survival	0.91	(Heath 1977, Bi et al. 2011)*	Bi et al. (2011) estimates an egg mortality rate of 9% per day. Heath estimated a lower value at 6% per day, but the more recent estimate was used.
e_{de} = egg duration	2 days	(Bi et al. 2011)	Eggs are present for 2 days before hatching
l_{se} = larval survival	0.9756061 day^{-1}	(Bi et al. 2011)	From Bi et al. (2011) there are estimates of the durations of larval stages nauplii, furcilia and calyptopes – these were combined into one larval survival using weighted sum based on the durations of each of the stages
l_{de} = larval duration	66 days	(Bi et al. 2011)	Total number of days nauplii, fucilia and calyptopes are present in the water column
j_{se} =	0.9867395	(Brinton 1976)	

juvenile survival			
je_{de} = juvenile duration for early spawners	61	(Brinton 1976, Harvey et al. 2010)	Brinton (1976) suggests that juveniles are only present for 2 months, and Harvey et al. (2010) writes that *pacifica* can recruit to spawning size after 4-7 months in the water column. With ~2 months in the egg and larval stages this means that on the lower end juveniles are present for 2 months and the higher end 5 months.
jl_{de} = juvenile duration for late spawners	152	(Brinton 1976, Harvey et al. 2010)	For this model with the longer juvenile stage, the higher end of 5 months was used.
pr_{se} = pre-recruit survival	0.9852339	(Brinton 1976)	
pr_{de} = pre-recruit duration	91 days	Based on information in (Harvey et al. 2010)	Harvey et al. (2010) shows that brood size can increase almost three-fold for smaller vs. larger krill. To account for the fact that brood sizes increase with body size, we included a pre-recruit stage. This stage was determined to be approximately 3 months as in Harvey et al. (2010) there is a ~100 day period when smaller brood sizes have been observed.
a_{se} = adult survival	0.9831166	(Brinton 1976)	
f_{pre} = pre-recruit fecundity	$f_{ap}/3$	(Feinberg et al. 2007)	Feinberg et al. (2007) report differing fecundity values based on body size, however from personal communication with Julie Keister, she suggested that pre-recruits have spawning output that is almost the same as adults and is only slightly lower during this life stage. Therefore the fecundity is assumed to be 90% of adult fecundity.
f_{ae} = adult fecundity	7.15	(Feinberg et al. 2013)	These authors report three different daily fecundity estimates for *E. pacifica* in the California Current, from these we calculated

			total fecundity based on length of spawning season, averaged the three and obtained a daily estimate of fecundity for the entire year.

* Note: there are two alternative sources for early life stage parameter values – models have been tested for both and show little difference in population growth rate.

Transition and Elasticity Matrices:

Early spawners (shorter juvenile life stage) transition matrix from parameters in Table C4:

	Eggs	Larvae	Juvenile	Pre-recruit	Adults
Eggs	0.4764	0	0	2.3845	7.1534
Larvae	0.4336	0.9697	0	0	0
Juvenile	0	0.0059	0.9762	0	0
Pre-recruit	0	0	0.0105	0.9801	0
Adults	0	0	0	0.0051	0.9831

Later spawners (longer juvenile life stage) transition matrix:

	Eggs	Larvae	Juvenile	Pre-recruit	Adults
Eggs	0.4764	0	0	2.3845	7.1534
Larvae	0.4336	0.9697	0	0	0
Juvenile	0	0.0059	0.9847	0	0
Pre-recruit	0	0	0.0020	0.9801	0
Adults	0	0	0	0.0051	0.9831

Multipliers for the two models, to ensure $\lambda = 1$, h_e= 0.9714854, h_l= 0.9885264.

The elasticity matrix for the early spawners:

	Eggs	Larvae	Juvenile	Pre-recruit	Adults
Eggs	0.0133	0	0	0.0115	0.0039
Larvae	0.0154	0.2502	0	0	0

Juvenile	0	0.0154	0.2828	0	0
Pre-recruit	0	0	0.0154	0.3064	0
Adults	0	0	0	0.0039	0.0819
SUMS	0.0287	0.2656	0.2982	0.3218	0.0858

The elasticity matrix for the late spawners:

	Eggs	Larvae	Juvenile	Pre-recruit	Adults
Eggs	0.0082	0	0	0.0060	0.0032
Larvae	0.0092	0.2136	0	0	0
Juvenile	0	0.0092	0.3386	0	0
Pre-recruit	0	0	0.0092	0.2874	0
Adults	0	0	0	0.0032	0.1120
SUMS	0.0175	0.2229	0.3478	0.2966	0.1152

Krill,* Thysanoessa spinifera*—Model Methods and Parameters

The model for *Thysanoessa spinifera* is a stage structured model that includes eggs, larvae, juveniles, and adults. Most model parameters for *E. pacifica* were used to fill in the many unknowns about this species, however since *E. pacifica* exhibits different larval duration than has been found for *T. spinifera* we decided to make a separate model for this species. Note that it does not include the sub-adult stage, as there was no mention of sub-adults in the Summers (1993) thesis which seems to be the most detailed outline of life history.

Table D5. Life history stages included in the model for *T. spinifera*. Survival rates and duration times are all the same as the *E. pacifica model* except where written in red. Sources provided.

**Parameter**	**Value** (daily values)	**Source**	**Notes**
e_{st} = egg survival	0.91	(Heath 1977, Bi et al. 2011)	Bi et al. (2011) estimates an egg mortality rate of 9% per day. Heath estimated a lower value at 6% per day, but the more recent estimate was used.
e_{dt} = egg duration	2 days	(Summers 1993, Bi et al. 2011)	Eggs are present for 2 days before hatching. Summers (1993) found this for *T. spinifera* as well.
l_{st} = larval survival	0.9756061 day^{-1}	(Bi et al. 2011)(Bi et al. 2011)(Bi et al. 2011)(Bi et al. 2011)	From Bi et al. (2011) there are estimates of the durations of larval stages nauplii, furcilia and calyptopes – these were combined into one larval survival using weighted sum based on the durations of each of the stages
l_{dt} = larval duration	66 days	(Summers 1993, Bi et al. 2011)	Total number of days nauplii, fucilia and calyptopes are present in the water column. Note that while Summers (1993) did not outline the durations of all larval stages, they approximated a total of 67 days from egg to juvenile, therefore the same value as *E. pacifica* was used.
j_{st} = juvenile survival	0.9867395	(Brinton 1976)	
j_{dt} = juvenile duration for	183	(Summers 1993)	Summers (1993) found that juveniles took approximately 6 months to develop into adults.
a_{st} = adult	0.9831166	(Brinton 1976)	

survival			
f_{at} = adult fecundity	7.15	(Feinberg et al. 2013)	These authors report three different daily fecundity estimates for *E. pacifica* in the California Current, from these we calculated total fecundity based on length of spawning season, averaged the three and obtained a daily estimate of fecundity for the entire year.

Transition and Elasticity Matrices:

T. spinifera transition matrix:

	Eggs	Larvae	Juvenile	Adults
Eggs	0.4764	0	0	7.1534
Larvae	0.4336	0.9697	0	0
Juvenile	0	0.0059	0.9835	0
Adults	0	0	0.0032	0.9831

Multiplier to ensure $\lambda = 1$, h_t= 0.9738514.

The elasticity matrix:

	Eggs	Larvae	Juvenile	Adults
Eggs	0.0129	0	0	0.0149
Larvae	0.0149	0.2531	0	0
Juvenile	0	0.0149	0.3387	0.0000
Adults	0	0	0.0149	0.3356
SUMS	0.0278	0.2680	0.3536	0.3505

Appendix E: Justification for uncertainty conclusions

Table E1. Justifications for uncertainty conclusions. Including uncertainty regarding consequence, exposure and population model, for each species' life stage.

Species	Life Stage	U_c	Justification *For Consequence Uncertainty*	U_e	Justification *For Exposure Uncertainty*
Metacarcinus magister	Eggs	3	No direct studies that were controlled	1	We know where adults are from fishing and eggs are on adults
	Larvae	2	Three recent studies from theses, however inconsistent results on survival impact indicate some uncertainty	3	Has been minimally studied
	Megalops	3	No direct studies, some observations from Miller, but conclusions from larvae	3	Has been minimally studied
	Juvenile i	3	No published studies, and only pilot experiments noted	1	We know where adults are and juveniles are found there
	Juvenile ii	3	No published studies, assumed same as other life stage as same species	1	Same as above
	Adult	1	Two studies have both shown strong capabilities of tolerating low pH	1	We know where adults are from fishing
Pendalus jordani	Eggs	2	Studies on species in the same genus with agreement	1	We know where adults are from fishing and eggs are on adults
	Larvae	2	Studies on species in the same genus with agreement	3	Has been minimally studied
	Juvenile	3	Studies on other life stages of species in same genus	1	We know where they settle
	Adult	2	Studies on species in the same genus with agreement	1	We know where adults are from fishing
Limacina helicina	Eggs & larvae	2	Based on consequence conclusions from juveniles and subadults, with expert opinion.	3	With a highly patchy distribution, thus is very hard to be conclusive where it is found and the timing of when they are in the water column is unclear
	Juvenile	1	Multiple papers show pteropod dissolution and negative consequences	3	Same as above

	Subadult	1	Multiple papers show pteropod dissolution and negative consequences	3	Same as above
	Adult	2	Based on consequence conclusions from juveniles and subadults, with expert opinion.	3	Same as above
Merluccius productus	Eggs	3	On other species not in same genus	1	Many years of CalCOFI surveys
	Larvae	3	On other species not in same genus	1	Many years of CalCOFI surveys
	Adult	1	Adult fish are robust	1	Adult distributions not mapped as consequence assume to be 1
Euphausia pacifica Early and late spawners	Eggs	2	Only one study on this species, and one from another – although results are in agreement	2	Somewhat well studied, we have a fairly good idea of their distribution
	Larvae	2	Only one study on this species life stage	2	Same as above
	Juveniles	3	Based on adults	2	Same as above
	Sub-adults	3	Based on adults	2	Same as above
	Adult	3	Only one paper on a different species in same genus and not very strong conclusions	2	Same as above
Thysanoessa spinifera	Eggs	3	Based on *E. pacifica*	2	Somewhat well studied, we have a fairly good idea of their distribution
	Larvae	3	Based on *E. pacifica*	2	Same as above
	Adult	3	Based on *E. pacifica*	2	Same as above

Appendices Literature Cited

Armstrong, D., C. Rooper, and D. Gunderson. 2003. Estuarine production of juvenile dungeness crab (cancer magister) and contribution to the Oregon-Washington coastal fishery. Estuaries **26**:1174-1188.

Arnberg, M., P. Calosi, J. Spicer, A. Tandberg, M. Nilsen, S. Westerlund, and R. Bechmann. 2012. Elevated temperature elicits greater effects than decreased pH on the development, feeding and metabolism of northern shrimp (Pandalus borealis) larvae. Marine Biology:1-12.

Bechmann, R. K., I. C. Taban, S. Westerlund, B. F. Godal, M. Arnberg, S. Vingen, A. Ingvarsdottir, and T. Baussant. 2011. Effects of ocean acidification on early life stages of shrimp (Pandalus borealis) and mussel (Mytilus edulis). J Toxicol Environ Health A **74**:424-438.

Bednaršek, N., R. A. Feely, J. C. P. Reum, B. Peterson, J. Menkel, S. R. Alin, and B. Hales. 2014. Limacina helicina shell dissolution as an indicator of declining habitat suitability owing to ocean acidification in the California Current Ecosystem. Proceedings of the Royal Society B: Biological Sciences **281**.

Bednaršek, N., G. A. Tarling, D. C. E. Bakker, S. Fielding, A. Cohen, A. Kuzirian, D. McCorkle, B. Lézé, and R. Montagna. 2012. Description and quantification of pteropod shell dissolution: a sensitive bioindicator of ocean acidification. Global Change Biology **18**:2378-2388.

Bi, H., L. Feinberg, C. T. Shaw, and W. T. Peterson. 2011. Estimated development times for stage-structured marine organisms are biased if based only on survivors. Journal of Plankton Research **33**:751-762.

Bignami, S., I. C. Enochs, D. P. Manzello, S. Sponaugle, and R. K. Cowen. 2013. Ocean acidification alters the otoliths of a pantropical fish species with implications for sensory function. Proceedings of the National Academy of Sciences **110**:7366-7370.

Brillon, S., Y. Lambert, and J. Dodson. 2005. Egg survival, embryonic development, and larval characteristics of northern shrimp (Pandalus borealis) females subject to different temperature and feeding conditions. Marine Biology **147**:895-911.

Brinton, E. 1976. Population biology of *Euphausia pacifica* off Southern California. Fishery Bulletin **74**:733-762.

Bromhead, D., V. Scholey, S. Nicol, D. Margulies, J. Wexler, M. Stein, S. Hoyle, C. Lennert-Cody, J. Williamson, J. Havenhand, T. Ilyina, and P. Lehodey. The Potential Impact of Ocean Acidification Upon Eggs and Larvae of Yellowfin Tuna (Thunnus albacares). Deep Sea Research Part II: Topical Studies in Oceanography.

Busch, D. S., M. Maher, P. Thibodeau, and P. McElhany. 2014. Shell Condition and Survival of Puget Sound Pteropods Are Impaired by Ocean Acidification Conditions. Plos One **9**.

CalCOFI. 2012. Pacific hake egg and larval survey 1984-2012.*in* C. C. O. F. Investigation, editor., http://www.calcofi.org/new.data/.

Cass-Calay, S. L. 2003. The feeding ecology of larval Pacific hake (Merluccius productus) in the California Current region: an updated approach using a combined OPC/MOCNESS to estimate prey biovolume. Fisheries Oceanography **12**:34-48.

Christmas, A.-M. F. 2013. Effects of ocean acidification on dispersal behavior in the larval stage of the Dungeness crab and the Pacific Green Shore crab. Western Washington University.

Dahlstrom, W. A. 1970. Synopsis of biological data on the ocean shrimp Pandalus jordani Rathbun. FAO Fisheries Report **57**:377-1416.

Dahlstrom, W. A. 1973. Status of the California ocean shrimp resource and its management. Marine Fisheries Review **35**:55-59.

Descoteaux, R. 2014. Effects of ocean acidification on development of Alaskan crab larvae. University of Alaska Fairbanks.

Feinberg, L., and W. Peterson. 2003. Variability in duration and intensity of euphausiid spawning off central Oregon, 1996–2001. Progress in Oceanography **57**:363-379.

Feinberg, L. R., W. T. Peterson, and C. Tracy Shaw. 2010. The timing and location of spawning for the Euphausiid Thysanoessa spinifera off the Oregon coast, USA. Deep Sea Research Part II: Topical Studies in Oceanography **57**:572-583.

Feinberg, L. R., C. Shaw, and W. T. Peterson. 2007. Long-term laboratory observations of Euphausia pacifica fecundity: comparison of two geographic regions. Marine Ecology Progress Series **341**:141-152.

Feinberg, L. R., C. T. Shaw, W. T. Peterson, M. Décima, Y. Okazaki, and S.-J. Ju. 2013. Euphausia pacifica brood sizes: a North Pacific synthesis. Journal of Plankton Research.

Frommel, A., A. Schubert, U. Piatkowski, and C. Clemmesen. 2013. Egg and early larval stages of Baltic cod, Gadus morhua, are robust to high levels of ocean acidification. Marine Biology **160**:1825-1834.

Frommel, A. Y., R. Maneja, D. Lowe, C. K. Pascoe, A. J. Geffen, A. Folkvord, U. Piatkowski, and C. Clemmesen. 2014. Organ damage in Atlantic herring larvae as a result of ocean acidification. Ecological Applications **24**:1131-1143.

Gallagher, C. M., R. W. Hannah, and G. Sylvia. 2004. A comparison of yield per recruit and revenue per recruit models for the Oregon ocean shrimp, Pandalus jordani, fishery. Fisheries Research **66**:71-84.

Hammer, K. M. 2012. Acid-base regulation and metabolite responses in shallow-and deep-living marine invertebrates during environmental hypercapnia. Norwegian University of Science and Technology.

Hankin, D. G., N. Diamond, M. S. Mohr, and J. Ianelli. 1989. Growth and reproductive dynamics of adult female Dungeness crabs (Cancer magister) in northern California. Journal du Conseil: ICES Journal of Marine Science **46**:94-108.

Hannah, R. W. 1995. Variation in geographic stock area, catchability, and natural mortality of ocean shrimp (Pandalus jordani): some new evidence for a trophic interaction with Pacific hake (Merluccius productus). Canadian Journal of Fisheries and Aquatic Sciences **52**:1018-1029.

Hannah, R. W. 2011. Variation in the distribution of ocean shrimp (Pandalus jordani) recruits: links with coastal upwelling and climate change. Fisheries Oceanography **20**:305-313.

Hans, S., S. Fehsenfeld, J. Treberg, and D. Weihrauch. 2014. Acid–base regulation in the Dungeness crab (Metacarcinus magister). Marine Biology:1-15.

Harvey, H. R., S.-J. Ju, S. K. Son, L. R. Feinberg, C. T. Shaw, and W. T. Peterson. 2010. The biochemical estimation of age in Euphausiids: Laboratory calibration and field comparisons. Deep Sea Research Part II: Topical Studies in Oceanography **57**:663-671.

Heath, W. A. 1977. The ecology and harvesting of euphausiids in the strait of georgia. University of British Columbia.

Higgins, K. 1997. Stochastic Dynamics and Deterministic Skeletons: Population Behavior of Dungeness Crab. Science **276**:1431-1435.

Higgins, K., A. Hastings, J. N. Sarvela, and L. W. Botsford. 1997. Stochastic Dynamics and Deterministic Skeletons: Population Behavior of Dungeness Crab. Science **276**:1431-1435.

Hobbs, R., and L. Botsford. 1992. Diel vertical migration and timing of metamorphosis of larvae of the Dungeness crab Cancer magister. Marine Biology **112**:417-428.

Hobbs, R. C., L. W. Botsford, and A. Thomas. 1992. Influence of hydrographic conditions and wind forcing on the distribution and abundance of Dungeness crab, Cancer magister, larvae. Canadian Journal of Fisheries and Aquatic Sciences **49**:1379-1388.

Hollowed, A. B. 1992. Spatial and temporal distributions of Pacific hake, Merluccius productus, larvase and estimates of survival during early life stages California Cooperative Oceanic Fisheries Investigations Reports **33**:100-123.

Kawaguchi, S., A. Ishida, R. King, B. Raymond, N. Waller, A. Constable, S. Nicol, M. Wakita, and A. Ishimatsu. 2013. Risk maps for Antarctic krill under projected Southern Ocean acidification. Nature Clim. Change **advance online publication**.

Kawaguchi, S., H. Kurihara, R. King, L. Hale, T. Berli, J. P. Robinson, A. Ishida, M. Wakita, P. Virtue, S. Nicol, and A. Ishimatsu. 2011. Will krill fare well under Southern Ocean acidification? Biol Lett **7**:288-291.

Kroeker, K. J., R. L. Kordas, R. N. Crim, and G. G. Singh. 2010. Meta-analysis reveals negative yet variable effects of ocean acidification on marine organisms. Ecology Letters **13**:1419-1434.

Long, W. C., K. M. Swiney, C. Harris, H. N. Page, and R. J. Foy. 2013. Effects of Ocean Acidification on Juvenile Red King Crab (Paralithodes camtschaticus) and Tanner Crab (Chionoecetes bairdi) Growth, Condition, Calcification, and Survival. PLoS ONE **8**:e60959.

Lu, B., D. L. Mackas, and D. F. Moore. 2003a. Cross-shore separation of adult and juvenile euphausiids in a shelf-break alongshore current. Progress in Oceanography **57**:381-404.

Lu, B., D. L. Mackas, and D. F. Moore. 2003c. Cross-shore separation of adult and juvenile euphausiids in a shelf-break alongshore current. Progress in Oceanography **57**:381-404.

McKelvey, R., D. Hankin, K. Yanosko, and C. Snygg. 1980. Stable cycles in multistage recruitment models: an application to the northern California Dungeness crab (Cancer magister) fishery. Canadian Journal of Fisheries and Aquatic Sciences **37**:2323-2345.

Melzner, F., M. Gutowska, M. Langenbuch, S. Dupont, M. Lucassen, M. C. Thorndyke, M. Bleich, and H.-O. Pörtner. 2009. Physiological basis for high CO 2 tolerance in marine ectothermic animals: pre-adaptation through lifestyle and ontogeny? Biogeosciences Discussions **6**:4693-4738.

Miller, J. 2015. Effect of low pH on early life stages of the decapod crustacean, Dungeness crab (Cancer magister). University of Washington.

Modin, J. C., and K. W. Cox. 1967. Post-Embryonic Development of Laboratory-Reared Ocean Shrimp, Pandalus jordani Rathbun. Crustaceana **13**:197-219.

Moloney, C., L. Botsford, and J. Largier. 1994. Development, survival and timing of metamorphosis of planktonic larvae in a variable environment: the Dungeness crab as an example. Marine ecology progress series. Oldendorf **113**:61-79.

Moser, H. G., N. C. H. Lo, and P. E. Smith. 1997. Vertical distribution of Pacific hake eggs in relation to stage of development and temperature. California Cooperative Oceanic Fisheries Investigations Report:120-126.

Munday, P. L., J. M. Donelson, D. L. Dixson, and G. G. K. Endo. 2009. Effects of Ocean Acidification on the early Life History of a Tropical Marine Fish. Proceedings: Biological Sciences **276**:3275-3283.

Pane, E. F., and J. P. Barry. 2007. Extracellular acid-base regulation during short-term hypercapnia is effective in a shallow-water crab, but ineffective in a deep-sea crab. Marine Ecology Progress Series **334**:1-9.

Pauley, G. B., D. A. Armstrong, T. W. Heun, U. S. A. E. W. E. S. C. E. Group, C. National Wetlands Research, U. S. Fish, Wildlife, U. S. Fish, R. Wildlife, Development, and S. United States. Army. Corps of Engineers. Waterways Experiment. 1986. Species profiles : life histories and environmental requirements of coastal fishes and invertebrates (Pacific Northwest) : dungeness crab. Fish and Wildlife Service ; Coastal Ecology Group, Waterways Experiment Station, Washington, DC; Vicksburg, MS.

Rasmuson, L. K. 2013. The Biology, Ecology and Fishery of the Dungeness crab, Cancer magister. Adv Mar Biol **65**:95-148.

Reed, P. H. 1969. Culture methods and effects of temperature and salinity on survival and growth of Dungeness crab (Cancer magister) larvae in the laboratory. Journal of the Fisheries Board of Canada **26**:389-397.

Reilly, P. N. 1983. Dynamics of Dungeness crab, Cancer magister, larvae off central and northern California. Life history, environment, and mariculture studies of Dungeness crab, Cancer magister, with emphasis on the central California® fishery resource. Calif. Dept Fish Game, Fish. Bull **172**:57-84.

Rothlisberg, P., and W. G. Pearcy. 1976. An epibenthic sampler used to study the ontogeny of vertical migration of pandalus dordani (Decapoda caridea).

Rothlisberg, P. C. 1975. Larval ecology of Pandalus jordani Rathbun. Oregon State University.

Rothlisberg, P. C., and C. B. Miller. 1983. *Factors affectin*g the distribution, abundance and survival of Pandalus jordani (Decapoda, Pandalidae) larvae off the Oregon coast. Fisheries Bulletin **81**:455-472.

Rumrill, S. S. 1990. Natural mortality of marine invertebrate larvae. Ophelia **32**:163-198.

Rumsey, S. M., and P. J. S. Franks. 1999. Influence of variability in larval development on recruitment success in the euphausiid Euphausia pacifica: elasticity and sensitivity analyses. Marine Biology **133**:283-291.

Saba, G. K., O. Schofield, J. J. Torres, E. H. Ombres, and D. K. Steinberg. 2012. Increased Feeding and Nutrient Excretion of Adult Antarctic Krill, Euphausia superba, Exposed to Enhanced Carbon Dioxide (CO2). PLoS ONE **7**:1-12.

Shirley, S. M., T. C. Shirley, and S. D. Rice. 1987. Latitudinal variation in the Dungeness crab, Cancer magister: zoeal morphology explained by incubation temperature. Marine Biology **95**:371-376.

Smith, P. E. 1995. Development of the population biology of the Pacific hake, Merluccius productus. California Cooperative Oceanic Fisheries Investigations Reports **36**:144-152.

Stevens, B. G., and D. A. Armstrong. 1984. Distribution, abundance, and growth of juvenile Dungeness crabs, Cancer magister, in Grays Harbor estuary, Washington. Fishery Bulletin **82**.

Summers, P. L. 1993. Life history, growth and aging in Thysanoessa spinifera. University of Victoria.

Vance, P., J. Keister, and W. Peterson. 2003. Seasonal and annual variation in the population composition and depth distributions of the euphausiid, Euphausia pacifica. Eos Transactions, AGU **84**:52.

Wainwright, T., D. Armstrong, P. Dinnel, J. Orensanz, and K. McGraw. 1992. Predicting effects of dredging on a crab population: An equivalent adult loss approach. Fishery Bulletin **90**:171-182.

Wang, K. 2014. The life cycle of the pteropod Limacina helicina in Rivers Inlet University of British Columbia.

Wickham, D. 1979a. The relationship between megalopae of the Dungeness Crab, Cancer magister, and the hydroid, Velella velella, and its influence on abundance estimates of C. magister megalopae. California Fish and Game **65**:184-186.

Wickham, D. E. 1979c. Predation by the nemertean Carcinonemertes errans on eggs of the Dungeness crab Cancer magister. Marine Biology **55**:45-53.
